# The representation of Indigenous peoples in chronic disease clinical trials in Australia, Canada, New Zealand, and the United States

**DOI:** 10.1177/17407745211069153

**Published:** 2022-01-06

**Authors:** Valerie Umaefulam, Tessa Kleissen, Cheryl Barnabe

**Affiliations:** Departments of Medicine and Community Health Sciences, Cumming School of Medicine, University of Calgary, Calgary, AB, Canada

**Keywords:** Clinical trials, Indigenous people, Indigenous health, chronic disease, health disparities

## Abstract

**Background:**

Indigenous peoples are overrepresented with chronic health conditions and experience suboptimal outcomes compared with non-Indigenous peoples. Genetic variations influence therapeutic responses, thus there are potential risks and harm when extrapolating evidence from the general population to Indigenous peoples. Indigenous population–specific clinical studies, and inclusion of Indigenous peoples in general population clinical trials, are perceived to be rare. Our study (1) identified and characterized Indigenous population–specific chronic disease trials and (2) identified the representation of Indigenous peoples in general population chronic disease trials conducted in Australia, Canada, New Zealand, and the United States.

**Methods:**

For Objective 1, publicly available clinical trial registries were searched from May 2010 to May 2020 using Indigenous population–specific terms and included for data extraction if in pre-specified chronic disease. For identified trials, we extracted Indigenous population group identity and characteristics, type of intervention, and funding type. For Objective 2, a random selection of 10% of registered clinical trials was performed and the proportion of Indigenous population participants enrolled extracted.

**Results:**

In total, 170 Indigenous population–specific chronic disease trials were identified. The clinical trials were predominantly behavioral interventions (n = 95). Among general population studies, 830 studies were randomly selected. When race was reported in studies (n = 526), Indigenous individuals were enrolled in 172 studies and constituted 5.6% of the total population enrolled in those studies.

**Conclusion:**

Clinical trials addressing chronic disease conditions in Indigenous populations are limited. It is crucial to ensure adequate representation of Indigenous peoples in clinical trials to ensure trial data are applicable to their clinical care.

## Introduction

Inequities in health outcomes and experiences in the health system exist for certain population groups due to unfair and avoidable differences in determinants of health. The term “Indigenous” in this article refers to the original peoples of Australia, Canada, New Zealand, and the United States and their descendants who share a similar history of colonization and its detrimental impact on health.^1^ Indigenous peoples are disproportionately affected by chronic conditions, and these inequities may increase risk for chronic disease, and influence presentation, characteristics, and outcomes. For instance, type 2 diabetes mellitus is more prevalent in Native American populations than the general population and is more likely to result in impaired kidney function.^2^ Indigenous populations in Australia, Canada, and the United States are overrepresented with inflammatory arthritis and experience higher disability and mortality rates compared with non-Indigenous populations.^3^ Colonization events instigate and maintain inequities in social determinants of health; however, there are genetic variations contributing to disease risk and treatment efficacy differences.^4^ Furthermore, racial identities are known to influence clinical presentations and therapeutic responses.^5^ For example, a Canadian Early Arthritis Cohort observational study demonstrated that despite similar disease activity at treatment initiation and with applying the same therapy for early rheumatoid arthritis as White patients, Aboriginal patients were 60% less likely to achieve remission. This may be related to poor prognostic factors influenced by inequities of determinants of health, but the alternative explanation is that the rheumatoid arthritis medications were not as efficacious.^6^

Randomized controlled trials (RCTs) are regarded as providing the highest level of evidence for evaluating intervention efficacy and allows for the effectiveness of an intervention to be determined while controlling for confounders of the effect.^1,7^ Data from clinical trials are essential for testing the safety and efficacy of potential new therapies and interventions, and results are translated into practice for patient benefit.^8^ Although Indigenous peoples have a high burden of chronic conditions, they are perceived to be underrepresented in clinical trial research.^2^ Results obtained in other population groups are being extrapolated to Indigenous patients, which may not be appropriate.^9^ Treatment effectiveness may differ between Indigenous and non-Indigenous peoples; thus their inclusion in clinical trials can identify both benefits and harms and improve informed and shared decision-making for treatment decisions. Therefore, underrepresentation of Indigenous peoples in clinical trials has serious implications for medical science by limiting validity and generalizability of research findings, contributing to inequity in treatment outcomes,^8^ and influencing the allocation of resources for services and research.^10^

Reporting and enrollments of ethnic minority groups in clinical trials have been reported in the past.^11^ A previous systematic review from 1999 found there were limited well-designed clinical trials addressing medical interventions and health needs of Indigenous Australians.^12^ Another systematic review examined clinical trials on health-related issues in Indigenous communities of Canada, the United States, Australia, and New Zealand published from 1999 to 2010.^13^ The trials identified addressed mental health issues, diabetes, obesity, and parenting, but the study was limited by the inability to identify unpublished studies.^13^ Hunter et al.^14^ explored clinical trial registrations for the Australian Indigenous population from 2008 to 2018 via the Australian New Zealand Clinical Trials Registry (ANZCTR) and the US National Library of Medicine (NLM) clinicaltrials.gov. Trials were predominantly on topics in public health, mental health, or cardiovascular-disease related, and represented just 1.5% of all trials registered during that time period.^14^ None of the previous studies mentioned focused on identifying published and unpublished chronic disease-related clinical trials. This article builds on these studies by expanding the search and extraction methods to gain insights into the representation of Indigenous peoples in chronic disease trials in Australia, Canada, New Zealand, and the United States. Our study has two objectives: to identify and characterize Indigenous population–specific chronic disease trials, and to summarize the representation of Indigenous peoples in general population chronic disease clinical trials conducted in Australia, Canada, New Zealand, and the United States.

## Methods

### Methodologic approach

Environmental scan methodology was utilized in this study because it is a valuable tool to systematize and synthesize knowledge and can provide evidence for strategic action, decision-making, health policy, and program planning.^15^ We conducted our scan in June 2020 of registered Indigenous population–specific chronic disease trials in two registries; the US National Library of Medicine (NLM) clinical trial registry (https://clinicaltrials.gov) and the Australian New Zealand Clinical Trials Registry (ANZCTR) (https://www.anzctr.org.au). The NLM clinical trial registry consists of privately and publicly funded studies that explore studies in 50 US states and in 216 countries (including Canada, which does not have its own registry), and the ANZCTR is a primary registry of clinical trials carried out in Australia, New Zealand, and in other locations. Institutional review board approval and informed consent were not obtained, given that the study was an environmental scan of publicly available information. The research team comprises members of an epidemiology and health services research lab working to resolve care disparities experienced by Indigenous patients and consists of a member (C.B.) of the Métis Nation of Alberta. Two researchers (V.U. and C.B.) independently conducted the searches and identified the studies, and all three investigators extracted the data.

### Identification of studies

#### Indigenous population–specific trials

Clinical trials registries from May 2010 to May 2020 were searched for Indigenous population–specific terms (Table 1) in the title, abstract, or in the protocol. Key terms used to describe Indigenous peoples in Australia, Canada, New Zealand, and the United States were included.^16^ Some search terms included are now recognized as racist, but were used historically in the literature and thus were retained. Eligible studies were then included if conducted specifically in a pre-specified chronic condition of interest related to known overrepresentation of disease incidence or prevalence, or more severe outcomes, in the Indigenous population of the four countries of interest.^17,18^ These included arthritis, diabetes, hypertension, cardiovascular disease, mental illness, respiratory disease, kidney disease, and dental/periodontal disease. Cancer was not included given variabilities within cancer types and prognoses.^19,20^ The terms listed in Table 2 were used to identify the chronic conditions of interest. For each country, we conducted separate searches with individual chronic disease terms, general Indigenous population terms, and country-specific Indigenous population terms. Table 3 provides more information on how the terms were entered in the advanced search function of the databases to conduct the search.

**Table 1.** Indigenous population search terms.

		Search terms
General Indigenous population terms		Aboriginal, Indigenous, Tribe, Tribes, Tribal, Native People
Country-specific population terms	Australia	Aborigine, Torres Strait islander
	Canada	First Nation, First Nations, Indian, Indians, Métis, Half-Breed, Inuit, Eskimo
	New Zealand	Maori, Pacific Islander or Pacific People, Pasifika
	US	American Indian, Amerindian, Native American, Alaska Native, Native Hawaiian

**Table 2.** Pre-specified chronic disease conditions of interest.

Chronic disease	Search terms
Arthritis	Arthritis, osteoarthritis, inflammatory arthritis, rheumatoid arthritis, psoriatic arthritis, ankylosing spondylitis, rheumatic diseases, connective tissue disease, systemic lupus erythematosus, lupus, scleroderma, vasculitis
Diabetes	Diabetes, diabetes mellitus, diabetes complications
Hypertension	Hypertension, high blood pressure
Cardiovascular Disease	Heart disease, myocardial ischemia, myocardial infarction, congestive heart failure, vascular diseases, stroke, cerebrovascular accident, atherosclerosis, hypercholesterolemia
Mental illness	Mental disorders, anxiety disorders, mood disorders, depression, substance-related disorders, trauma and stressor-related disorders, suicide ideation, schizophrenia, loneliness, and gambling
Respiratory disease	Respiratory tract disease, lung disease, asthma, COPD, chronic obstructive pulmonary disease, chronic obstructive lung disease, emphysema, chronic bronchitis, bronchiectasis
Kidney disease	Chronic kidney disease, renal disease, nephritis, post-transplant
Dental/periodontal disease	Dental disease, tooth disease, mouth disease, periodontal disease

COPD: chronic obstructive pulmonary disease.

**Table 3.** Search strategy in registries.

Registries	Sections in advanced search^a^	Example of search combination^b^
*Clinicaltrials.gov*	Condition or disease	Diabetes
	Other terms	Aboriginal
	Study type	Interventional studies (Clinical Trials
	Country	Canada
	Study start	1 May 2010 to 31 May 2020
*ANZCTR*	Registry	ANZCTR
	Description of intervention(s)/exposure	Maori
	Study type	Interventional
	Health condition(s) or problem(s) studied	Systemic lupus erythematosus
	Registration date	1 May 2010 to 31 May 2020
	Countries of recruitment (AND)	New Zealand

a No selections were made in the other search rows.

b We conducted separate searches with a combination of chronic disease terms, general Indigenous population terms and country-specific Indigenous population terms.

#### Indigenous enrolment in general population clinical trials

We searched any clinical trials registered between May 2010 to May 2020 in the four countries using the pre-specified chronic diseases terms (Table 2) mentioned in the title, abstract, or protocol. The studies selected were listed in both registries as having completed recruitment and for the clinicaltrial.gov registry, with submitted results, in the aforementioned time period. Indigenous population–specific studies previously identified were excluded. All identified studies from the registries were collated and sorted based on the type of intervention, specified in the following section, to ensure sufficient sample of each study type. Ten percent of the studies from each intervention type were randomly selected for data extraction using an online random integer set generator, to identify the proportion of enrolment that was Indigenous. We included studies that were conducted in at least one of the chronic conditions, and conducted the searches in each registry, eliminating the duplicate of a study that was already randomly included in the first databases.

### Data collection

Indigenous population–specific trials: for each registered trial meeting inclusion criteria, we extracted details on Indigenous population group identity and geographic location(s) of research, chronic condition, participant age category (i.e. child, adult, or both), gender, type of intervention, study design, number of Indigenous participants enrolled, community involvement if described (which is an essential aspect of Indigenous research ethics), and funding type. We contacted investigators associated with the studies selected and searched Medline and Embase to identify publications for these studies in peer-reviewed journals so as to acquire any further details on demographic information.

Indigenous enrolment in general population clinical trials: for each selected study, we obtained details of the geographic location(s) of research, chronic condition studied, type of intervention, total number of participants enrolled, total number of Indigenous participants, study design, and funding type. For studies with no information in the registry, we contacted the authors/investigators associated with studies selected and searched for peer-reviewed publications via Medline and Embase, and extracted Indigenous population information from the study’s demographic tables. We searched Medline and Embase using the registration number and principal investigator name(s) as keywords. The linked publications feature in the registries was also used to retrieve publications related to the study.

For both objectives, in the clinicaltrial.gov registry, data were extracted using the comma-separated values (CSV) format and in ANZCTR, using the Excel format, and we viewed the full record of selected studies to extract further information. The study design was categorized as listed in the registry based on the allocation and intervention mode/assignment. The location data extracted was centered on the recruitment site. The intervention-type data collected was grounded on how it was classified in the registry (behavioral (including education, training),^21^ drug, device, procedure, other). For clinicaltrial.gov, we extracted the intervention/treatment type listed in the registry, while for ANZCTR, we obtained the information listed in the study’s intervention code. Information on community collaboration was extracted from the entries in the registries and from the publications obtained. We collected both the anticipated and actual enrollment data as indicated in the registry and/or study publication, but the final accrual data were used in the analysis. From the publications of studies identified, we extracted demographic information on the participants included in the study’s primary analysis report. Where discrepancies existed between the information obtained from the registry and the published data, we reported the data from the published paper.

## Results

### Characteristics of Indigenous-specific trials

**Figure 1.**
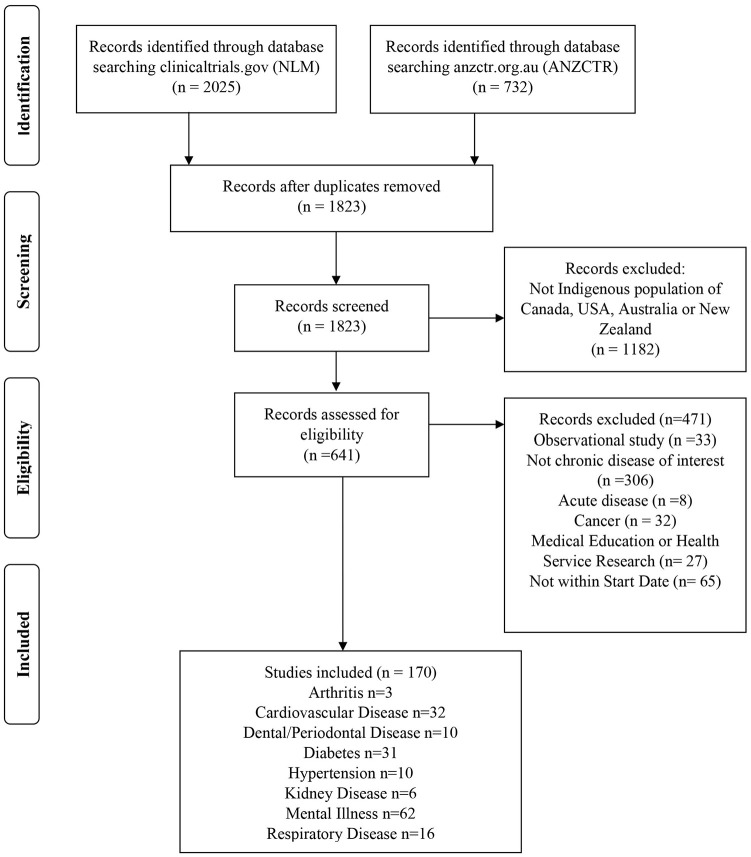
PRISMA flow diagram of studies included in environmental scan.

The Indigenous population–specific search retrieved a total of 2757 records from the two registries (2025 from NLM clinical trials.gov and 732 from ANZCTR). Following removal of duplicates, 1823 studies were screened. After excluding studies that did not meet the inclusion criteria, 170 studies were retained for analysis (Figure 1). Medical education and health service research studies were excluded because they focused on the training of health professionals working with Indigenous peoples and quality improvement studies for health programs and systems. A complete list of studies is summarized in Supplemental Table S1. The number of studies conducted in each country was 46 in Australia, 11 in Canada, 46 in New Zealand, and 67 in the United States; 1 New Zealand study was multi-national (Australia, Canada, and New Zealand); 110 studies enrolled only adults, and 60 studies included children <18 years. The chronic condition most addressed was mental illness (36%). The interventions were classified based on the categorization provided in the registry. The type of intervention across the four countries was predominantly focused on behavior change interventions (56%, n = 95) followed by a combination of behavioral, lifestyle, education, and rehabilitation interventions (15%, n = 25) and drug interventions (10%, n = 17) (Table 4). In all, 153 studies included participants of all genders, while 13 studies enrolled only females and 4 studies recruited only males. About 51% (n = 86) of the studies reported some level of community collaboration, while the other studies neither reported community involvement nor was it clearly stated. The primary government research agencies of the four countries, that is, the National Health & Medical Research Council (NHMRC), Canadian Institutes for Health Research (CIHR), Health Research Council (HRC), and National Institutes of Health (NIH) funded 87 studies. The other studies were funded by individuals, hospitals, universities, research institutes, industry, or other government agencies.

**Table 4.** Number of clinical trials in chronic diseases of interest by intervention types in Indigenous populations of Australia, Canada, New Zealand, and the United States.

Chronic condition	Intervention	Australia	Canada	New Zealand	United States
Arthritis	Combination^a^			1	
	Drug/biological			2	
Total = 3				3	
Cardiovascular disease	Behavioral	2		7	2
	Combination^a^	4		4	2
	Device			1	
	Drug/biological	2		1	1
	Other^b^	2			1
	Procedure/screening	2			1
Total = 32		***12***		***13***	***7***
Dental/periodontal disease	Behavioral	2		2	1
	Combination^a^	1			1
	Other^b^	2	1		
Total = 10		***5***	***1***	***2***	***2***
Diabetes	Behavioral	1	2	5	8
	Combination^a^	1		2	
	Device		2		
	Drug/biological	2		1	2
	Other^b^	1			1
	Procedure/screening	1	1	1	
Total = 31		***6***	***5***	***9***	***11***
Hypertension	Behavioral			1	7
	Device				1
	Other^b^		1		
Total = 10			***1***	***1***	***8***
Kidney disease	Behavioral		1		2
	Other^b^				2
	Procedure/screening	1			
Total = 6		***1***	***1***		***4***
Mental illness	Behavioral	9	3	8	29
	Combination^a^	1			2
	Device	1		1	
	Drug/biological	2			
	Other^b^	3		1	
	Procedure/screening	1		1	
Total = 62		***17***	***3***	***11***	***31***
Respiratory disease	Behavioral			1	2
	Combination^a^	1		3	2
	Drug/biological	2		2	
	Other^b^	1			
	Procedure/screening	1		1	
Total = 16		***5***		***7***	***4***

Bold italic values indicates the total number of clinical trial studies per chronic disease per country.

a Combination: a mix of Behavioral, Lifestyle, Education, and Rehabilitation interventions.

b Other: gardening, digital stories, lifestyle coaching, case management, Ma ka hana ka Ike, Hanapū Provider Toolbox.

### Published Indigenous-specific trials

Of the 170 trials we identified from the clinical trial registries, 48 were ongoing and did not provide final accrual data. Of the completed trials, 74 studies provided details of Indigenous enrolment within the registry enrolment data; for 47 of these 74 studies we obtained final accrual data from peer-reviewed publications, whereas 3 studies had no data on Indigenous enrollment listed and 45 studies did not provide final accrual data. Among the studies with either data in the registry or published results, a total of 16,635 Indigenous participants were enrolled (Australia =2827; Canada = 1495; New Zealand = 3507; United States = 8806), ranging from 1 participant up to 1451 participants (in a trial, n = 1 Indigenous participant published). The Indigenous peoples enrolled in the clinical trials were mainly American Indian and Alaska Native peoples (n = 7939), followed by Maori and Pacific Island peoples (n = 3178). Studies related to oral health enrolled the highest number of Indigenous participants (n = 4499). Also, Indigenous peoples were mostly enrolled into behavioral studies (n = 8951) compared with other intervention types. A total of 3905, 927, 1793, 888, 153, and 18 participants were enrolled in combined, drug, other, procedure, screening, and device intervention studies, respectively. Studies where community collaboration was mentioned were predominately in mental health (n = 38). The clinical trials were a combination of parallel, sequential, factorial, crossover, or single group allocations. In Australia, Canada, and New Zealand, the trials were a combination of randomized and nonrandomized designs, whereas in the US studies the design was predominantly randomized.

### Indigenous population enrolment in general population trials

The search of general population studies with results posted within the 10 years in Australia, Canada, New Zealand, and the United States in the chronic conditions of interest, identified 15,313 completed studies. After removing duplicates, 8302 studies were sorted based on the type of intervention (Supplemental Figure S1), 830 (10%) of the studies were randomly selected (Supplemental Table S2), and the proportion of Indigenous people enrolled in the studies extracted. Demographic characteristics of participants were not available for 26 studies. Although we attempted to contact the investigators connected with these studies to obtain demographic data, responses were not obtained. Indigenous peoples identified as Aboriginal, American Indian, or Alaska Native, Native Hawaiian or Pacific Islander, Aboriginal, Torres Strait Islander, Maori, or Indigenous. Of the remaining 804 studies, 278 (35%) studies did not provide race/ethnicity information to allow analysis. There were 526 studies (n = 208,941 participants) with race or ethnicity of participants reported, and Indigenous participants (n = 11,714) represented 5.6% of the total enrolled population. However, 354 (67%) did not include Indigenous participants at all. In studies with Indigenous participant enrolment, there was a median of 3 Indigenous participants per study (interquartile range (IQR: 1–6)), whereas the total enrolment for these same studies was a median of 225 (IQR: 98–612), of which 383 (48%) of the studies were funded by industry. Table 5 summarizes Indigenous population enrolment in chronic disease RCTs relative to general population enrolment.

**Table 5.** Summary of Indigenous population enrolment in general population chronic disease randomized controlled trials.

Location and total study enrolment	Population identity and enrolment	Chronic condition studied in the trial							
		Arthritis	CVD	Dental	Diabetes	HTN	Kidney	Mental illness	Respiratory
Australia (n = 49,692)	Indigenous, Aboriginal, and Torres Strait Islander (n = 7032, 14.2%)	–	14%	–	–	–	0.002%	0.1%	–
	General population (n = 42,660, 85.8%)	1.5%	65.9%		8.0%	0.2%	0.4%	9.6%	0.3%
Canada (n = 804)	First Nations, Inuit and Métis (n = 0, 0%)	–	–	–	–	–	–	–	–
	General population (n = 804, 100%)	7.5%	34.5%	23.9%	18.4%	2.0%		9.7%	4.0%
New Zealand (n = 654)	Maori and Pacific Islander (n = 96, 14.7%)	0.8%	1.7%	–	–	–	–	–	12.2%
	General population (n = 558, 85.3%)	15%	16.8%		33.3%			6.1%	14.1%
US (n = 187,391)	American Indian or Alaska Native, Native Hawaiian or Other Pacific Islander (n = 4180, 2.2%)	0.1%	1.4%	0.1%	0.4%	0.01%	0.01%	0.1%	0.2
	General population (n = 183,211, 97.8%)	4.3%	29.3%	2.6%	25.0%	6.8%	1.9%	13.4%	14.6%
Multiple countries (n = 120,385)	Indigenous (n = 406, 0.3%)	0.1%	0.02%	–	0.1%	–	0.01%	0.02%	0.1%
	General population (n = 119,979, 99.7%)	6.6%	40.3%	0.04%	32.6%	1.0%	2.0%	4.3%	12.8%

CVD: cardiovascular disease; HTN: hypertension.

Proportions rounded to 1 decimal place unless <0.05%.

## Discussion

Given the known diversity in treatment effects—both benefits and harms—it is important to include Indigenous populations in clinical trials. We have approached describing the current reality of Indigenous persons’ enrolment in clinical trials in two ways—the first to identify the frequency of Indigenous population–specific trials and their characteristics, and the second to identify how frequently Indigenous people are enrolled in general population clinical trials. As observed in this study, Indigenous population–specific clinical trials were frequently focused on behavior change.^22^ The behavioral interventions integrated and utilized several strategies, including education or group sessions offered in person or via mobile platforms. This type of intervention is critiqued for limitations in duration and sustainability of effect.^23^ Scale-up of interventions that are proven useful is a major problem in Indigenous research,^24^ and communities are burdened from contributing to research that is not scaled and sustained. Few Indigenous peoples were enrolled into drug-related clinical trials with limited enrolment in arthritis, hypertension, and kidney disease studies. This reduces the information about drug reactions regarding these conditions in this population, despite being among the most frequent conditions affecting Indigenous populations.^17,18^ Knowing that there are often differences in drug metabolism or response by race or ethnicity, it cannot be assumed that reactions to drugs are similar between different races.^25^ With the ongoing impact of colonialization on the health of Indigenous peoples, the increasing prevalence of chronic diseases in the population, and the upsurge of patient-focused medicine, representation of Indigenous peoples in clinical trials during the approval for new therapeutic drugs is vital in improving our understanding of how to prescribe the treatments and how the therapies function in the population.^26^ In addition, it is worth noting that certain chronic conditions are mostly studied in some Indigenous populations, which may be due to community, researchers, or government interest. For instance, although cardiovascular disease disproportionately affects Indigenous peoples in Canada compared with the general population,^27^ we were unable to identify Indigenous-specific clinical trials focused on this condition, rather there are more studies focused on other cardiometabolic conditions such as diabetes.

Indigenous peoples are a growing population in Australia, Canada, New Zealand, and the United States, with an estimated population of 7 million.^28^ Indigenous people account for about 3.3%, 4.9%, 15%, and 1.7% of the total Australian, Canadian, New Zealand, and the US population, respectively.^16,29^ The number of Indigenous people enrolled in clinical studies is below their overrepresentation in chronic disease prevalence.^30^ For example, it is estimated that 12.1% of American Indians and Alaska Native peoples above 18 years of age have coronary heart disease,^31^ but fewer than 1% are represented in clinical studies.^32^

Our result shows that among Indigenous population–specific chronic disease trials, a total of 16,635 participants were enrolled in the four countries, which is below population proportion. Although not every clinical trial needs to include all racial/ethnic groups, group(s) involved in studies must be representative of their larger population.^5^ This will provide meaningful opportunities to examine the multifaceted relationship between ancestral influences, environmental exposures, and social factors.^5^

Research ethics, particularly in regard to Indigenous research, focus on reducing health inequities, and research approaches framed around the cultural values of communal relationships and respect of world views.^33,34^ When working with Indigenous communities, clinical trials may not be plausible mainly due to ethical concerns arising from historical mistrust of researchers and the purposeful exclusion of some individuals from a beneficial intervention. Nevertheless, cultural appropriateness of the research should be encouraged and addressed.^1^ Clinical trial acceptance can be increased by design, particularly if traditional RCTs are not ethically applicable. Thus, methods that support access to the intervention to all participants, such as delayed interventions,^35^ should be considered. Our environmental scan provides examples of delayed intervention, and these approaches often coincide with cultural values of inclusion, which is essential for Indigenous community engagement.^36^ Although community engagement is often more reported in the North American literature due to differences in research funding policies and legislative framework differences regarding government–Indigenous community relations,^37^ this study showed that some level of community involvement that involved engaging Indigenous communities in the research processes was reported across all four countries. Prioritizing community engagement and education,^38^ and utilizing engaging strategies to recruit Indigenous peoples in clinical trials can assist in reducing barriers to enrollment.^39^

The principal government-funded health research bodies in Australia (NHMRC), Canada (CIHR), New Zealand (HRC), and the United States (NIH) have different policies regarding the inclusion of Indigenous peoples in research and the criteria to obtain research funding. In Australia, qualifying funding applications must address the NHMRC^40^ Indigenous Research Excellence criteria. Funded health research in New Zealand must address the attributes of the Prioritization Framework.^41^ In Canada, one of CIHR’s Institute of Indigenous Peoples’ Health strategic direction is to drive research, via increasing funding in Indigenous health research and providing funding directly to Indigenous communities.^42^ In the United States, to encourage including racial minorities in clinical trials, the NIH Revitalization Act passed the mandate that clinical researchers applying for NIH-funded research include women and people of diverse racial backgrounds in their studies based on analysis of the variables studied in the trial affect minority groups.^11^ The NIH also stipulated that clinical trials must include subgroup analyses to assess ethnic differences in treatment efficacy.^43^ In our study, most of the randomly selected general population trials either did not enroll Indigenous peoples or did not provide race and ethnicity information. About 56% of Indigenous population–specific chronic disease clinical trials were funded by the primary government funding agencies in the different counties. Nonetheless, sponsorship from government funding agencies could be improved to further meet their mandates and responsibilities of promoting Indigenous health and diversity in clinical research.^44,45^ It is essential that policies that support Indigenous people involvement and representation in clinical trials be created and put into practice. If the inclusion of Indigenous peoples is crucial, then better efforts need to be made, so they are adequately represented in clinical trials^39^ and ensure sufficiently powered subgroup analyses.^38^ In addition, integrating the Consolidated Standards of Reporting Trials (CONSORT)-Equity extension^46^ in clinical trials design may assist with creating awareness to consider equity when designing trials, guide in the revaluation of how to ensure engagement of subgroups, and advance analysis across population groups.

In Canada, the Truth and Reconciliation Commission of Canada^47^ calls on researchers to understand gaps in Indigenous health outcomes as a result of colonization. Research is essential to reconciliation and a vital aspect of closing the gaps in research, which often focuses on health conditions and outcomes. Yet, the type of research conducted equally matters. It is essential to increase the proportion of well-designed, high-quality Indigenous health clinical studies that address population health issues, supports reconciliation, and respects the right of Indigenous peoples to the highest attainable standard of health.^47^

## Limitations

We included only trials that were registered in the US NLM and the ANZCTR database. Accordingly, our results could have missed clinical trials registered in other databases, especially studies conducted across multiple locations. While this could be mitigated by conducting a systematic review of published studies as done by Saini and Quinn,^13^ we did not take this step. Replication of that study should be considered to provide updated results. Also, in the general population trial search, since we included studies designated in the registry as clinical trials/interventional studies, trials inappropriately registered as observational studies could have been missed.^48^ Since selection required being “completed recruitment” or “submitted results” and the other database was not verified, it is a limitation of our approach.^49^ We did not contact the authors of the published general population trials that did not report ethnicity to obtain this data, and our interpretations and conclusions resulting from the other studies that did are limited to the ability of participants to self-report their ethnicity.

## Conclusion

There is limited representation of Indigenous peoples in chronic disease clinical trials. This critique is not intended to impose on community which research topics or methods they choose to participate in, but rather highlights that large gaps exist when attempting to apply the evidence base to Indigenous peoples. Failure to create more racially diverse clinical research cohorts could increase existing health disparities if those most affected by disease continue to be excluded.^50^ Underrepresentation of Indigenous peoples in clinical trials decreases the opportunity to fully understand the factors that lead to poor health and outcomes of therapeutic interventions in the population.

## Supplemental Material

sj-pdf-1-ctj-10.1177_17407745211069153 – Supplemental material for The representation of Indigenous peoples in chronic disease clinical trials in Australia, Canada, New Zealand, and the United States Click here for additional data file. Supplemental material, sj-pdf-1-ctj-10.1177_17407745211069153 for The representation of Indigenous peoples in chronic disease clinical trials in Australia, Canada, New Zealand, and the United States by Valerie Umaefulam, Tessa Kleissen and Cheryl Barnabe in Clinical Trials

## References

[1] Glover M Kira A Johnston V et al. A systematic review of barriers and facilitators to participation in randomized controlled trials by Indigenous people from New Zealand, Australia, Canada and the United States. Glob Health Promot 2015; 22(1): 21–31. 24842989 10.1177/1757975914528961

[2] Nazha B Mishra M Pentz R et al. Enrollment of racial minorities in clinical trials: old problem assumes new urgency in the age of immunotherapy. Am Soc Clin Oncol Educ Book 2019; 39: 3–10. 31099618 10.1200/EDBK_100021

[3] Hurd K Barnabe C. Mortality causes and outcomes in Indigenous populations of Canada, the United States, and Australia with rheumatic disease: a systematic review. Semin Arthritis Rheum 2018; 47(4): 586–592. 28823732 10.1016/j.semarthrit.2017.07.009

[4] Hernandez LM Blazer DG. Genes, behavior, and the social environment: moving beyond the nature/nurture debate. Washington, DC: National Academies Press, 2006, https://www.ncbi.nlm.nih.gov/books/NBK19932/20669442

[5] Oh SS Galanter J Thakur N et al. Diversity in clinical and biomedical research: a promise yet to be fulfilled. PLoS Med 2015; 12(12): e1001918. 26671224 10.1371/journal.pmed.1001918 PMC4679830

[6] Nagaraj S Barnabe C Schieir O et al. Early rheumatoid arthritis presentation, treatment, and outcomes in aboriginal patients in Canada: a Canadian early arthritis cohort study analysis. Arthritis Care Res 2018; 70(8): 1245–1250. 10.1002/acr.23470 29125904

[7] Canuto K McDermott R Cargo M. Participant views on participating in a pragmatic randomised controlled trial: the Aboriginal and Torres Strait Islander Women’s Fitness Program. Int J Equity Health 2014; 13(1): 77. 25192793 10.1186/s12939-014-0077-3 PMC4172823

[8] Hamel LM Penner LA Albrecht TL et al. Barriers to clinical trial enrollment in racial and ethnic minority patients with cancer. Cancer Control 2016; 23(4): 327–337. 27842322 10.1177/107327481602300404 PMC5131730

[9] Maar MA Beaudin V Yeates K et al. Wise practices for cultural safety in electronic health research and clinical trials with Indigenous people: secondary analysis of a randomized clinical trial. J Med Internet Res 2019; 21(11): e14203. 31682574 10.2196/14203 PMC6862000

[10] Redwood S Gill PS. Under-representation of minority ethnic groups in research-call for action. Br J Gen Pract 2013; 63(612): 342–343. 23834862 10.3399/bjgp13X668456 PMC3693777

[11] Zhang T Tsang W Wijeysundera HC et al. Reporting and representation of ethnic minorities in cardiovascular trials: a systematic review. Am Heart J 2013; 166(1): 52–57. 23816021 10.1016/j.ahj.2013.03.022

[12] Morris PS. Randomised controlled trials addressing Australian aboriginal health needs: a systematic review of the literature. J Paediatr Child Health 1999; 35(2): 130–135. 10365347

[13] Saini M Quinn A. A systematic review of randomized controlled trials of health related issues within and Aboriginal context, 2013, https://www.nccih.ca/docs/context/RPT-ReviewRCTs-Saini-Quinn-EN.pdf

[14] Hunter KE Xu G Modi D et al. The landscape of clinical trial activity focusing on Indigenous health in Australia from 2008 to 2018, 2019, https://colloquium2019.cochrane.org/abstracts/landscape-clinical-trial-activity-focusing-indigenous-health-australia-2008-2018 10.1186/s12889-022-13338-y PMC9107126 35568933

[15] Graham P Evitts T Thomas-MacLean R. Environmental scans: how useful are they for primary care research? Can Fam Physician 2008; 54(7): 1022–1023. 18625830 PMC2464800

[16] Berger DN Bulanin N García-Alix L et al. The Indigenous World 2020, 34th ed, 2020, http://iwgia.org/images/yearbook/2020/IWGIA_The_Indigenous_World_2020.pdf

[17] Australian Institute of Health and Welfare. The health and welfare of Australia’s Aboriginal and Torres Strait Islander peoples: 2015. Canberra, ACT, Australia: Australian Institute of Health and Welfare, 2015.

[18] Jones M Weaver S Panahi S et al. Indigenous peoples health in the United States of America: review of lifestyle issues and the implementation of community-based participatory research. Divers Equal Heal Care 2018; 15(2): 66–70.

[19] Pizzoli SFM Renzi C Arnaboldi P et al. From life-threatening to chronic disease: is this the case of cancers? A systematic review. Cogent Psychol 2019; 6(1): 1577593.

[20] Bernell S Howard SW. Use your words carefully: what is a chronic disease? Front Public Health 2016; 4: 159–154. 10.3389/fpubh.2016.00159 PMC4969287 27532034

[21] Chauhan BF Jeyaraman M Mann AS et al. Behavior change interventions and policies influencing primary healthcare professionals’ practice-an overview of reviews. Implement Sci 2017; 12(1): 3. 28057024 10.1186/s13012-016-0538-8 PMC5216570

[22] Rice K Te Hiwi B Zwarenstein M et al. Best practices for the prevention and management of diabetes and obesity-related chronic disease among Indigenous peoples in Canada: a review. Can J Diabetes 2016; 40(3): 216–225. 27066857 10.1016/j.jcjd.2015.10.007

[23] Ory MG Lee Smith M Mier N et al. The science of sustaining health behavior change: the health maintenance consortium. Am J Health Behav 2010; 34(6): 647–659. 20604691 10.5993/ajhb.34.6.2 PMC3753403

[24] McCalman J Bainbridge R Percival N et al. The effectiveness of implementation in Indigenous Australian healthcare: an overview of literature reviews. Int J Equity Health 2016; 15: 47. 26965040 10.1186/s12939-016-0337-5 PMC4787175

[25] Bonham VL Callier SL Royal CD. Will precision medicine move us beyond race? N Engl J Med 2016; 374(21): 2003–2005. 27223144 10.1056/NEJMp1511294 PMC5621043

[26] Hoppe C Kerr D. Minority underrepresentation in cardiovascular outcome trials for type 2 diabetes. Lancet Diabetes Endocrinol 2017; 5(1): 13. 28010783 10.1016/S2213-8587(16)30324-2

[27] Foulds HJA Bredin SSD Warburton DER . Cardiovascular dynamics of Canadian Indigenous peoples. Int J Circumpolar Health 2018; 77(1): 1421351. 29405888 10.1080/22423982.2017.1421351 PMC5804726

[28] Pulver LJ Haswell MR Ring I et al. Indigenous health—Australia, Canada, Aotearoa New Zealand and the United States—laying claim to a future that embraces health for us all, 2010, http://www.who.int/healthsystems/topics/financing/healthreport/IHNo33.pdf

[29] U.S. Census Bureau. Census 2010 American Indian and Alaska native summary file. Washington, DC: U.S. Census Bureau, 2010.

[30] Gionet L Roshanafshar S. Select health indicators of First Nations people living off reserve, Metis and Inuit, 2013, http://www.statcan.gc.ca/pub/82-624-x/2013001/article/11763-eng.pdf

[31] Benjamin EJ Muntner P Alonso A et al. Heart disease and stroke statistics-2019 update: a report from the American Heart Association. Circulation 2019; 139: e56–e528. 30700139 10.1161/CIR.0000000000000659

[32] Vigil D Sinaii N Karp B. American Indian and Alaska native enrollment in clinical studies in the National Institutes of Health’s intramural research program. Ethics Hum Res 2021; 43(3): 2–9. 10.1002/eahr.500090 34019337

[33] Health Research Council of New Zealand. Pacific health research guidelines, 2014, www.hrc.govt.nz

[34] Canadian Institutes of Health Research, Natural Sciences and Engineering Research Council of Canada and Social Sciences and Humanities Research Council of Canada. Tri-council policy statement: ethical conduct for research involving humans, 2018, https://www.cmcc.ca/Tri-Council%20Policy%20Statement.pdf

[35] Spineli LM Jenz E Großhennig A et al. Critical appraisal of arguments for the delayed-start design proposed as alternative to the parallel-group randomized clinical trial design in the field of rare disease. Orphanet J Rare Dis 2017; 12(1): 140. 28814322 10.1186/s13023-017-0692-3 PMC5559817

[36] Henry D Tolan P Gorman-Smith D et al. Alternatives to randomized control trial designs for community-based prevention evaluation. Prev Sci 2017; 18(6): 671–680. 27600286 10.1007/s11121-016-0706-8

[37] Lin CY Loyola-Sanchez A Hurd K et al. Characterization of Indigenous community engagement in arthritis studies conducted in Canada, United States of America, Australia and New Zealand. Semin Arthritis Rheum 2019; 49(1): 145–155. 30598333 10.1016/j.semarthrit.2018.11.009

[38] Falasinnu T Chaichian Y Simard JF. Increasing ancestral diversity in lupus trials: ways forward. Rheum Dis Clin North Am 2020; 46(4): 713–722. 32981648 10.1016/j.rdc.2020.07.011 PMC11075620

[39] Falasinnu T Chaichian Y Bass MB et al. The representation of gender and race/ethnic groups in randomized clinical trials of individuals with systemic lupus erythematosus. Curr Rheumatol Rep 2018; 20(4): 20. 29550947 10.1007/s11926-018-0728-2 PMC5857270

[40] NHMRC. Funding rules involving Aboriginal and Torres Strait Islander people, 2020, https://www.nhmrc.gov.au/health-advice/aboriginal-and-torres-strait-islander-health/funding-rules-involving-aboriginal-and-torres-strait-islander-people

[41] Health Research Council of New Zealand. The New Zealand health research prioritisation framework, 2019, https://www.hrc.govt.nz/resources/new-zealand-health-research-prioritisation-framework

[42] Canadian Institutes of Health Research. Institute of Indigenous peoples’ health: strategic plan 2019-2024, 2019, https://cihr-irsc.gc.ca/e/8668.html

[43] National Institutes of Health. National Institutes of Health Revitalization Act of 1993, 1993, https://www.congress.gov/103/statute/STATUTE-107/STATUTE-107-Pg122.pdf

[44] NHMRC. Road map II: a strategic framework for improving the health of Aboriginal and Torres Strait Islander people through research, 2010, https://www.nhmrc.gov.au/about-us/publications/road-map-ii-strategic-framework-improving-health-aboriginal-and-torres-strait-islander-people-through-research

[45] Health Research Council of New Zealand. Guidelines for researchers on health research involving Māori, 2010, http://www.hrc.govt.nz

[46] Welch VA Norheim OF Jull J et al. CONSORT-Equity 2017 extension and elaboration for better reporting of health equity in randomised trials. BMJ 2017; 359: j5085. 29170161 10.1136/bmj.j5085

[47] Truth and Reconciliation Commission of Canada. Calls to action, 2015, http://trc.ca/assets/pdf/Calls_to_Action_English2.pdf

[48] Nicholls SG Carroll K Hey SP et al. A review of pragmatic trials found a high degree of diversity in design and scope, deficiencies in reporting and trial registry data, and poor indexing. J Clin Epidemiol 2021; 137: 45–57. 33789151 10.1016/j.jclinepi.2021.03.021 PMC8996736

[49] Fleminger J Goldacre B. Prevalence of clinical trial status discrepancies: a cross-sectional study of 10, 492 trials registered on both ClinicalTrials.gov and the European Union Clinical Trials Register. PLoS One 2018; 13(3): e0193088. 29513684 10.1371/journal.pone.0193088 PMC5841737

[50] Konkel L. Racial and ethnic disparities in research studies: the challenge of creating more diverse cohorts. Environ Health Perspect 2015; 123(12): A297–A302. 26625444 10.1289/ehp.123-A297 PMC4670264

