# Supplementary material for: The representation of Indigenous peoples in chronic disease clinical trials in Australia, Canada, New Zealand, and the United States
Source: Clin Trials. 2022 Jan 6;19(1):22–32. doi: 10.1177/17407745211069153 (PMC8847750; doi:10.1177/17407745211069153)

Supplementary Tables and Figures Legend

Table S1. Summary of Indigenous specific studies. 2

Table S2. Summary of randomly selected studies. 19

Figure S1. PRISMA flow diagram of general population trials. 77

Table S1. Summary of Indigenous specific studies included in environmental scan.

Title	Start Date	Location	Indigenous Identity	Conditions	Interventions	Gender	Age	Actual Indigenous Enrollment	Study Designs	Community Partner	Funded By
Associations between periodontal disease and cardiovascular surrogate endpoints in an adult Indigenous population	2010	Australia	Indigenous	Dental Disease	Other	All	25 Years and older	269	Randomized, Delayed intervention design	Not stated	NHMRC
Getting better at chronic care in North Queensland: A cluster randomized trial of patient-centred care delivered by Indigenous health professionals to Indigenous clients.	2010	Australia	Indigenous	Cardiovascular Disease	Combination	All	18 to 65 Years	No data obtained	Randomized, Parallel Assignment	Yes	NHMRC
Reducing disease burden and health inequalities arising from chronic dental disease among Indigenous children: an early childhood caries intervention	2011	Australia	Aboriginal	Dental Disease	Behavioral	All	No age limit	446	Randomized, Parallel Assignment	Yes	NHMRC
Tasmanian Aborigines Step Up to Health: evaluation of a cardiopulmonary rehabilitation and secondary prevention program	2011	Australia	Aborigine	Cardiovascular Disease	Combination	All	18 Years and older	92	Non-randomized, Single Group	Yes	Other
The Central Australian Heart Protection Study: A Randomised Trial of Nurse-Led, Family Based Secondary Prevention of Acute Coronary Syndromes.	2011	Australia	Indigenous	Cardiovascular Disease	Combination	All	18 Years and older	No data obtained	Randomized, Parallel Assignment	Not stated	NHMRC
"Smiles not Tears" an Aboriginal Health Worker Led Dental Health Education Program.	2012	Australia	Aboriginal	Dental Disease	Combination	All	5 to7 months	187	Non-Randomized, Single Group	Yes	NHMRC
Pilot of a self-help app to support suicide prevention amongst Indigenous youth	2013	Australia	Indigenous	Mental illness	Behavioral	All	18 to 25 Years	61	Randomized	Yes	Other
The evaluation of telehealth technologies to facilitate clinical outcomes improvements in eye care, diabetes and cardiovascular disease management for Indigenous Australians.	2013	Australia	Indigenous Australians	Diabetes	Other	All	18 Years and older	Not completed	Non-randomized	Yes	NHMRC

Using a patient-focussed electronic health system for reducing heart disease risk in people with cardiovascular disease.	2013	Australia	Aboriginal	Cardiovascular Disease	Behavioral	All	18 Years and older	No published results	Randomized, Parallel Assignment	Not stated	NHMRC
Using Pneumococcal Vaccines in Combination for Maximum Protection From Ear and Lung Infections in First 3 Years of Life	2013	Australia	Australian Indigenous	Respiratory disease	Biological	All	9 Months to 3 Years	261	Randomized, Parallel Assignment	Yes	NHMRC
Far North Queensland Hospital Avoidance Trial - chronic disease case management compared with usual care in reducing avoidable hospital and Emergency Department admissions.	2014	Australia	Aboriginal or Torres Strait Islander	Cardiovascular Disease	Other	All	18 to75 Years	No data obtained	Randomized	Not stated	Other
Optimum Thiamine Intervention (OpTIn) Trial	2014	Australia	Aboriginal and non-Aboriginal patients	Mental illness	Drug	All	18 to 65 Years	No published results	Randomized, Parallel Assignment	Not stated	NHMRC
Optimum Thiamine Intervention for Treatment and Prevention of Wernicke-Korsakoff Syndrome (WKS): A Randomised Controlled Trial.	2014	Australia	Aboriginal	Mental illness	Drug	All	18 to 65 Years	No published results	Randomized, Parallel Assignment	Not stated	NHMRC
The effect of a culturally and linguistically appropriate patient education tool to improve conclusive electrocardiography stress testing results in Aboriginal and non-Aboriginal Australian patients.	2014	Australia	Aboriginal	Cardiovascular Disease	Behavioral	All	18 Years and older	No published results	Randomized, Parallel Assignment	Not stated	Other
Using an app for suicide prevention amongst young Indigenous people: A randomised controlled trial	2014	Australia	Indigenous	Mental illness	Behavioral	All	16 Years and older	350	Randomized	Not stated	NHMRC
Preventing tooth decay in children in a remote community in Australia.	2015	Australia	Indigenous	Dental Disease	Other	All	4 to 17 Years	435	Non-randomized, Single Group	Yes	NHMRC
The Aboriginal Cardiovascular Omega-3 Randomised Controlled Trial	2015	Australia	Indigenous Australian	Cardiovascular Disease	Drug	All	18 Years and older	89	Randomized, Parallel Assignment	Not stated	NHMRC
The effect of a periodontal intervention on renal health	2015	Australia	Aboriginal	Kidney Disease	Procedure	All	18 Years	600	Randomized, Parallel Assignment	Yes	NHMRC

in Aboriginal Australian adults with kidney disease							and older				
Evaluating the efficacy and adherence of administration of a PCSK9 inhibitor Alirocumab in Aboriginal participants with hypercholesterolaemia	2016	Australia	Aboriginal	Cardiovascular Disease	Drug	All	18 Years and older	No published results	Randomized, Parallel Assignment	Not stated	Other
Exenatide -LAR in addition to standard care in Indigenous Australians with type 2 diabetes and effect on glycaemic control	2016	Australia	Indigenous	Diabetes	Drug	All	18 Years and older	37	Randomized	Not stated	Other
Hospitalised Pneumonia With Extended Treatment (HOPE) Study	2016	Australia	Indigenous	Respiratory disease	Drug	All	3 Months to 5 Years	Not completed	Randomized, Parallel Assignment	Not stated	Other
Is opportunistic screening appropriate for identifying arrhythmias and establishing effective clinical pathways for Aboriginal Australians?	2016	Australia	Aboriginal and Torres Strait Islander peoples	Cardiovascular Disease	Screening	All	45 Years and older	No published results	Non-randomized, Single group	Yes	Other
Aboriginal and non-Aboriginal women perpetrators of violence: a trial of a prison-based intervention (Beyond Violence)	2017	Australia	Aboriginal	Mental illness	Behavioral	Female	18 Years and older	No published results	Non-randomized	Not stated	NHMRC
Comparing the effectiveness and safety of two treatments (OZURDEX® intravitreal implant versus Avastin®) for diabetic eye disease in adult Indigenous patients living in Western Australia.	2017	Australia	Indigenous	Diabetes	Drug	All	18 Years and older	No published results	Randomized, Parallel Assignment	Not stated	Other
Evaluation of a community-based assertive follow-up suicide prevention program for people who have attempted suicide or are at high risk of suicide	2017	Australia	Indigenous	Mental illness	Other	All	16 Years and older	No published results	Non-randomized	Yes	Other
Managing chronic lung disease in Aboriginal communities: the Breathe Easy Walk Easy-Lungs for Life (BE WELL) project	2017	Australia	Aboriginal	Respiratory disease	Combination	All	40 to 90 Years	Not completed	Non-randomized, Single group	Yes	NHMRC
The effectiveness and acceptance of eye movement desensitisation and reprocessing (EMDR) from a	2017	Australia	Aboriginal and Torres Strait Islander	Mental illness	Other	All	18 to 70 Years	No data obtained	Non-randomized, Single Group	Yes	HRC

remote Aboriginal Australian community perspective.											
Wellbeing Intervention for Chronic Kidney Disease (WICKD): A Trial of the Aboriginal and Islander Mental Health Initiative (AIMhi) Stay Strong App.	2017	Australia	Aboriginal or Torres Strait Islander.	Mental illness	Device	All	18 Years and older	No published results	Randomized	Yes	NHMRC
A model of culturally-informed integration of diabetic retinopathy screening and diabetes education assessment in Indigenous primary care settings.	2018	Australia	Indigenous	Diabetes	Screening	All	18 Years and older	No published results	Non-randomized, Single Group	Yes	Other
A pilot trial of a smartphone application to support the mental health of young apprentices	2018	Australia	Aboriginal and Torres Strait	Mental illness	Behavioral	All	16 to 30 Years	No published results	Non-Randomized, Single Group	Not stated	Other
A randomised controlled trial to determine if use of a Bronchiectasis Action Management Plans (BAMP) compared to usual care improves clinical outcomes in children with bronchiectasis.	2018	Australia	Aboriginal and/or Torres Strait Islander	Respiratory Disease	Other	All	18 Years and older	Not completed	Randomized, Parallel Assignment	Not stated	NHMRC
Adapting Compassion Focused Therapy for Groups with Aboriginal and Torres Strait Islander Clients	2018	Australia	Aboriginal and Torres Strait Islander	Mental illness	Behavioral	All	19 Years and older	No published results	Non-Randomized, Single Group	Yes	Other
Community Intervention to Reduce Tobacco Use in Pregnant Women	2018	Australia	Aboriginal	Mental illness	Combination	Female	16 Years and older	No published results	Non-randomized, Single Group	Yes	Other
Electronic risky health behaviour and mental health screening for New Zealand youth aged 12 - 24 years in primary health care.	2018	Australia	Maori	Mental illness	Screening	All	16 to 65 Years	Not completed	Randomized, Parallel Assignment	Yes	NHMRC
Integrating Pharmacists within Aboriginal Community Controlled Health Services to improve Chronic Disease Management Project	2018	Australia	Aboriginal	Cardiovascular Disease	Other	All	18 Years and older	No published results	Non-randomized	Yes	Other
Management of dental decay in young Aboriginal children	2018	Australia	Aboriginal	Dental Disease	Behavioral	All	72 months and younger	Not completed	Randomized, Parallel Assignment	Yes	NHMRC

Yeddung Gauar (Good Heart): Feasibility of an Aboriginal and Torres Strait Islander female Cardiac Rehabilitation program delivered in a non-Indigenous health service.	2018	Australia	Indigenous	Cardiovascular Disease	Combination	Female	18 Years and older	No published results	Non-randomized, Single Group	Yes	Other
Improving self management of blood sugar control in people with Type 2 Diabetes.	2019	Australia	Indigenous	Diabetes	Combination	All	18 Years and older	Not completed	Randomized, Parallel Assignment	Not stated	Other
Indigenous Model of Mental Health Care	2019	Australia	Indigenous	Mental illness	Other	All	18 Years and older	No published results	Randomized, Parallel Assignment	Yes	NHMRC
Prevention of Diabetes Progression in Primary Care through Shared Medical Appointments	2019	Australia	Aboriginal	Diabetes	Behavioral	All	18 to 65 Years	Not completed	Randomized, Parallel Assignment	Not stated	NHMRC
We Can Do This: Web-based therapeutic intervention for reducing methamphetamine use amongst Aboriginal and Torres Strait Islander people: Randomised wait-list control Trial	2019	Australia	Aboriginal and Torres Strait Islander	Mental illness	Behavioral	All	16 Years and older	Not completed	Randomized, Parallel Assignment	Yes	NHMRC
Community screening of participants across community pharmacies detecting asymptomatic clinical markers or risk factors associated with cardiovascular disease and chronic disease.	2019	Australia	Aboriginal or Torres strait Islander	Cardiovascular Disease	Screening	All	35-74 Years	Not completed	Randomized	Not stated	Other
Standing Tall with Our Mob Program (STOMP) pilot trial to improve mobility, balance, physical activity, cognitive function and psychological well-being with older people in an urban Aboriginal community.	2019	Australia	Aboriginal or Torres strait Islander	Mental illness	Behavioral	All	45 Years and older	No published results	Randomized, Parallel Assignment	Not stated	NHMRC
Yawardani Jan-ga (Horses helping healing in Yawuru language): Using equine-assisted learning to improve the social and emotion wellbeing of Aboriginal young people and prevent suicide	2019	Australia	Aboriginal	Mental illness	Behavioral	All	6-25 Years	Not completed	Non-randomized, Single group assignment	Not stated	Other

A Pilot Study using Non-Contact Smartphone Cough Sound Recordings to Screen for Chronic Obstructive Pulmonary Disease in an Indigenous Australian Adult Population	2020	Australia	Indigenous	Respiratory Disease	Screening	All	22 Years and older	Not completed	Non-Randomized	Not stated	Other
Talking about Aboriginal Gambling: Examining the effectiveness of Facebook groups to reduce gambling harm within Aboriginal communities in New South Wales, Australia	2020	Australia	Aboriginal	Mental illness	Behavioral	All	18 Years and older	Not completed	Randomized	Yes	Other
The Aboriginal Youth Mentorship Program	2010	Canada	Aboriginal	Diabetes	Behavioral	All	8 to 11 Years	246	Non-Randomized, Crossover Assignment	Yes	Other
Circle of Life. Improving Capacity of Swampy Cree Community Members to Recognize People at Risk for Suicide	2011	Canada	First Nations	Mental illness	Behavioral	All	16 Years and older	55	Randomized, Parallel Assignment| Primary Purpose: Prevention	Yes	CIHR
Point of Care HbA1c as a Screening Test for Type 2 Diabetes in First Nations	2011	Canada	First Nations	Diabetes	Procedure	All	14 Years and older	258	Single Group Assignment	Yes	CIHR
Preventing Early Childhood Caries in Indigenous Children: the Baby Teeth Talk Study	2011	Canada	Indigenous	Dental disease	Other	Female	Child, Adult, Older Adult	544	Randomized, Parallel Assignment	Yes	CIHR
Detecting and Treating High Blood Pressure in Aboriginal Population and Low and Middle Income Countries	2012	Canada	Indigenous Canadian and Rural Tanzanian communities	Hypertension	Other	All	18 to 90 Years	243	Randomized, Parallel Assignment	Not stated	CIHR
Healthy Moms, Healthy Babies	2012	Canada	First Nations	Diabetes	Device	Female	18 Years and older	18	Non-Randomized, Parallel Assignment	Not stated	Other
The Cedar Project: Impact of mHealth for HIV Prevention Among Young Indigenous People Who Use Illicit Drugs	2014	Canada	Indigenous	Mental illness	Behavioral	All	19 Years and older	131	Randomized, Parallel Assignment	Yes	CIHR
The Role and Effectiveness of Diabetes Coaches in British Columbia	2014	Canada	Aboriginal	Diabetes	Behavioral	All	19 to 80 Years	No data on Indigenous enrollment	Single Group Assignment	Yes	Other
Diabetes Foot Care Clinical Pathway - Orpyx Medical Technologies	2016	Canada	First Nations	Diabetes	Device	All	18 Years	No published results	Randomized, Parallel Assignment	Not stated	Other

							and older				
A Randomized Controlled Trial of a Pre-Transplant Education Intervention	2018	Canada	First Nations	Kidney Disease	Behavioral	All	0 to18 Years	Not completed	Randomized, Parallel Assignment	Yes	Other
Smartphone-enabled Health Coaching Intervention for Youth Diagnosed With Major Depressive Disorders	2018	Canada	First Nations	Mental illness	Behavioral	All	18 to 30 Years	Not completed	Randomized, Parallel Assignment	Not stated	Other
Improving Adherence using Combination Therapy	2010	New Zealand	Maori	Cardiovascular Disease	Drug	All	18 to 80 Years	257	Randomized, Parallel Assignment	Not stated	HRC
Heart Exercise And Remote Technologies Trial	2011	New Zealand	Maori	Cardiovascular Disease	Combination	All	18 Years and older	No data obtained	Randomized, Parallel Assignment	Not stated	HRC
Prospective randomised controlled trial comparing the efficacy of laparoscopic Roux-en-Y gastric bypass versus laparoscopic sleeve gastrectomy for the management of type 2 diabetes mellitus in obese patients	2011	New Zealand	Maori and Pacific Island	Diabetes	Procedure	All	20 to 50 Years	30	Randomized, Parallel Assignment	Not stated	Other
The effect of an oral health education program for mothers and fluoride treatment on oral health in Indigenous Maori children	2011	New Zealand	Maori	Dental Disease	Behavioral	Female	16 Years and older	222	Randomized, Parallel Assignment	Yes	HRC
The NATINATI Study: A single intervention to compare the effects of hazelnut consumption on the acceptance and cardiovascular disease risk factors among Maori and European participants.	2011	New Zealand	Maori and European	Cardiovascular Disease	Behavioral	All	18 to 40 Years	20	Non-randomized, Single Group	Not stated	Other
The Nuts2 Study: The effect of daily consumption of raw versus roasted/salted hazelnuts on Cardiovascular disease risk factors and acceptability in Maori and European populations	2011	New Zealand	Maori and European	Cardiovascular Disease	Behavioral	All	18 to 65 Years	No data on Indigenous enrollment	Randomized, Crossover Assignment	Not stated	Other
Antibiotics for bronchiectasis exacerbations in children - The Bronchiectasis Exacerbation Study (Study 1).	2012	New Zealand	Indigenous	Respiratory Disease	Drug	All	19 years and below	88	Randomized, Parallel Assignment	Not stated	NHMRC

Antibiotics for treatment of bronchiectasis exacerbations in children - The Bronchiectasis Exacerbation Study (Study 2).	2012	New Zealand	Indigenous	Respiratory Disease	Drug	All	19 years and below	70	Randomized, Parallel Assignment	Not stated	NHMRC
Safety and efficacy of high dose allopurinol in the management of gout: a randomised interventional study	2012	New Zealand	Maori and Pacific Island	Arthritis	Drug	All	18 Years and older	77	Randomized, Parallel Assignment	Not stated	HRC
Safety and efficacy of immediate versus delayed allopurinol treatment for the prevention of gout flares in pacific, maori and other people	2012	New Zealand	Maori and Pacific people	Arthritis	Drug	All	18 to 75 Years	20	Randomized, Parallel Assignment	Not stated	Other
The effect of a 12-week exercise and lifestyle management programme on cardiac risk reduction: A pilot using a kaupapa Maori philosophy	2012	New Zealand	Maori	Cardiovascular Disease	Behavioral	All	18 Years and older	9	Non-randomized	Not stated	Other
YourCall Study: The effectiveness of text messaging to address hazardous drinking among trauma patients	2012	New Zealand	Maori and Pacific	Mental illness	Behavioral	All	16 to 69 Years	202	Randomized	Yes	HRC
Non-randomised pilot study of a text message self-management support programme for people with diabetes	2013	New Zealand	Maori	Diabetes	Behavioral	All	16 to 65 Years	18	Non-randomized, Single Group	Not stated	Other
Strengthening health literacy among Indigenous people living with cardiovascular disease, their families, and health care providers	2013	Multiple	Indigenous	Cardiovascular Disease	Behavioral	All	20 Years and older	171	Non-randomized, Single group	Yes	HRC, NHMRC, CIHR
Do Defects in the Innate Immune System Contribute to Non-Cystic Fibrosis Bronchiectasis in Maori and Pacific Island Children?	2014	New Zealand	Maori and Pacific Island	Respiratory Disease	Screening	All	0 to15 years	49	Non-randomized, Single Group allocation	Not stated	Other
A supervised toothbrushing intervention trial in Northland New Zealand school children	2015	New Zealand	Maori	Dental Disease	Behavioral	All	10 to 13 Years	222	Non-randomized, Quasi-experimental	Not stated	Other
Diabetes text message self management support	2015	New Zealand	Maori and Pacific	Diabetes	Behavioral	All	16 Years and older	72	Randomized, Parallel Assignment	Not stated	HRC

Effectiveness of problem gambling interventions in a service setting: A pragmatic randomised clinical trial	2015	New Zealand	Maori	Mental illness	Behavioral	All	18 Years and older	No published results	Randomized, Parallel Assignment	Yes	Other
Efficacy of a kaupapa Maori approach, within an exercise and lifestyle programme, on cardiovascular disease risk	2015	New Zealand	Maori	Cardiovascular Disease	Behavioral	All	18 Years and older	No published results	Non-randomized, Single group	Yes	Other
Implanted (brain) Stimulators to Augment stroke Rehabilitation Therapy (iSTART trial)	2016	New Zealand	Maori and Pacific Island	Cardiovascular Disease	Device	All	18 Years and older	No published results	Randomized	Not stated	Other
Ka Mau Te Wehi: Culturally appropriate weight Loss intervention for Maori and Pacific people of New Zealand	2016	New Zealand	Maori and Pacific people	Cardiovascular Disease	Combination	All	16 Years and older	165	Non-randomized, Parallel Assignment	Not stated	Other
The effect of aerobic exercise training on cardiovascular function in aged adults with and without type 2 diabetes	2016	New Zealand	Maori and Pacific Island	Diabetes	Behavioral	All	35 to 80 Years	Not completed	Non-randomized, Parallel Assignment	Not stated	HRC
Acceptability and utility of electronic screener, YouthCHAT (Youth Version Case-finding Help Assessment Tool), and its comparison with the Home, Education, Eating, Activities, Drugs and Alcohol, Sexuality, Suicide/Depression, Safety (HEEADSSS) assessment. for young people with long-term physical conditions and high school students.	2016	New Zealand	Maori and Pacific Island	Mental illness	Screening	All	13-18 Years	104	Randomized, Crossover Assignment	Not stated	Other
Efficacy of a health and wellness coaching program for the prevention of cardiovascular disease and stroke in New Zealand adults	2016	New Zealand	Maori and Pasifika	Cardiovascular Disease	Behavioral	All	35 Years and older	160	Randomized, Parallel Assignment	Not stated	Other
BetaMe. An innovative management of diabetes and prediabetes with a comprehensive digital health programme: a randomised controlled trial.	2017	New Zealand	Maori and Pacific	Diabetes	Combination	All	18 to 75 Years	No published results	Randomized, Parallel Assignment	Yes	HRC
Can a 'take charge' intervention reduce incidence of repeat acute exacerbation of chronic obstructive	2017	New Zealand	Maori and Pacific Island	Respiratory disease	Combination	All	18 Years and older	No published results	Randomized, Parallel Assignment	Not stated	HRC

pulmonary disease? A feasibility study											
Can better sleep health during pregnancy regulate depressive symptoms in women with a previous history of depression?	2017	New Zealand	Maori	Mental illness	Behavioral	Female	16 Years and older	22	Non-Randomized, Single Group	Yes	Other
Community exercise and education to improve blood glucose control, physical health outcomes, and well-being for people living with type II diabetes.	2017	New Zealand	Maori and Pacific	Diabetes	Combination	All	35 Years and older	No published results	Randomized, Parallel Assignment	Not stated	HRC
Fibre structure of whole grains in blood glucose response	2017	New Zealand	Maori and Pacific Island	Diabetes	Behavioral	All	18 to 75 Years	30	Randomized, Crossover Assignment	Not stated	Other
He Kura: Improving Asthma Support for School Children	2017	New Zealand	Maori	Respiratory disease	Behavioral	All	5 to 13 Years	No published results	Non-randomized, Single group	Yes	HRC
Walking to better health after stroke	2017	New Zealand	Maori	Cardiovascular Disease	Combination	All	18 Years and older	1	Randomized, Parallel Assignment	Not stated	Other
Whakapai e Te Ara Ha: Asthma Self-Management Programme for the whanau of tamariki Maori with Asthma	2017	New Zealand	Maori	Respiratory disease	Combination	All	All Years	No published results	Randomized, Parallel Assignment	Yes	HRC
A novel model of care for Maori with chronic airways disease	2018	New Zealand	Maori	Respiratory Disease	Combination	All	16 Years and older	No published results	Non-Randomized, Single Group	Not stated	Other
Mana Tu: how effective is a whanau ora approach to improve HbA1C levels in people with poorly controlled type 2 diabetes mellitus	2018	New Zealand	Maori and/or Pacific	Diabetes	Behavioral	All	18 to 70 Years	No published results	Randomized, Parallel Assignment	Yes	HRC
OL@-OR@ trial: a cluster-randomised controlled trial to evaluate a co-designed, culturally-tailored, lifestyle-support mobile health (mHealth) tool, which includes an app and website for Maori and Pasifika in New Zealand	2018	New Zealand	Maori or Pasifika group	Cardiovascular Disease	Combination	All	18 Years and older	1451	Randomized, Parallel Assignment	Yes	Other
MyTeen – Increasing competence and mental health literacy: A mobile-	2018	New Zealand	Maori and Pacific Island	Mental illness	Behavioral	All	18 Years and older	47	Randomized, Parallel Assignment	Not stated	Other

based intervention to support parents of teenagers											
Testing an Integrated Care Model for Maori Men with Pre-diabetes, cardio vascular disease (CVD) risk, and/or obesity	2018	New Zealand	Maori	Cardiovascular Disease	Behavioral	Male	30-55 Years	Not completed	Non-randomized, Parallel Assignment	Not stated	Other
Evaluation of an app to support emotional wellbeing of adolescents experiencing depression and/or anxiety	2019	New Zealand	Maori and Pasifika	Mental illness	Device	All	11 to 16 Years	No published results	Randomized, Parallel Assignment	Yes	Other
Game for Health: Development of a Prototype eHealth Intervention to Treat Anxiety in Young People with Long-term Physical Conditions	2019	New Zealand	Maori	Mental illness	Behavioral	All	13 to18 Years	No published results	Non-randomized, Single group	Not stated	Other
Kia ora ai te Iwi - Whanau Oranga (wellbeing of the whole family): Supporting Maori with cultural-adapted psychological interventions to cope with the impact of Mate Wareware (Dementia).	2019	New Zealand	Maori	Mental illness	Behavioral	All	5 Years and above	No published results	Non-randomized	Yes	Other
Salt ALTernatives Study (SALTS): A smartphone app and dietary alternative salt to lower blood pressure for adults with high blood pressure	2019	New Zealand	Maori	Hypertension	Behavioral	All	18 Years and older	Not completed	Randomized, Parallel Assignment	Not stated	HRC
WORTH (which one is right here?): Randomised, crossover study to identify predictive baseline characteristics of response to pioglitazone or vildagliptin as second or third line therapy in patients with Type 2 diabetes	2019	New Zealand	Maori and Pacific	Diabetes	Drug	All	18 to 80 Years	Not completed	Randomized, Crossover Assignment	Yes	HRC
Understanding the uptake of a mobile phone application (app) to support youth wellbeing and resilience.	2019	New Zealand	Maori	Mental illness	Behavioral	All	12-22 Years	No published results	Non-randomized, Single group	Not stated	Other
A community group to improve loneliness in older adults	2020	New Zealand	Maori and non-Maori	Mental illness	Other	All	65 Years and older	Not completed	Randomized, Parallel Assignment	Yes	NHMRC
An randomised control trial of a cognitive behavioural therapy mobile health app	2020	New Zealand	Maori and Pacific	Mental illness	Behavioral	All	18 Years	Not completed	Randomized, Parallel Assignment	Not stated	HRC

[Manaaki] aimed at reducing gambling symptom severity and supporting people experiencing gambling problems.							and older				
The effect of health information distributed to people with knee osteoarthritis through community pharmacies: a feasibility study	2020	New Zealand	Maori or a Pacifica	Arthritis	Combination	All	18 Years and older	No published results	Randomized, Parallel Assignment	Not stated	HRC
Drum-Assisted Therapy for Native Americans	2010	US	Native American	Mental illness	Behavioral	All	18 to 65 Years	10	Randomized, Parallel Assignment	Yes	Other
Preventing Caries in Preschoolers: Testing a Unique Service Delivery Model in American Indian Head Start Programs	2011	US	American Indian	Dental disease	Combination	All	3 to 5 Years	1016	Randomized, Parallel Assignment	Yes	NIH
Promoting Behavioral Change for Oral Health in American Indian Mothers and Children	2011	US	American Indian	Dental disease	Behavioral	All	15 to 44 Years	1158	Randomized, Parallel Assignment	Yes	NIH
The Hanapū Study: Incentivized Partnerships to Reduce Diabetes Disparities	2011	US	Native and Pacific People	Diabetes	Other	All	18 Years and older	107	Randomized, Parallel Assignment	Yes	NIH
Together on Diabetes Study: Evaluation of a Pilot Diabetes Prevention and Management Program for American Indian Youth	2011	US	American Indian	Diabetes	Behavioral	All	10 to 19 Years	478	Single Group Assignment	Not stated	Other
Antiplatelet Effects of Ticagrelor Versus Clopidogrel in American Indian Patients	2012	US	American Indian	Cardiovascular Disease	Drug	All	18 Years and older	28	Randomized, Crossover Assignment	Not stated	Other
Ola Hou i ka Hula: Hula and Hypertension	2012	US	Native Hawaiians	Hypertension	Behavioral	All	18 Years and older	59	Randomized, Crossover Assignment	Yes	NIH
Cognitive Processing Intervention for Trauma, HIV/STI Risks, and Substance Use Among Native Women	2013	US	American Indian	Mental illness	Behavioral	Female	18 Years and older	60	Randomized, Crossover Assignment	Not stated	NIH
Factors Influencing Pediatric Asthma	2013	US	American Indian	Respiratory disease	Behavioral	All	6 to 17 Years	324	Randomized, Parallel Assignment	Yes	NIH

Home-base Kidney Care in Zuni Indians	2013	US	Indian	Kidney Disease	Other	All	21 to 80 Years	125	Randomized, Factorial Assignment	Yes	Other
Motivational Interviewing and Cognitive Behavioral Therapy-based Intervention for Cardiovascular Disease Prevention Amongst American Indians With Diabetic and Depressive Symptoms	2013	US	American Indian	Cardiovascular Disease	Behavioral	All	18 Years and older	34	Randomized, Parallel Assignment	Yes	NIH
Partnerships to Improve Lifestyle Interventions (PILI) 'Ohana Dissemination Project Partners in Care	2013	US	Native Hawaiian	Diabetes	Behavioral	All	18 Years and older	47	Single Group Assignment	Yes	Other
Vitamin D and Type 2 Diabetes Study	2013	US	American Indian, Alaska Native, Native Hawaiian or Other Pacific Islander	Diabetes	Drug	All	30 Years and older	No data on Indigenous enrollment	Randomized, Parallel Assignment	Not stated	NIH
Culturally Grounded Early Substance Use Prevention for American Indian Families	2013	US	American Indian	Mental illness	Behavioral	All	10-14 Years	449	Randomized, Factorial Assignment	Not stated	Other
Chronic Kidney Disease Knowledge and Awareness Among American Indians	2014	US	American Indian	Kidney Disease	Other	All	21 Years and older	70	Single Group Assignment	Not stated	NIH
Evaluation of an Entrepreneurship Program for American-Indian Youth	2014	US	American Indian	Mental illness	Behavioral	All	13 to 15 Years	393	Randomized, Parallel Assignment	Yes	Other
Iwankapiya-Healing: Historical Trauma Practice and Group IPT for American Indians	2014	US	American Indian	Mental illness	Behavioral	All	18 Years and older	52	Randomized, Parallel Assignment	Yes	NIH
Motivational Incentives for Alcohol Abstinence in American Indian and Native Alaskan Adults	2014	US	American Indian and Alaska Native	Mental illness	Behavioral	All	18 Years and older	Not completed	Randomized, Parallel Assignment	Not stated	Other
Motivational Interviewing and Culture for Urban Native American Youth (MICUNAY)	2014	US	Native American	Mental illness	Combination	All	14 to 18 Years	185	Randomized, Parallel Assignment	Yes	NIH
Wood Smoke Interventions in Native American Populations	2014	US	Native Americans	Respiratory disease	Combination	All	55 Years and older	156	Randomized, Parallel Assignment	Yes	NIH
Wood Stove Interventions and Child Respiratory Health	2014	US	American Indian and Alaska Native	Respiratory disease	Combination	All	up to 5 Years	516	Randomized, Parallel Assignment	Yes	NIH

Intertribal Talking Circle for the Prevention of Substance Abuse in Native Youth	2014	US	American Indian	Mental illness	Behavioral	All	12-55 Years	630	Randomized, Parallel Assignment	Yes	Other
Substance Use Prevention Campaign for American Indian Youth	2014	US	American Indian	Mental illness	Behavioral	All	11-14 Years	528	Randomized, Parallel Assignment	Yes	Other
A Patient-Centered Strategy for Improving Diabetes Prevention in Urban American Indians	2015	US	American Indian	Diabetes	Behavioral	All	21 Years and older	207	Randomized, Parallel Assignment Single	Yes	Other
An Intervention to Increase Engagement With Hypertension Care for American Indian Patients	2015	US	American Indian and Alaska Native	Hypertension	Behavioral	All	18 to 100 Years	No published results	Randomized, Parallel Assignment	Not stated	Other
Biomarker Feedback to Motivate Cessation in Pregnancy	2015	US	American Indian and Alaska Native	Mental illness	Behavioral	Female	18 to 45 Years	60	Randomized, Parallel Assignment	Yes	Other
Contingency Management for the Treatment of Co-Occurring Alcohol and Drug Misuse	2015	US	American Indian	Mental illness	Behavioral	All	18 Years and older	120	Randomized, Factorial Assignment	Not stated	Other
Growing Resilience in Wind River Indian Reservation	2015	US	Indigenous	Cardiovascular Disease	Other	All	5 to 80 Years	Not completed	Randomized, Parallel Assignment	Yes	NIH
Healing and Empowering Alaskan Lives Towards Healthy-Hearts Study	2015	US	Alaska Native	Mental illness	Behavioral	All	19 Years and older	No published results	Randomized, Parallel Assignment	Yes	NIH
Innovative Multigenerational Household Intervention to Reduce Stroke and CVD	2015	US	American Indian	Cardiovascular Disease	Behavioral	All	11 Years and older	458	Randomized, Parallel Assignment	Yes	Other
Yappalli - The Road to Choctaw Health	2015	US	Native	Mental illness	Behavioral	Female	18 Years and older	Not completed	Randomized, Crossover Assignment	Not stated	Other
The KaHOLO Project: Preventing Cardiovascular Disease in Native Hawaiians	2016	US	Native Hawaiian/Pacific Islanders	Hypertension	Behavioral	All	21 Years and older	275	Randomized, Crossover Assignment	Yes	NIH
The Development and Evaluation of the Ho'Ouna Pono Drug Prevention Curriculum	2016	US	Native Hawaiian and Pacific Islander	Mental illness	Behavioral	All	10-14 Years	486	Randomized, Parallel Assignment	Not stated	Other
A Family-Centered Ojibwe Substance Abuse Prevention	2017	US	American Indian	Mental illness	Behavioral	All	8 to 10 Years	Not completed	Randomized, Parallel Assignment	Yes	Other
American Indian and Alaska Native Men Who Have Sex	2017	US	American Indian and Alaska Native	Mental illness	Behavioral	Male	18 Years	79	Randomized, Parallel Assignment	Not stated	NIH

With Men HIV & Substance Abuse Research							and older				
An Asthma Collaboration to Reduce Childhood Asthma Disparities on the Navajo Nation	2017	US	Navajo Nation	Respiratory Disease	Behavioral	All	18 Years and older	Not completed	Non-Randomized, Sequential Assignment	Yes	Other
Controlling Hypertension in Native American and Other Populations	2017	US	American Indian and Alaska Native	Hypertension	Behavioral	All	21 to 79 Years	No published results	Randomized, Parallel Assignment	Not stated	NIH
Diet Intervention for Hypertension: Adaptation and Dissemination to Native Communities	2017	US	American Indians	Hypertension	Behavioral	All	18 Years and older	No published results	Randomized, Parallel Assignment	Not stated	NIH
Empaglifozin in Early Diabetic Kidney Disease	2017	US	American Indian	Diabetes	Drug	All	18 to 64 Years	0	Randomized, Parallel Assignment	Not stated	NIH
Home-Based Kidney Care in Native American's of New Mexico (HBKC)	2017	US	Native American	Kidney Disease	Behavioral	All	21 to 80 Years	Not completed	Randomized, Factorial Assignment	Yes	Other
Nxstan: A Culturally Tailored Intervention to Prevent Diabetes in American Indian Men	2017	US	American Indian	Diabetes	Behavioral	Male	21 to 65 Years	100	Randomized, Parallel Assignment	Yes	Other
Preventing Early Childhood Obesity, Part 1: Family Spirit Nurture, 3-9 Months	2017	US	American Indian	Cardiovascular Disease	Combination	Female	13 Years and older	136	Randomized, Parallel Assignment	Not stated	Other
Preventing HIV Among Native Americans Through the Treatment PTSD & Substance Use	2017	US	Native Americans	Mental illness	Behavioral	All	16 Years and older	Not completed	Randomized, Parallel Assignment	Yes	NIH
Testing Effectiveness of the Community Reinforcement Approach and Family Training (CRAFT) With American Indians	2017	US	American Indian	Mental illness	Behavioral	All	18 Years and older	No published results	Randomized, Parallel Assignment	Yes	NIH
Utilizing Traditional Practices	2017	US	American Indians and Alaska Natives	Mental illness	Behavioral	All	18 Years and older	62	Randomized, Parallel Assignment	Yes	NIH
Family Listening Program: Multi-Tribal Implementation and Evaluation	2017	US	Mescalero Apache, Jemez Pueblo and Ramah Navajo	Mental illness	Behavioral	All	8-11 Years	No published results	Non-randomized, Parallel Assignment	Yes	Other
Brief Interventions for the Prevention of Suicide and the Promotion of Resilience	2018	US	American Indian	Mental illness	Behavioral	All	10 to 24 Years	Not completed	Randomized, Sequential Assignment	Yes	NIH
Caring Contacts: A Strength-based, Suicide Prevention	2018	US	American Indians and Alaska Natives	Mental illness	Behavioral	All	18 Years	Not completed	Randomized, Parallel Assignment	Yes	NIH

Trial in 4 Native Communities							and older				
Chickasaw Healthy Eating Environments Research Study	2018	US	American Indian and Alaska Native	Hypertension	Behavioral	All	18 Years and older	Not completed	Randomized, Parallel Assignment	Yes	Other
Engaging Native Hawaiian/Pacific Islanders and Activating Communities to Take Steps (ENACTS)	2018	US	Native Hawaiian/Pacific Islanders	Hypertension	Behavioral	All	18 Years and older	Not completed	Randomized, Parallel Assignment	Not stated	NIH
Screening for Atrial Fibrillation in Native AmeRicans Using iPhone ECG	2018	US	Native American	Cardiovascular Disease	Screening	All	50 to 100 Years	Not completed	Single Group Assignment	Not stated	Other
Strong Men, Strong Communities Diabetes Risk Reduction in American Indian Men	2018	US	American Indian	Diabetes	Behavioral	Male	18 to 75 Years	Not completed	Randomized, Parallel Assignment	Yes	NIH
Supporting American Indian/Alaska Native Mothers and Daughters in Reducing Gestational Diabetes Risk	2018	US	American Indian and Alaska Native	Diabetes	Behavioral	Female	12 to 20 Years	398	Randomized, Parallel Assignment	Yes	NIH
Web-based Addiction Treatment: Cultural Adaptation With American Indians	2018	US	American Indian	Mental illness	Behavioral	All	18 to 99 Years	Not completed	Randomized, Single Group Assignment	Yes	NIH
Zero Suicide Plus KICKS	2018	US	American Indian	Mental illness	Behavioral	All	13 to 24 Years	Not completed	Randomized, Crossover Assignment	Yes	NIH
Promoting Community Conversations About Research to End Native Youth Suicide in Rural Alaska	2018	US	Native	Mental illness	Behavioral	All	15 Years and older	Not completed	Non-randomized, Parallel Assignment	Not stated	Other
Blood Pressure-Improving Control Among Alaska Native People" (BP-ICAN)	2019	US	Alaska Native	Hypertension	Device	All	18 Years and older	Not completed	Randomized, Parallel Assignment	Not stated	Other
Native-Changing High-risk Alcohol Use and Increasing Contraception Effectiveness Study	2019	US	American Indian and Alaska Native	Mental illness	Behavioral	Female	18 to 44 Years	Not completed	Randomized, Parallel Assignment	Not stated	Other
NCARE, Transition to Recovery	2019	US	Alaska Natives or American Indians.	Mental illness	Behavioral	All	18 Years and older	Not completed	Randomized, Single Group Assignment	Not stated	NIH
Southwest Hub for American Indian Youth Suicide Prevention Research	2019	US	American Indian	Mental illness	Combination	All	16 Years	Not completed	Randomized, Factorial Assignment	Yes	NIH

							and older				
Wa'Kan Ye'Zah: Enhancing Caregivers' and Children's Well-being Through an Evidence-based and Culturally Informed Prevention Intervention	2019	US	Native Americans	Mental illness	Behavioral	All	18 Years and older	Not completed	Randomized, Parallel Assignment	Yes	NIH
Cooking for Health	2020	US	American Indian	Diabetes	Behavioral	All	18 to 60 Years	Not completed	Randomized, Parallel Assignment	Not stated	NIH
Diabetes Nutrition Education for American Indian and Alaska Native Communities	2020	US	American Indian and Alaska Native	Diabetes	Behavioral	All	18 Years and older	Not completed	Randomized, Sequential Assignment	Not stated	Other
Evaluation of ThiwÃ¡he GluwÃ¡Å¡'Akapi Substance Use Prevention Program	2020	US	American Indian	Mental illness	Behavioral	All	10 to 85 Years	Not completed	Randomized, Factorial Assignment	Yes	Other
Pilot of IMPACT Intervention at the University of New Mexico	2020	US	American Indian	Kidney Disease	Behavioral	All	18 Years and older	Not completed	Randomized, Parallel Assignment	Not stated	Other
Reducing Diabetes Risk Factors in American Indian Children: Tribal Turning Point	2020	US	American Indian	Cardiovascular Disease	Combination	All	7 to 10 Years	Not completed	Randomized, Parallel Assignment	Not stated	NIH
Suicide in Urban Natives: Detection and Networks to Combat Events	2020	US	American Indian and Alaska Native	Mental illness	Behavioral	All	18 to 34 Years	Not completed	Randomized, Parallel Assignment	Not stated	NIH

Table S2. Summary of randomly selected studies.

Title	Conditions	Interventions	Actual Enrolled	Indigenous Enrolled	*Indigenous Population	Location	Study Designs	Funding	Start Year
"Wiihabilitation": Can active gaming systems be used to improve recovery after stroke?	Cardiovascular Disease	Rehabilitation	30		Race and Ethnicity Not Collected	Australia	Randomized, Parallel Assignment	NHMRC	2010
A Study to Evaluate the Potential Role of Mesenchymal Stem Cells in the Treatment of Idiopathic Pulmonary Fibrosis	Respiratory Disease	Other	8		Race and Ethnicity Not Collected	Australia	Single Group Assignment	Hospital	2010
Australian study of the effects of strict potassium restriction on nerve function in patients with chronic kidney disease	Kidney Disease	Other	47		Race and Ethnicity Not Collected	Australia	Randomized, Factorial Assignment	NHMRC	2010
Getting better at chronic care in North Queensland: A cluster randomized trial of patient-centred care delivered by Indigenous health professionals to Indigenous clients.	Diabetes	Behavioral	213	0		Australia	Non-randomized, Crossover Assignment	NHMRC	2010
Impact of routine screening and feedback on post-stroke depression	Mental Illness	Screening	124		Race and Ethnicity Not Collected	Australia	Randomized, Parallel Assignment	Industry	2010
Investigating Magnetic Seizure Therapy in Major Depressive Disorder.	Mental Illness	Device	40		Race and Ethnicity Not Collected	Australia	Randomized, Parallel Assignment	NHMRC	2010
Randomised controlled trial of two implementation methods of the e-couch Anxiety and Worry program in an adolescent school-based population.	Mental Illness	Behavioral	1767	53	Indigenous	Australia	Randomized, Parallel Assignment	Industry	2010
The CREDO Research Project: Can mood problems be prevented and treated using e-health interventions in patients being treated for cardiovascular disease?	Mental Illness	Other	562		Race and Ethnicity Not Collected	Australia	Randomized, Parallel Assignment	Industry	2010
Treatment of Bifurcation Lesions With The BIOTRONIK Pantera Lux Drug Eluting Balloon	Cardiovascular Disease	Device	35		Race and Ethnicity Not Collected	Australia	Single Group Assignment	Industry	2010
A randomised control trial of the impact of the implementation of case management compared with usual care in the transition of young adults with type 1 diabetes mellitus from paediatric to adult services	Diabetes	Other	120		Not Available	Australia	Randomized, Parallel Assignment	Research Institute	2011
A Randomised Controlled Trial of a Marinova Seaweed Extract on Osteoarthritis	Arthritis	Other	130		Not Available	Australia	Randomized, Parallel Assignment	Industry	2011
Cognitive Behavioural Therapy for Vestibular Migraine	Mental Illness	Behavioral	61		Not Available	Australia	Randomized, Parallel Assignment	Hospital	2011
Does cholecalciferol (vitamin D3) improve patient-level outcomes for people with chronic kidney disease on dialysis	Kidney Disease	Drug	60		Race and Ethnicity Not Collected	Australia	Randomized, Parallel Assignment	Industry	2011
Intravitreal Aflibercept Injection in Vision Impairment Due to DME	Diabetes	Procedure	404		Race and Ethnicity Not Collected	Australia	Randomized, Crossover Assignment	Industry	2011

Mindfulness, cognitive processes and coping in chronic illness: insights from a study of joint replacement surgery	Arthritis	Behavioral	145		Race and Ethnicity Not Collected	Australia	Randomized, Parallel Assignment	Research Institute	2011
The effect of physiotherapy booster sessions on home exercise outcomes in people with knee osteoarthritis	Arthritis	Rehabilitation	78		Race and Ethnicity Not Collected	Australia	Randomized, Parallel Assignment	NHMRC	2011
The Osteoarthritis Stem Cell Advanced Research Study	Arthritis	Other	40		Not Available	Australia	Randomized	Industry	2011
The Out-and-About trial: Improving quality of life after stroke	Cardiovascular Disease	Behavioral	263		Not Available	Australia	Randomized, Parallel Assignment	NHMRC	2011
Treatment of Cardiovascular Risk in Primary Care with Electronic Decision Support- The TORPEDO study.	Cardiovascular Disease	Screening	38725	6916	Aboriginal and Torres Strait Islander	Australia	Randomized, Parallel Assignment	NHMRC	2011
A randomised trial assessing the acceptability and effectiveness of providing generic versus tailored feedback about health risks for a high need primary care sample	Cardiovascular Disease	Behavioral	87	62	Aboriginal	Australia	Non-Randomized, Parallel Assignment	Industry	2012
Effect of long term consumption of Australian pork for weight loss and weight maintenance on cardiometabolic health, food cravings and cognition and psychological wellbeing in individuals with type 2 diabetes.	Diabetes	Behavioral	61		Race and Ethnicity Not Collected	Australia	Randomized, Parallel Assignment	Industry	2012
Mitochondrial agents in the treatment of bipolar disorder	Mental Illness	Drug	181		Race and Ethnicity Not Collected	Australia	Randomized, Parallel Assignment	NHMRC	2012
Sitting Time AfteR Stroke (STARS). A phase II safety and feasibility trial of counselling sessions aimed at encouraging stroke survivors to 'move more and sit less'.	Cardiovascular Disease	Behavioral	35		Race and Ethnicity Not Collected	Australia	Randomized, Parallel Assignment	Industry	2012
Stepping Up Study: A Cluster Randomised Controlled Trial of team-based transition to insulin in primary care compared to usual care to improve HbA1c for patients with poorly controlled type 2 diabetes	Diabetes	Other	266		Race and Ethnicity Not Collected	Australia	Randomized, Parallel Assignment	NHMRC	2012
Aged Garlic Extract for heart health: A 3-month randomised placebo-controlled trial	Hypertension	Drug	100		Race and Ethnicity Not Collected	Australia	Randomized, Parallel Assignment	Industry	2013
Couselling to improve symptoms of anxiety or depression for heart transplant recipients	Mental Illness	Behavioral	13		Race and Ethnicity Not Collected	Australia	Randomized, Parallel Assignment	University	2013
Home Monitoring of Chronic Disease for Aged Care	Cardiovascular Disease	Device	287		Race and Ethnicity Not Collected	Australia	Non-randomized, Quasi Case-Control Design	Industry	2013
Implementing care coordination plus early rehabilitation in high-risk chronic obstructive pulmonary disease (COPD) patients in transition from hospital to primary care	Respiratory Disease	Other	19		Race and Ethnicity Not Collected	Australia	Non-randomized	University	2013
Modified SHoes for osteoARthritis of the Knee: the SHARK study	Arthritis	Device	160		Not Available	Australia	Randomized, Parallel Assignment	NHMRC	2013

Online treatment for depression in people with diabetes: a randomised controlled trial	Mental Illness	Behavioral	91		Race and Ethnicity Not Collected	Australia	Randomized, Parallel Assignment	Hospital	2013
Online treatment for depression in people with osteoarthritis: a randomised controlled trial	Mental Illness	Behavioral	77		Not Available	Australia	Randomized, Parallel Assignment	Hospital	2013
Screening Education And Recognition by primary Care pHysician of Atrial Fibrillation for prevention of stroke (SEARCH-AF II)	Cardiovascular Disease	Screening	88		Not Available	Australia	Single Group Assignment	Industry	2013
Technology That Permits Focal Dose of Antibiotics to be Delivered to Lower Limb(s) of Diabetic Patients	Diabetes	Drug	15		Race and Ethnicity Not Collected	Australia	Randomized, Parallel Assignment	Industry	2013
What is the efficacy of therapist-guided internet-treatment, self-guided internet-treatment with pre-treatment contact, and purely self-guided internet-delivered for older adults (60+) with symptoms of anxiety and depression?	Mental Illness	Behavioral	434		Race and Ethnicity Not Collected	Australia	Randomized, Parallel Assignment	Industry	2013
Magnesium supplements for the treatment of resistant depression	Mental Illness	Drug	10		Not Available	Australia	Single Group Assignment	University	2014
Multimorbidity rehabilitation in chronic disease: general rehabilitation compared to usual care	Cardiovascular Disease	Rehabilitation	16		Not Available	Australia	Randomized, Parallel Assignment	Research Institute	2014
Prescribing the maximum tolerated dose of walking for people with severe knee osteoarthritis: A Phase II, Randomised Controlled Trial	Arthritis	Rehabilitation	46		Race and Ethnicity Not Collected	Australia	Randomized, Parallel Assignment	University	2014
TriPoD: Trial for the Prevention of Depression in final year secondary school students	Mental Illness	Behavioral	540		Race and Ethnicity Not Collected	Australia	Randomized	NHMRC	2014
A Study of ARC-AAT in Healthy Volunteer Subjects and Patients With Alpha-1 Antitrypsin Deficiency (AATD)	Respiratory Disease	Drug	65		Race and Ethnicity Not Collected	Australia	Randomized, Parallel Assignment	Industry	2015
A Study to Compare Insulin Intensification of Biphasic Insulin Aspart 30 and Insulin Analogues (Insulin Glargine and Insulin Aspart) in Insulin naÃ¯ve Type 2 Diabetic Patients	Diabetes	Drug	335		Race and Ethnicity Not Collected	Australia	Randomized, Parallel Assignment	Industry	2015
An evaluation of the efficacy of curcumin and saffron for the treatment of depression	Mental Illness	Other	160		Race and Ethnicity Not Collected	Australia	Randomized, Parallel Assignment	Industry	2015
Canakinumab Add-on Treatment in Schizophrenia (CATS)	Mental Illness	Drug	29		Race and Ethnicity Not Collected	Australia	Randomized, Parallel Assignment	University	2015
Efficacy of Dexamethasone in reducing pain, nausea and vomiting, improve mobilisation and reduce hospital stay in hip and knee arthroplasty: a double blind controlled trial	Arthritis	Drug	142		Race and Ethnicity Not Collected	Australia	Randomized, Parallel Assignment	Research Institute	2015
Low Energy Therapy to Convert Ventricular Tachycardias	Cardiovascular Disease	Device	9		Race and Ethnicity Not Collected	Australia	Single Group Assignment	Industry	2015
The effectiveness of kinesiology taping for shoulder on pain and motor control in patients after stroke	Cardiovascular Disease	Other	10		Not Available	Australia	Randomized, Parallel Assignment	Individual	2015

The Health Anxiety Program: a pilot trial of an online program for people who worry excessively about their health	Mental Illness	Behavioral	16		Race and Ethnicity Not Collected	Australia	Single Group Assignment	Industry	2015
The Resilience at Work (RAW) Mindfulness Program: The development and evaluation of an online mindfulness based training program aimed at enhancing psychological resilience and wellbeing among Emergency Workers	Mental Illness	Behavioral	143		Race and Ethnicity Not Collected	Australia	Randomized, Parallel Assignment	Research Institute	2015
An evaluation of user acceptance and performance of a mobile real-time continuous glucose monitoring system.	Diabetes	Device	10		Not Available	Australia	Randomized, Crossover Assignment	Hospital	2016
Consumer Choice of Pre-Packaged Foods that Vary by Nutrition Information and Price	Diabetes	Behavioral	2067		Race and Ethnicity Not Collected	Australia	Randomized, Crossover Assignment	University	2016
Efficacy and safety of a single injection of Articul One in comparison with Synvisc-One Registered Trademark or placebo for the treatment of symptomatic primary knee osteoarthritis.	Arthritis	Drug	30		Not Available	Australia	Randomized, Parallel Assignment	Industry	2016
Fish Oil Cell Uptake Study of Inflammation – a trial of fish oil supplementation in healthy volunteers	Cardiovascular Disease	Dietary Supplement	40		Not Available	Australia	Randomized, Parallel Assignment	Individual	2016
General Practice Optimising Structured MOnitoring To Improve Clinical outcomes in Type 2 Diabetes: An individually randomised trial of the effect of retrospective continuous glucose monitoring (rCGM) for people with type 2 diabetes in general practice on HbA1.	Diabetes	Device	299		Not Available	Australia	Randomized, Parallel Assignment	NHMRC	2016
The effectiveness of a stretching intervention in lowering plantar pressures related to reduced ankle range of motion in people with diabetes.	Diabetes	Behavioral	68		Not Available	Australia	Randomized, Parallel Assignment	University	2016
The Evaluation of iNdividualized Telehealth Intensive Coaching to promote healthy Eating and lifestyle in Chronic Kidney Disease	Kidney Disease	Behavioral	82	1	Indigenous	Australia	Randomized, Parallel Assignment	Research Institute	2016
The MIRROR2 pilot study: prevention of Major depressIon among older people living in Regional and RemOte aReas of Western Australia	Mental Illness	Behavioral	309		Not Available	Australia	Randomized, Parallel Assignment	University	2016
What is the optimal dose of insulin for the protein content of a meal in individuals with type 1 diabetes mellitus using intensive insulin therapy	Diabetes	Other	32		Not Available	Australia	Randomized	Hospital	2016
A 12 week pilot study targeted for adult consumers within a community mental health rehabilitation setting to implement a Personal Safety Tool for self-management and crisis management.	Mental Illness	Behavioral	7		Not Available	Australia	Non-randomized	Industry	2017
A phase 2b, randomised double blind placebo controlled multicentre study to evaluate the effects of pentosan polysulfate sodium on treating pain in subjects with osteoarthritis of the knee and subchondral bone marrow lesions	Arthritis	Drug	126		Race and Ethnicity Not Collected	Australia	Randomized, Parallel Assignment	Industry	2017
Ambulatory oxygen in interstitial lung disease	Respiratory Disease	Device	30		Race and Ethnicity Not Collected	Australia	Randomized, Parallel Assignment	Industry	2017

Embedding High Intensity Interval Training (HIIT) into the school day: The Burn 2 Learn pilot trial for senior school students	Diabetes	Behavioral	68	0		Australia	Randomized, Parallel Assignment	NHMRC	2017
Examining the Feasibility of an Online Cognitive Behaviour Therapy (CBT) - based self-management Program for Adults with Neurological Conditions.	Mental Illness	Behavioral	105		Race and Ethnicity Not Collected	Australia	Single Group Assignment	University	2017
Goal oriented instructions increase the number of repetitions completed in stroke rehabilitation: A within-participant, repeated measures experimental study	Cardiovascular Disease	Rehabilitation	24		Race and Ethnicity Not Collected	Australia	Randomized, Parallel Assignment	University	2017
Nutritional supplements for prevention of type 2 diabetes - postprandial study	Diabetes	Behavioral	16		Race and Ethnicity Not Collected	Australia	Randomized, Crossover Assignment	University	2017
Development and evaluation of a nursing risk assessment manual handling training program for moving patients with a stroke and other conditions affecting mobility: The Risk Assessment for Moving Patients (RAMP) program	Cardiovascular Disease	Behavioral	72		Not Available	Australia	Single Group Assignment	Industry	2018
Efficacy of saffron as an adjunct treatment for unremitted depression in adults	Mental Illness	Other	160		Race and Ethnicity Not Collected	Australia	Randomized, Parallel Assignment	Industry	2018
Beta Blockers and Angiotensin Receptor Blockers in Bicuspid Aortic Valve Disease Aortopathy (BAV Study)	Cardiovascular Disease	Drug	85	0		Canada	Randomized, Parallel Assignment	Research Institute	2011
Bystander Fatigue and CPR Quality Using Continuous Compressions Versus 30:2 Compressions to Ventilation	Cardiovascular Disease	Procedure	63		Race and Ethnicity Not Collected	Canada	Randomized, Crossover Assignment	Research Institute	2011
Comparison of the Bioavailability of Metformin Between Medium Dose Linagliptin/Metformin Tablets and Medium Dose Glucophage Tablet Given With Linagliptin Tablet	Diabetes	Drug	58		Race and Ethnicity Not Collected	Canada	Randomized, Crossover Assignment	Industry	2011
Effect of Travoprost 0.004% on Retinal Oximetry in Primary Open Angle Glaucoma	Hypertension	Drug	16		Race and Ethnicity Not Collected	Canada	Non-randomized, Parallel Assignment	University	2012
Improving Oral Care to Reduce Hospital-Acquired Pneumonia (HAP) in the Acute Neurologically Impaired Adult	Respiratory Disease	Other	32		Race and Ethnicity Not Collected	Canada	Single Group Assignment	Industry	2012
52 Week Trial of Liraglutide in Type 1 Diabetes	Diabetes	Drug	15		Race and Ethnicity Not Collected	Canada	Randomized, Parallel Assignment	University	2013
The Family Cognitive Adaptation Training Manual: A Test of Effectiveness	Mental Illness	Behavioral	40	0		Canada	Randomized, Parallel Assignment	Industry	2013
A Study of Single or Repeated Doses of Glucagon in Participants With Diabetes	Diabetes	Drug	32	0		Canada	Randomized, Crossover Assignment	Industry	2015
MONITOR-OA: Using Wearable Activity Trackers to Improve Physical Activity in Knee Osteoarthritis	Arthritis	Behavioral	61		Race and Ethnicity Not Collected	Canada	Randomized, Crossover Assignment	University	2015

The INDORSE Study: Inhibition of Dipeptidyl Peptidase IV: Outcomes on Renal Sodium Excretion	Diabetes	Other	32		Race and Ethnicity Not Collected	Canada	Randomized, Parallel Assignment	Hospital	2015
Feasibility Study of DermGEN for Diabetic Foot Ulcer Treatment	Diabetes	Biological	11		Race and Ethnicity Not Collected	Canada	Single Group Assignment	CIHR	2016
To Investigate the Efficacy of an Occluding Dentifrice in Dentinal Hypersensitivity (DH)	Dental Disease	Other	192	0		Canada	Randomized, Parallel Assignment	Industry	2016
Utilizing Novel Dipole Density Capabilities to Objectively Visualize the Etiology of Rhythms in Atrial Fibrillation	Cardiovascular Disease	Device	129	0		Canada	Single Group Assignment	Industry	2016
Feasibility and Outcomes of a Digital Health Support for the Schizophrenia Spectrum	Mental Illness	Device	38	0		Canada	Single Group Assignment	Industry	2017
A Study to Evaluate the Long-Term Safety of MEDI-545 in Adult Participants With Systemic Lupus Erythematosus or Myositis	Arthritis	Drug	103		Race and Ethnicity Not Collected	Multiple	Single Group Assignment	Industry	2010
An Efficacy, Safety, and Tolerability Study of Canagliflozin in Patients With Type 2 Diabetes Mellitus Who Have Moderate Renal Impairment	Diabetes	Drug	272		Race and Ethnicity Not Collected	Multiple	Randomized, Parallel Assignment	Research Institute	2010
An Open-Label, Multicenter, Rollover, Long-term Study of Aripiprazole Intramuscular Depot in Participants With Schizophrenia	Mental Illness	Drug	709	2	American Indian or Alaska Native, Native Hawaiian or Other Pacific Islander	Multiple	Single Group Assignment	Industry	2010
BI 10773 (Empagliflozin) Cardiovascular Outcome Event Trial in Type 2 Diabetes Mellitus Patients (EMPA-REG OUTCOME).	Diabetes	Drug	7028	0		Multiple	Randomized, Parallel Assignment	Industry	2010
CALIPSO: Calfactant for Acute Lung Injury in Pediatric Stem Cell Transplant and Oncology Patients	Respiratory Disease	Drug	43	1	American Indian or Alaska Native	Multiple	Randomized, Parallel Assignment	University	2010
Clinical Trials to Reduce the Risk of Antimicrobial Resistance	Respiratory Disease	Drug	43		Race and Ethnicity Not Collected	Multiple	Randomized, Parallel Assignment	University	2010
Efficacy and Safety of 3 Doses of Tiotropium Compared to Placebo in Adolescents (12 to 17 Yrs) With Moderate Asthma	Respiratory Disease	Drug	105	0		Multiple	Randomized, Crossover Assignment	Industry	2010
Evaluation of Cardiovascular Outcomes in Patients With Type 2 Diabetes After Acute Coronary Syndrome During Treatment With AVE0010 (Lixisenatide)	Diabetes	Drug	6068	0		Multiple	Randomized, Parallel Assignment	Industry	2010
Evaluation of the Pharmacodynamic Effect of the Combination of Sildenafil and Riociguat on Blood Pressure and Other Safety Parameters.	Hypertension	Drug	18	0		Multiple	Randomized, Parallel Assignment	Industry	2010
Evaluation of Tiotropium 2.5 and 5 mcg Once Daily Delivered Via the RespimatÂ® Inhaler Compared to Placebo and Salmeterol HydroFluoroAlkane (HFA) Metered Dose Inhaler (MDI) (50 mcg Twice Daily) in Patient With Moderate Persistent Asthma II	Respiratory Disease	Drug	1032	118	American Indian or Alaska Native	Multiple	Randomized, Parallel Assignment	Industry	2010

FAME II - Fractional Flow Reserve (FFR) Guided Percutaneous Coronary Intervention (PCI) Plus Optimal Medical Treatment (OMT) Verses OMT	Cardiovascular Disease	Other	1170		Race and Ethnicity Not Collected	Multiple	Randomized, Parallel Assignment	Industry	2010
Impact of Dietary Intervention on Weight Change in Subjects With Type 2 Diabetes	Diabetes	Dietary Supplement	611	3	Native Hawaiian or Other Pacific Islander	Multiple	Randomized, Parallel Assignment	Industry	2010
Kidney Damage in Patients With Moderate Fall in eGFR	Cardiovascular Disease	Drug	57		Race and Ethnicity Not Collected	Multiple	Randomized, Parallel Assignment	Industry	2010
Phase II Study of Afinitor vs. Sutent in Patients With Metastatic Non-Clear Cell Renal Cell Carcinoma	Kidney Disease	Drug	131	0		Multiple	Randomized, Parallel Assignment	University	2010
Safety and Efficacy of AIN457 in Patients With Quiescent Non-infectious Uveitis	Arthritis	Drug	125		Race and Ethnicity Not Collected	Multiple	Randomized, Parallel Assignment	Industry	2010
Study Comparing Synthetic Vascular Grafts in Patients With Peripheral Artery Disease (PAD) Who Require Artery Bypass.	Cardiovascular Disease	Device	207	0		Multiple	Randomized, Parallel Assignment	Industry	2010
The CANTATA-MP Trial (CANagliflozin Treatment and Trial Analysis - Metformin and Pioglitazone)	Diabetes	Drug	344		Race and Ethnicity Not Collected	Multiple	Randomized, Parallel Assignment	Research Institute	2010
An Exercise Endurance Study to Evaluate the Effects of Treatment of Chronic Obstructive Pulmonary Disease (COPD) Patients With a Dual Bronchodilator: GSK573719/GW642444. Study A	Respiratory Disease	Drug	307	1	American Indian or Alaska Native	Multiple	Randomized, Crossover Assignment	Industry	2011
AugmentÂ® Injectable Bone Graft Compared to Autologous Bone Graft as a Bone Regeneration Device in Hindfoot Fusions	Arthritis	Device	299		Race and Ethnicity Not Collected	Multiple	Randomized, Parallel Assignment	Industry	2011
Aztreonam Lysine for Pseudomonas Infection Eradication Study	Respiratory Disease	Drug	105	0		Multiple	Single Group Assignment	Industry	2011
Clinical Trial Evaluating TechnosphereÂ® Insulin Versus Insulin Aspart in Subjects With Type 1 Diabetes Mellitus Over a 24-week Treatment Period	Diabetes	Drug	518	1	Native Hawaiian or Other Pacific Islander	Multiple	Randomized, Parallel Assignment	Industry	2011
Continuous Soluble Ferric Pyrophosphate (SFP) Iron Delivery Via Dialysate in Hemodialysis Patients	Kidney Disease	Drug	108	0		Multiple	Randomized, Parallel Assignment	Industry	2011
DysportÂ® Adult Upper Limb Spasticity Extension Study	Cardiovascular Disease	Biological	258	0		Multiple	Single Group Assignment	Industry	2011
Effect of Liraglutide on Body Weight in Overweight or Obese Subjects With Type 2 Diabetes: SCALEâ„¢ - Diabetes	Diabetes	Drug	846	4	American Indian or Alaska Native	Multiple	Randomized, Parallel Assignment	Industry	2011
Effects of Two Dosing Regimens of Bosentan in Children With Pulmonary Arterial Hypertension	Respiratory Disease	Drug	64		Race and Ethnicity Not Collected	Multiple	Randomized, Parallel Assignment	Industry	2011
Efficacy at 24 Weeks and Long Term Safety, Tolerability and Efficacy up to 2 Years of Secukinumab (AIN457) in Patients With Active Psoriatic Arthritis (PsA)	Arthritis	Drug	606		Race and Ethnicity Not Collected	Multiple	Randomized, Parallel Assignment	Industry	2011

Epoprostenol for Injection (EFI/ACT-385781A) - Pulmonary Arterial Hypertension	Respiratory Disease	Drug	42	0		Multiple	Single Group Assignment	Industry	2011
MEmbranous Nephropathy Trial Of Rituximab	Kidney Disease	Drug	130	0		Multiple	Single Group Assignment	University	2011
Open Label Extension Study of Epratuzumab in Subjects With Systemic Lupus Erythematosus	Arthritis	Drug	1250		Race and Ethnicity Not Collected	Multiple	Randomized, Parallel Assignment	Industry	2011
PEARL Schizophrenia Maintenance	Mental Illness	Drug	676	4	American Indian or Alaska Native, Native Hawaiian or Pacific Islander	Multiple	Randomized, Parallel Assignment	Industry	2011
Phase I Biomarker Study (BMS-936558)	Kidney Disease	Drug	119	0		Multiple	Randomized, Parallel Assignment	Industry	2011
Roflumilast in Chronic Obstructive Pulmonary Disease (COPD) Patients Treated With Fixed Combinations of Long-acting Î²2-agonists (LABA) and Inhaled Glucocorticosteroid (ICS)	Respiratory Disease	Drug	1945	0		Multiple	Randomized, Parallel Assignment	Industry	2011
Safety & Efficacy of BCT197 in Patients Undergoing Cardiac Surgery	Kidney Disease	Drug	91	0		Multiple	Randomized, Parallel Assignment	Industry	2011
Safety and Efficacy of Levomilnacipran ER (Levomilnacipran SR) in Major Depressive Disorder	Mental Illness	Drug	568	4	American Indian or Alaska Native, Native Hawaiian or Other Pacific Islander	Multiple	Randomized, Parallel Assignment	Industry	2011
Safety, Tolerability, and Efficacy of Cariprazine in Participants With Bipolar Depression	Mental Illness	Drug	584	7	American Indian or Alaska Native	Multiple	Randomized, Parallel Assignment	Industry	2011
Study Of A Controlled Release Formulation Of Pregabalin In Fibromyalgia Patients	Arthritis	Drug	441	0		Multiple	Randomized, Parallel Assignment	Industry	2011
Study to Evaluate the 24-Hour Pulmonary Function Profile of Fluticasone Furoate (FF) /GW642444 (Vilanterol) (VI) Inhalation Powder 100/25mcg Once Daily Compared With Fluticasone Propionate/Salmeterol Inhalation Powder 250/50mcg Twice Daily in Subjects With Chronic Obstructive Pulmonary Disease	Respiratory Disease	Drug	519	1	American Indian or Alaska Native	Multiple	Randomized, Parallel Assignment	Industry	2011
Study to Evaluate the Efficacy and Safety of the Combination of Valturna and Amlodipine or Valturna and Chlorthalidone Versus Valturna Alone in Patients With Stage 2 Hypertension and Diabetes	Diabetes	Drug	975		Race and Ethnicity Not Collected	Multiple	Randomized, Parallel Assignment	Industry	2011

Study to Evaluate the Efficacy, Safety, Tolerability, and Pharmacokinetics of Saxagliptin as Monotherapy in Pediatric Patients With Type 2 Diabetes	Diabetes	Drug	26		Race and Ethnicity Not Collected	Multiple	Randomized, Parallel Assignment	Industry	2011
A Trial of ASP7487 (OSI-906) in Combination With Bortezomib for the Treatment of Relapsed Multiple Myeloma	Cardiovascular Disease	Drug	19	0		Multiple	Single Group Assignment	Hospital	2012
BIIB023 Proof-of-Concept Study in Participants With Lupus Nephritis	Arthritis	Biological	276		Race and Ethnicity Not Collected	Multiple	Randomized, Parallel Assignment	Industry	2012
Daily Use of Lipikar Balm AP From Birth in Infants at High Risk of Developing Atopic Dermatitis	Respiratory Disease	Drug	2		Race and Ethnicity Not Collected	Multiple	Randomized, Parallel Assignment	Industry	2012
Effects of Ventavis in Patients With Pulmonary Hypertension (PH) Secondary to Chronic Obstructive Pulmonary Disease (COPD)	Respiratory Disease	Drug	2		Race and Ethnicity Not Collected	Multiple	Randomized, Parallel Assignment	Industry	2012
Efficacy and Safety of QMF149 vs. Salmeterol Xinafoate/Fluticasone Propionate in Patients With Chronic Obstructive Pulmonary Disease (COPD)	Respiratory Disease	Drug	629	0		Multiple	Randomized, Parallel Assignment	Industry	2012
Efficacy, Safety and Tolerability of Two Fixed Dose Combinations of Aclidinium Bromide/Formoterol Fumarate, Aclidinium Bromide, Formoterol Fumarate and Placebo for 28-Weeks Treatment in Patients With Moderate to Severe, Stable Chronic Obstructive Pulmonary Disease (COPD)	Respiratory Disease	Drug	921	0		Multiple	Randomized, Parallel Assignment	Industry	2012
Evaluation of Safety and Efficacy, Including Pharmacokinetics, of NNC 0129-0000-1003 When Administered for Treatment and Prophylaxis of Bleeding in Subjects With Haemophilia A	Cardiovascular Disease	Drug	186	0		Multiple	Non-randomized, Parallel Assignment	Industry	2012
Heart And Lung Failure - Pediatric INsulin Titration Trial	Cardiovascular Disease	Drug	713	0		Multiple	Randomized, Parallel Assignment	NIH	2012
Phase 3b Safety and Efficacy Study of Apremilast to Treat Moderate to Severe Plaque-plaque Psoriasis	Arthritis	Drug	250	0		Multiple	Randomized, Parallel Assignment	Industry	2012
Phase III Clinical Worsening Study of UT-15C in Subjects With PAH Receiving Background Oral Monotherapy	Respiratory Disease	Drug	690	0		Multiple	Randomized, Parallel Assignment	Industry	2012
Safety, Efficacy and Pharmacokinetics of NNC-0156-0000-0009 in Previously Treated Children With Haemophilia B.	Cardiovascular Disease	Drug	25	0		Multiple	Single Group Assignment	Industry	2012
Study of Ivacaftor in Subjects With Cystic Fibrosis (CF) Who Have the R117H-CF Transmembrane Conductance Regulator (CFTR) Mutation (KONDUCT)	Respiratory Disease	Drug	69		Race and Ethnicity Not Collected	Multiple	Randomized, Parallel Assignment	Industry	2012
Study of LY2886721 in Mild Cognitive Impairment Due to Alzheimer's Disease or Mild Alzheimer's Disease	Mental Illness	Drug	70	0		Multiple	Randomized, Parallel Assignment	Industry	2012
Study of Pomalidomide (CC-4047) to Evaluate Safety, Tolerability, Pharmacokinetics, Pharmacodynamics and Effectiveness for Patients With Systemic Sclerosis With Interstitial Lung Disease	Arthritis	Drug	23	1	Native Hawaiian or Other Pacific Islander	Multiple	Randomized, Parallel Assignment	Industry	2012

Study of the Effect of Fostamatinib Twice Daily on Blood Pressure in Patients With Rheumatoid Arthritis	Arthritis	Drug	266	0		Multiple	Randomized, Parallel Assignment	Industry	2012
Treatment of Resistant Hypertension Using a Radiofrequency Percutaneous Transluminal Angioplasty Catheter	Hypertension	Device	146	0		Multiple	Single Group Assignment	Industry	2012
An Evaluation of Sarilumab Plus Methotrexate Compared to Etanercept Plus Methotrexate in RA Patients Not Responding to Adalimumab Plus Methotrexate	Arthritis	Drug	776		Race and Ethnicity Not Collected	Multiple	Randomized, Parallel Assignment	Industry	2013
Blinded Safety & Efficacy Placebo Controlled Study of Icatibant for Angiotensin Converting Enzyme Inhibitor Induced Angioedema	Cardiovascular Disease	Drug	121	0		Multiple	Randomized, Parallel Assignment	Industry	2013
Clinical Study to Assess the Long-term Safety, Tolerability, and Efficacy of Macitentan in Subjects With Eisenmenger Syndrome	Hypertension	Drug	217	0		Multiple	Single Group Assignment	Industry	2013
Clinical Study to Evaluate the Effects of Macitentan on Exercise Capacity in Subjects With Eisenmenger Syndrome	Hypertension	Drug	226	0		Multiple	Randomized, Parallel Assignment	Industry	2013
Comparison of Fasiglifam (TAK-875) With Sitagliptin When Used in Combination With Metformin in Patients With Type 2 Diabetes	Diabetes	Drug	96	1	Native Hawaiian or Other Pacific Islander	Multiple	Randomized, Parallel Assignment	Industry	2013
Efficacy, Safety and Pharmacokinetics Study of Antroquinonol to Treat NSCLC	Respiratory Disease	Drug	31	0		Multiple	Single Group Assignment	Industry	2013
Evaluate the Effect of Aclidinium Bromide on Long-term Cardiovascular Safety and Exacerbations in Moderate to Very Severe COPD Patients.	Respiratory Disease	Drug	3635	12	American Indian or Alaska Native, Native Hawaiian or Other Pacific Islander	Multiple	Randomized, Parallel Assignment	Industry	2013
Gadobutrol / Gadavist-enhanced Cardiac Magnetic Resonance Imaging (CMRI) to Detect Coronary Artery Disease (CAD)	Cardiovascular Disease	Drug	426	0		Multiple	Single Group Assignment	Industry	2013
Multicenter Trial to Evaluate the Effect of Dapagliflozin on the Incidence of Cardiovascular Events	Diabetes	Drug	17190	126	American Indian or Alaska Native, Native Hawaiian or Other Pacific Islander	Multiple	Randomized, Parallel Assignment	Industry	2013
nMARQâ„¢ Pulmonary Vein Isolation System for the Treatment of Paroxysmal Atrial Fibrillation	Cardiovascular Disease	Device	481	3	American Indian or Alaska Native	Multiple	Randomized, Parallel Assignment	Industry	2013
Open Label, Phase II Study to Evaluate Efficacy and Safety of Oral Nilotinib in Philadelphia Positive (Ph+) Chronic Myelogenous Leukemia (CML) Pediatric Patients.	Cardiovascular Disease	Drug	59	1	Native Americans	Multiple	Single Group Assignment	Industry	2013

Pediatric Schizophrenia Efficacy and Safety Study	Mental Illness	Drug	327	0		Multiple	Randomized, Parallel Assignment	Industry	2013
Rivaroxaban for the Prevention of Major Cardiovascular Events in Coronary or Peripheral Artery Disease	Cardiovascular Disease	Drug	27395	0		Multiple	Randomized, Parallel Assignment	Industry	2013
Safety and Immunogenicity of Two Doses of H5N1 Influenza Vaccine in Healthy Adults	Respiratory Disease	Biological	979	4	American Indian or Alaska Native, Native Hawaiian or Other Pacific Islander	Multiple	Randomized, Parallel Assignment	Industry	2013
Stenting of the Superficial Femoral and/or Proximal Popliteal Artery Project	Cardiovascular Disease	Device	57	0		Multiple	Single Group Assignment	Industry	2013
The Evaluation of Bococizumab (PF-04950615; RN316) in Reducing the Occurrence of Major Cardiovascular Events in High Risk Subjects	Cardiovascular Disease	Drug	10564	0		Multiple	Randomized, Parallel Assignment	Industry	2013
Acetylsalicylic Acid Compared to Placebo in Treating High-Risk Patients With Subsolid Lung Nodules	Respiratory Disease	Drug	109	0		Multiple	Randomized, Parallel Assignment	NIH	2014
An Efficacy And Safety Study Evaluating Tofacitinib With And Without Methotrexate Compared To Adalimumab With Methotrexate	Arthritis	Drug	1152		Race and Ethnicity Not Collected	Multiple	Randomized, Parallel Assignment	Industry	2014
Clinical Mismatch in the Triage of Wake Up and Late Presenting Strokes Undergoing Neurointervention With Trevo	Cardiovascular Disease	Device	206	1	Native Hawaiian or other Pacific Islander	Multiple	Randomized, Parallel Assignment	Industry	2014
Comparison of CHS-0214 to Enbrel (Etanercept) in Patients With Rheumatoid Arthritis (RA)	Arthritis	Drug	647	0		Multiple	Randomized, Parallel Assignment	Industry	2014
Double-Blind, Multiple Ascending Dose Study to Assess Safety, Tolerability and Pharmacokinetics of DX-2930 in Hereditary Angioedema (HAE) Subjects	Cardiovascular Disease	Drug	38	0		Multiple	Randomized, Parallel Assignment	Industry	2014
Effect of Eleclazine on Shortening of the QT Interval, Safety, and Tolerability in Adults With Long QT Syndrome Type 3	Cardiovascular Disease	Drug	41	1	American Indian or Alaska Native	Multiple	Single Group Assignment	Industry	2014
Efficacy and Safety of Insulin Glargine/ Lixisenatide Fixed Ratio Combination Compared to Insulin Glargine Alone and Lixisenatide Alone on Top of Metformin in Patients With T2DM	Diabetes	Drug	1170	0		Multiple	Randomized, Parallel Assignment	Industry	2014
Efficacy and Safety of Riociguat in Patients With Symptomatic Pulmonary Hypertension (PH) Associated With Idiopathic Interstitial Pneumonias (IIP)	Respiratory Disease	Drug	147		Race and Ethnicity Not Collected	Multiple	Randomized, Parallel Assignment	Industry	2014
Efficacy and Safety of Semaglutide Once-weekly Versus Placebo as add-on to Basal Insulin Alone or Basal Insulin in Combination With Metformin in Subjects With Type 2 Diabetes	Diabetes	Drug	397	0		Multiple	Randomized, Parallel Assignment	Industry	2014

Efficacy and Safety of the Insulin Glargine/Lixisenatide Fixed Ratio Combination Versus Insulin Glargine in Patients With Type 2 Diabetes	Diabetes	Drug	736	0		Multiple	Randomized, Parallel Assignment	Industry	2014
Efficacy and Safety Study of Benralizumab to Reduce OCS Use in Patients With Uncontrolled Asthma on High Dose Inhaled Corticosteroid Plus LABA and Chronic OCS Therapy	Respiratory Disease	Biological	220		Race and Ethnicity Not Collected	Multiple	Randomized, Parallel Assignment	Industry	2014
Efficacy and Safety Study of Bimatoprost Sustained-Release (SR) in Participants With Open-angle Glaucoma or Ocular Hypertension	Hypertension	Drug	594	0		Multiple	Randomized, Parallel Assignment	Industry	2014
EINSTEIN Junior: Oral Rivaroxaban in Children With Venous Thrombosis	Cardiovascular Disease	Drug	500	0		Multiple	Randomized, Parallel Assignment	Industry	2014
Estradiol Vaginal Softgel Capsules in Treating Symptoms of Vulvar and Vaginal Atrophy in Postmenopausal Women	Mental Illness	Drug	764	0		Multiple	Randomized, Parallel Assignment	Industry	2014
Evaluate the Maintenance of Efficacy of SPD489 in Adults Aged 18-55 Years With Moderate to Severe Binge Eating Disorder	Mental Illness	Drug	418	0		Multiple	Randomized, Parallel Assignment	Industry	2014
Evaluation of Umeclidinium Bromide in Combination With Fluticasone Furoate in COPD Subjects With an Asthmatic Component	Respiratory Disease	Drug	338	0		Multiple	Randomized, Parallel Assignment	Industry	2014
Investigation of Safety, Tolerability, Pharmacokinetics, Pharmacodynamics, and Clinical Efficacy of Oral Danirixin in Symptomatic COPD Subjects With Mild to Moderate Airflow Limitation at Risk for Exacerbations	Respiratory Disease	Drug	102	0		Multiple	Single Group Assignment	Industry	2014
Long Term Safety of Immediate-release Tolvaptan in Subjects With Autosomal Dominant Polycystic Kidney Disease	Kidney Disease	Drug	1803	6	American Indian or Alaska Native, Native Hawaiian or Other Pacific Islander	Multiple	Single Group Assignment	Industry	2014
Lung-MAP: Taselisib as Therapy in Treating Patients With Stage IV Squamous Cell Lung Cancer and Positive Biomarker Matches	Respiratory Disease	Other	26	1	American Indian or Alaska Native	Multiple	Single Group Assignment	NIH	2014
Pharmacokinetic Single Dose Trial of Empagliflozin in Children and Adolescents With Type 2 Diabetes Mellitus	Diabetes	Drug	27	3	American Indian or Alaska Native	Multiple	Randomized, Parallel Assignment	Industry	2014
Safety and Efficacy Study of Abraxane as Maintenance Treatment After Abraxane Plus Carboplatin in 1st Line Stage IIIB / IV Squamous Cell Non-small Cell Lung Cancer	Respiratory Disease	Drug	427	1	American Indian or Alaska Native	Multiple	Randomized, Crossover Assignment	Industry	2014
Safety of Dabigatran Etexilate in Blood Clot Prevention in Children	Cardiovascular Disease	Drug	214	0		Multiple	Single Group Assignment	Industry	2014
Safety, Pharmacokinetics and Preliminary Efficacy Study of CFZ533 in Patients With PrimarySjögren's Syndrome	Arthritis	Drug	69	0		Multiple	Randomized, Parallel Assignment	Industry	2014

Study of FX006 vs Normal Saline in Patients With Osteoarthritis of the Knee	Arthritis	Drug	310	0		Multiple	Randomized, Parallel Assignment	Industry	2014
Efficacy and Safety of Alirocumab Versus Placebo on Top of Maximally Tolerated Lipid Lowering Therapy in Patients With Hypercholesterolemia Who Have Type 1 or Type 2 Diabetes and Are Treated With Insulin (ODYSSEY DM - Insulin)	Cardiovascular Disease	Drug	517	0		Multiple	Randomized, Parallel Assignment	Industry	2015
ELUVIAâ„¢ Drug-eluting Stent Versus ZilverÂ® PTXÂ® Stent	Cardiovascular Disease	Device	524	5	American Indian or Alaska Native, Native Hawaiian or Other Pacific Islander	Multiple	Randomized, Parallel Assignment	Industry	2015
Emdogain Minimally Invasive Surgical Technique	Dental Disease	Procedure	51	0		Multiple	Randomized, Parallel Assignment	Research Institute	2015
Extension Study of Ataluren in Participants With Nonsense Mutation Cystic Fibrosis	Respiratory Disease	Drug	246	0		Multiple	Single Group Assignment	Industry	2015
Pharmacokinetics of Eleclazine in Adults With Normal and Impaired Hepatic Function	Cardiovascular Disease	Drug	49	0		Multiple	Non-randomized, Parallel Assignment	Industry	2015
Safety and Effectiveness of the Orsiro Sirolimus Eluting Coronary Stent System in Subjects With Coronary Artery Lesions	Cardiovascular Disease	Device	1334	3	Native Hawaiian or other Pacific Islander	Multiple	Randomized, Parallel Assignment	Industry	2015
Safety and Efficacy Study of NabÂ®-Paclitaxel With CC-486 or NabÂ®-Paclitaxel With Durvalumab, and NabÂ®-Paclitaxel Monotherapy as Second/Third-line Treatment for Advanced Non-small Cell Lung Cancer	Respiratory Disease	Drug	240	0		Multiple	Non-randomized, Parallel Assignment	Industry	2015
Study Comparing Daratumumab, Lenalidomide, and Dexamethasone With Lenalidomide and Dexamethasone in Participants With Previously Untreated Multiple Myeloma	Cardiovascular Disease	Drug	737	1	Native Hawaiian or Other Pacific Islander	Multiple	Randomized, Parallel Assignment	Research Institute	2015
Study to Assess the Efficacy and Safety of PT003, PT005, and PT001 in Subjects With Moderate to Very Severe COPD	Respiratory Disease	Drug	1756	1	American Indian or Alaska Native	Multiple	Randomized, Parallel Assignment	Industry	2015
Study to Evaluate Maintenance of Sustained Remission of axSpA With CZP Compared to Placebo	Arthritis	Biological	736	2	American Indian or Alaska Native	Multiple	Randomized, Parallel Assignment	Industry	2015
Tai Chi for stress and cardiovascular function	Cardiovascular Disease	Other	120	0		Multiple	Randomized, Parallel Assignment	University	2015
A Study to Evaluate the Onset of Effect and Time Course of Change in Lung Function With Benralizumab in Severe, Uncontrolled Asthma Patients With Eosinophilic Inflammation	Respiratory Disease	Drug	233	0		Multiple	Randomized, Parallel Assignment	Industry	2016

An 8-Week Dose-Finding Study to Evaluate the Efficacy and Safety of Alirocumab in Children and Adolescents With Heterozygous Familial Hypercholesterolemia	Cardiovascular Disease	Drug	42	0		Multiple	Non-randomized, Sequential Assignment	Industry	2016
Effect of Lu AF35700 in Patients With Treatment-resistant Schizophrenia	Mental Illness	Drug	1098	4	American Indian or Alaska Native, Native Hawaiian or Other Pacific Islander	Multiple	Randomized, Parallel Assignment	Industry	2016
Efficacy and Safety of Faster-acting Insulin Aspart Compared to NovoRapidÂ® Both in Combination With Insulin Degludec in Adults With Type 1 Diabetes	Diabetes	Drug	1108	2	American Indian or Alaska Native	Multiple	Randomized, Parallel Assignment	Industry	2016
Efficacy and Safety of Gemcabene in Patients With Homozygous Familial Hypercholesterolemia on Stable, Lipid-Lowering Therapy (COBALT-1)	Cardiovascular Disease	Drug	8	0		Multiple	Single Group Assignment	Industry	2016
Evaluation of Evolocumab (AMG 145) Efficacy in Diabetic Adults With Hypercholesterolemia/Mixed Dyslipidemia	Diabetes	Biological	424	17	American Indian or Alaska Native, Native Hawaiian or Other Pacific Islander	Multiple	Randomized, Parallel Assignment	Industry	2016
Evaluation of Immunogenicity and Safety of a Booster Dose of Infanrix Hexaâ„¢ in Healthy Infants Born to Mothers Vaccinated With Boostrixâ„¢ During Pregnancy or Immediately Post-delivery	Respiratory Disease	Biological	551	0		Multiple	Non-randomized, Parallel Assignment	Industry	2016
Evaluation of Long-Term Safety and Tolerability of ETC-1002 in High-Risk Patients With Hyperlipidemia and High CV Risk (CLEAR Harmony)	Cardiovascular Disease	Drug	2230	5	American Indian or Alaska Native, Native Hawaiian or Other Pacific Islander	Multiple	Randomized, Parallel Assignment	Industry	2016
Long-term Safety and Efficacy Study of DX-2930 (SHP643) to Prevent Acute Angioedema Attacks in Patients With Type I and Type II HAE	Cardiovascular Disease	Drug	212	0		Multiple	Single Group Assignment	Industry	2016
Mechanistic Study of GSK3196165 Plus Methotrexate (MTX) in Subjects With Active Rheumatoid Arthritis	Arthritis	Drug	39	0		Multiple	Randomized, Parallel Assignment	Industry	2016
Safety and Efficacy Study of a Protease Activated Receptor-4 Antagonist Being Tested to Reduce the Chances of Having Additional Strokes or "Mini Strokes"	Cardiovascular Disease	Drug	15	0		Multiple	Randomized, Parallel Assignment	Industry	2016
Safety and Efficacy Study of GDC-0853 Compared With Placebo and Adalimumab in Participants With Rheumatoid Arthritis (RA)	Arthritis	Drug	578	51	American Indian or Alaska Native	Multiple	Randomized, Parallel Assignment	Industry	2016

Research Study Comparing a New Medicine "Fast-acting Insulin Aspart" to Another Already Available Medicine "NovoRapid"/"NovoLog" in People With Type 2 Diabetes	Diabetes	Drug	1264	7	Native Hawaiian or Other Pacific Islander	Multiple	Randomized, Parallel Assignment	Industry	2017
Safety and Efficacy of G-Pen Compared to Lilly Glucagon for Hypoglycemia Rescue in Adult Type 1 Diabetics	Diabetes	Drug	80	0		Multiple	Randomized, Crossover Assignment	Industry	2017
Safety, Pharmacokinetics and Efficacy Study of QCC374 in PAH Patients	Respiratory Disease	Drug	8	0		Multiple	Randomized, Parallel Assignment	Industry	2017
Study of LLG783 in Patients With Peripheral Artery Disease (PAD) and Intermittent Claudication	Cardiovascular Disease	Drug	46	0		Multiple	Randomized, Parallel Assignment	Industry	2017
G-Pen Compared to Glucagen Hypokit for Severe Hypoglycemia Rescue in Adults With Type 1 Diabetes	Diabetes	Drug	132	1	Native Hawaiian or Other Pacific Islander	Multiple	Randomized, Crossover Assignment	Industry	2018
Safety and Efficacy of EMA401 in Patients With Painful Diabetic Neuropathy (PDN)	Diabetes	Drug	142	0		Multiple	Randomized, Parallel Assignment	Industry	2018
A randomized controlled trial to study the effect of a dietary supplement - a novel marine extract (BioLex (Registered Trademark)) - for the treatment of osteoarthritis.	Arthritis	Other	80	5	Maori and Pacific peoples	New Zealand	Randomized, Parallel Assignment	Industry	2011
Can the companion robot Paro improve quality of life in a residential care facility: A randomised controlled trial	Mental Illness	Device	40		Race and Ethnicity Not Collected	New Zealand	Randomized, Parallel Assignment	University	2012
Bronchiolitis / Paracetamol in infancy: a feasibility study	Respiratory Disease	Other	40	40	Maori and Pacific	New Zealand	Randomized, Parallel Assignment	HRC	2013
Comparative assessment of the absorption of a generic formulation of desvenlafaxine extended release tablet against the innovator desvenlafaxine extended release tablet conducted under fasting conditions and at steady state in healthy male and female volunteers	Mental Illness	Drug	26		Not Available	New Zealand	Randomized, Crossover Assignment	Industry	2013
The BROAD study. A trial using the whole-foods, plant-based diet in a community programme for people with obesity, or overweight with ischaemic heart disease or diabetes	Cardiovascular Disease	Behavioral	65	8	Maori	New Zealand	Randomized, Parallel Assignment	Industry	2014
Comparative assessment of the absorption of a generic formulation of metformin XR tablet against the innovator metformin SR prolonged release tablet conducted under fed conditions in healthy male and female volunteers	Diabetes	Drug	24		Not Available	New Zealand	Randomized	Industry	2015
In healthy adults, is provision of nutritional support effective for following low dietary sodium intake?	Cardiovascular Disease	Behavioral	11		Not Available	New Zealand	Non-randomized	University	2015
Influence of habitual dietary fibre intake on the responsiveness of the gut microbiota to a prebiotic	Cardiovascular Disease	Behavioral	45	3	Maori	New Zealand	Randomized, Parallel Assignment	Research Institute	2015
Using robots to reduce hospitalisations in patients with Chronic Obstructive Pulmonary Disease (COPD).	Respiratory Disease	Other	60	23	Maori and Pacific Islander	New Zealand	Randomized, Parallel Assignment	HRC	2015

A study designed to test a new oxygen delivery device (NHFO2) in patients with long term respiratory disease who require home oxygen.	Respiratory Disease	Device	12		Not Available	New Zealand	Single Group Assignment	Industry	2017
Comfort comparison of two bi-level devices in patients with chronic respiratory diseases.	Respiratory Disease	Device	30	6	Maori and Pacific	New Zealand	Randomized, Crossover Assignment	Industry	2017
Effect of prolonged fasting on blood sugars in overweight/obese volunteers with type 2 diabetes	Diabetes	Behavioral	16		Not Available	New Zealand	Randomized, Crossover Assignment	University	2017
Exergaming for people with knee osteoarthritis: a feasibility study	Arthritis	Rehabilitation	23	0		New Zealand	Randomized, Parallel Assignment	Industry	2017
Food 4 Health: using probiotics and prebiotic cereals to reduce blood sugar levels in adults at risk of developing type 2 diabetes<br>He Oranga Kai	Diabetes	Behavioral	153		Not Available	New Zealand	Randomized, Factorial Assignment	HRC	2017
A placebo-controlled, single-blind, crossover study to assess the effects of New Zealand pine bark extract (Enzogenol®) on glycaemic responses in healthy participants	Diabetes	Other	25		Race and Ethnicity Not Collected	New Zealand	Non-randomized, Crossover Assignment	Industry	2018
mobile Pulmonary Rehabilitation - Determining the feasibility and acceptability of an adaptive mobile Pulmonary Rehabilitation (PR) programme	Respiratory Disease	Behavioral	30	11	Maori and Pacific	New Zealand	Non-randomized	Research Institute	2019
6-week Study Treatment to Evaluate the Safety and Effectiveness of AZD2066 in Patients With Major Depressive Disorder	Mental Illness	Drug	249		Race and Ethnicity Not Collected	US	Randomized, Parallel Assignment	Industry	2010
A 6-week Study in Asthmatic Children Aged 6 to <12 Yrs Comparing Budesonide pMDI 160ug Twice Daily With Placebo	Respiratory Disease	Drug	304	1	Native Hawaiian or Other Pacific Islander	US	Randomized, Parallel Assignment	Industry	2010
A Study in Participants With Type 2 Diabetes Mellitus (AWARD-4)	Diabetes	Drug	884	47	American Indian or Alaska Native, Native Hawaiian or Other Pacific Islander	US	Randomized, Parallel Assignment	Industry	2010
A Study of Carfilzomib vs Best Supportive Care in Subjects With Relapsed and Refractory Multiple Myeloma	Cardiovascular Disease	Drug	315	0		US	Randomized, Parallel Assignment	Industry	2010
A Study of First-Line Ambrisentan and Tadalafil Combination Therapy in Subjects With Pulmonary Arterial Hypertension (PAH)	Respiratory Disease	Drug	610	5	American Indian or Alaska Native, Native Hawaiian or Other Pacific Islander	US	Randomized, Parallel Assignment	Industry	2010

A Study of LY2216684 and Digoxin in Healthy Subjects	Mental Illness	Drug	30	0		US	Non-randomized, Single Group Assignment	Industry	2010
A Study to Evaluate the Effects of Milnacipran on Pain Processing and Functional MRI in Patients With Fibromyalgia	Arthritis	Drug	22		Race and Ethnicity Not Collected	US	Randomized, Crossover Assignment	University	2010
ADAPT: Addressing Depression and Pain Together	Mental Illness	Drug	139		Race and Ethnicity Not Collected	US	Randomized, Parallel Assignment	University	2010
Alefacept and Allogeneic Hematopoietic Stem Cell Transplantation	Cardiovascular Disease	Drug	3		Race and Ethnicity Not Collected	US	Single Group Assignment	University	2010
Aminophylline to Prevent Acute Kidney Injury in Children After Cardiac Surgery	Kidney Disease	Drug	144		Race and Ethnicity Not Collected	US	Randomized, Parallel Assignment	University	2010
An Advanced Echocardiographic Evaluation of Nebivolol	Hypertension	Drug	2		Race and Ethnicity Not Collected	US	Single Group Assignment	Industry	2010
Aqueous Humor Dynamics and Brimonidine	Hypertension	Drug	35	1	Native American	US	Randomized, Crossover Assignment	University	2010
Ascorbic Acid (Vitamin C) Infusion in Human Sepsis	Respiratory Disease	Drug	24	0		US	Single Group Assignment	University	2010
Calorie Restriction and Changes in Body Composition, Disease, Function, and Quality of Life in Older Adults	Cardiovascular Disease	Behavioral	167	0		US	Randomized, Factorial Assignment	University	2010
Carvedilol for Psychostimulant Dependence	Mental Illness	Drug	32	0		US	Randomized, Parallel Assignment	NIH	2010
Clinical Trial of Pioglitazone for Prevention of Cardiac Allograft Vasculopathy After Heart Transplantation	Cardiovascular Disease	Drug	18		Race and Ethnicity Not Collected	US	Randomized, Parallel Assignment	University	2010
Comparing Self Monitored Blood Glucose (SMBG) to Continuous Glucose Monitoring (CGM) in Type 2 Diabetes	Diabetes	Device	124		Race and Ethnicity Not Collected	US	Randomized, Parallel Assignment	Research Institute	2010
Comparison of Couple-Based PTSD Treatment and Couple-Based PTSD Education	Mental Illness	Behavioral	138	4	American Indian or Alaska Native, Native Hawaiian or Other Pacific Islander	US	Randomized, Parallel Assignment	Research Institute	2010
Comparison of Intravenous Adenosine Infusion With Regadenoson Bolus for Inducing Maximal Coronary Hyperemia	Cardiovascular Disease	Drug	46	0		US	Single Group Assignment	University	2010
Comparison of Surgical Time and Efficiency of Total Knee Arthroplasty	Arthritis	Other	30		Race and Ethnicity Not Collected	US	Non-Randomized,	Industry	2010

							Parallel Assignment		
Corticotropin-Releasing Hormone Receptor 1 (CRH1) Antagonism in Anxious Alcoholics^	Mental Illness	Drug	70	0		US	Randomized, Parallel Assignment	NIH	2010
Drum-Assisted Therapy for Native Americans	Mental Illness	Other	10	10	Native Americans	US	Randomized, Parallel Assignment	University	2010
Effects of Mindfulness-Based Cognitive-Behavioral Conjoint Therapy on PTSD and Relationship Function	Mental Illness	Behavioral	92	0		US	Randomized, Parallel Assignment	Research Institute	2010
Efficacy and Safety of Empagliflozin (BI 10773) in Type 2 Diabetes Patients on a Background of Pioglitazone Alone or With Metformin	Diabetes	Drug	499	2	American Indian or Alaska Native	US	Randomized, Parallel Assignment	Industry	2010
Epoprostenol for Injection in Patients With Pulmonary Arterial Hypertension	Respiratory Disease	Drug	30		Race and Ethnicity Not Collected	US	Randomized, Parallel Assignment	Industry	2010
Epoprostenol for Injection in Pulmonary Arterial Hypertension - Extension of AC-066A401	Respiratory Disease	Drug	2		Race and Ethnicity Not Collected	US	Non-randomized, Parallel Assignment	Industry	2010
Evaluation of Therapeutic Plasma Exchange (TPE) Procedure Using the AMICUS Device	Kidney Disease	Device	37		Race and Ethnicity Not Collected	US	Randomized, Crossover Assignment	Industry	2010
Event Marker Ingested To Trigger Event Recorder 3.0 Psychiatry Study	Mental Illness	Other	28	1	Native American	US	Single Group Assignment	Hospital	2010
Extended Follow-up of Patients With Macular Edema Due to Retinal Vein Occlusion	Cardiovascular Disease	Drug	66	0		US	Single Group Assignment	University	2010
Identifying Treatments to Motivate Smokers to Quit	Mental Illness	Drug	517	0		US	Randomized, Factorial Assignment	University	2010
Impact of Exenatide on Cardiovascular Exercise Performance in Type 2 Diabetes	Diabetes	Drug	23		Race and Ethnicity Not Collected	US	Randomized, Parallel Assignment	University	2010
Intravitreal Ranibizumab for Vitreous Hemorrhage Due to Proliferative Diabetic Retinopathy (N)	Diabetes	Drug	261	5	American Indian or Alaska Native, Native Hawaiian or Other Pacific Islander	US	Randomized, Parallel Assignment	NIH	2010
Investigation of Mifepristone (RU486) on Stress Sensitivity and Relapse Prevention in Cocaine Dependent Patients	Mental Illness	Drug	58	0		US	Randomized, Parallel Assignment	Research Institute	2010
Investigation of the Efficacy of Antibiotics on Pulmonary Sarcoidosis	Respiratory Disease	Drug	15	0		US	Single Group Assignment	University	2010

Investigator Sponsored Trial of Polypoidal Choroidal Vasculopathy (PCV) Evaluation Assessing High-Dose Ranibizumab Prospectively (PEARL2)	Cardiovascular Disease	Drug	24	0		US	Non-randomized, Parallel Assignment	Industry	2010
Nebivolol Effect on Nitric Oxide Levels, Blood Pressure, and Renal Function in Kidney Transplant Patients	Hypertension	Drug	32	0		US	Randomized, Parallel Assignment	University	2010
Ocular Surface Tolerability Study of Prostaglandin Analogues in Patients With Open-Angle Glaucoma or Ocular Hypertension	Hypertension	Drug	164		Race and Ethnicity Not Collected	US	Randomized, Parallel Assignment	Industry	2010
Pilot of Acupuncture to Improve Quality of Life in Veterans With TBI and PTSD	Mental Illness	Drug	34		Race and Ethnicity Not Collected	US	Single Group Assignment	Research Institute	2010
Randomized Control Trial of Fluid Therapy for Pediatric Diabetic Ketoacidosis	Diabetes	Drug	1389	0		US	Randomized, Factorial Assignment	NIH	2010
Ranolazine and Pulmonary Hypertension	Respiratory Disease	Drug	11	0		US	Single Group Assignment	University	2010
Safety and Efficacy of AGN-210961 Ophthalmic Solution Compared With Bimatoprost Ophthalmic Solution in Patients With Glaucoma or Ocular Hypertension	Hypertension	Drug	163		Race and Ethnicity Not Collected	US	Randomized, Parallel Assignment	Industry	2010
Safety and Efficacy of Vortioxetine (Lu AA21004) in Adults With Major Depressive Disorder	Mental Illness	Drug	614	1	American Indian or Alaska Native	US	Randomized, Parallel Assignment	Industry	2010
Safety and Efficacy Study of Vortioxetine (Lu AA21004) in Adults With Major Depressive Disorder	Mental Illness	Drug	469	3	American Indian or Alaska Native	US	Randomized, Parallel Assignment	Industry	2010
Safety and Pharmacodynamics Study of AGN-207281 Ophthalmic Solutions Compared With Timolol Ophthalmic Solution or Placebo in Patients With Ocular Hypertension or Primary Open-Angle Glaucoma	Hypertension	Drug	50		Race and Ethnicity Not Collected	US	Randomized, Parallel Assignment	Industry	2010
Safety Study of Escitalopram in Children 7 to 11 Years of Age With Major Depressive Disorder	Mental Illness	Drug	162	2	American Indian or Alaska Native, Native Hawaiian or Other Pacific Islander	US	Single Group Assignment	Industry	2010
School-Based Preventive Asthma Care Technology: A Trial Using a Novel Technology to Improve Adherence	Respiratory Disease	Other	99	0		US	Randomized, Parallel Assignment	NIH	2010
Stereotactic Body Radiation Therapy (SBRT) in Metastatic Non-small Cell Lung Cancer	Respiratory Disease	Radiation	29	0		US	Single Group Assignment	University	2010
Sunitinib Malate in Treating Patients With Previously Untreated Metastatic Kidney Cancer	Kidney Disease	Drug	37	0		US	Single Group Assignment	Industry	2010
Systolic Blood Pressure Intervention Trial	Hypertension	Drug	9361	0		US	Randomized, Parallel Assignment	NIH	2010

Testosterone Replacement in Men With Diabetes and Obesity	Diabetes	Drug	144		Race and Ethnicity Not Collected	US	Randomized, Parallel Assignment	NIH	2010
Tetrahydrobiopterin Treatment in Children With Idiopathic Cognitive Developmental Disorders	Mental Illness	Drug	10		Race and Ethnicity Not Collected	US	Single Group Assignment	University	2010
The Influence of Smoking Status on Prasugrel and Clopidogrel Treated Subjects Taking Aspirin and Having Stable Coronary Artery Disease	Cardiovascular Disease	Drug	110	2	American Indian or Alaska Native	US	Randomized, Crossover Assignment	Industry	2010
The Intravascular Cooling in the Treatment of Stroke 2/3 Trial	Cardiovascular Disease	Device	120		Race and Ethnicity Not Collected	US	Randomized, Factorial Assignment	NIH	2010
Treatment of Patients Undergoing Primary Unilateral Elective Total Knee or Hip Replacement With Dabigatran Etexilate	Kidney Disease	Drug	112		Race and Ethnicity Not Collected	US	Single Group Assignment	Industry	2010
Trial of Citicoline Therapy in Patients With Mania or Hypomania and Cocaine Abuse/Dependence	Mental Illness	Drug	44		Race and Ethnicity Not Collected	US	Randomized, Parallel Assignment	University	2010
Trial of ID-Specific Donor Vaccinated Lymphocyte Infusion for Patients With Myeloma Relapsing or Failing to Achieve a Complete Remission After an Allogenic Transplant	Cardiovascular Disease	Biological	2	0		US	Single Group Assignment	Industry	2010
Using Computers to Assist in the Diagnosis and Treatment of Attention-deficit/Hyperactivity Disorder (ADHD)	Mental Illness	Other	84	0		US	Randomized, Parallel Assignment	NIH	2010
6-month Safety and Benefit Study of ADVAIR in Children 4-11 Years Old	Respiratory Disease	Drug	6250	268	American Indian or Alaska Native, Native Hawaiian or Other Pacific Islander	US	Randomized, Parallel Assignment	Industry	2011
A 12 Week Study to Assess Changes in Joint Inflammation Using Ultrasonography in Patients With Rheumatoid Arthritis (RA)	Arthritis	Biological	3		Race and Ethnicity Not Collected	US	Single Group Assignment	Industry	2011
A 24-week Arterial Stiffness Study With Fluticasone Furoate/Vilanterol in COPD	Respiratory Disease	Drug	430	0		US	Randomized, Parallel Assignment	Industry	2011
A Multiple Dose Study Of PF-04620110 In Type 2 Diabetes Patients	Diabetes	Drug	48		Race and Ethnicity Not Collected	US	Randomized, Parallel Assignment	Industry	2011
A Phase 3 Multi-center Study to Assess PET Imaging of Flurpiridaz F 18 Injection in Patients With CAD.	Cardiovascular Disease	Drug	795	6	American Indian or Alaska Native, Native Hawaiian or Other Pacific Islander	US	Single Group Assignment	Industry	2011

A Study in Prevention of Re-emergence of Depression Symptoms	Mental Illness	Drug	1249	50	American Indian or Alaska Native	US	Randomized, Parallel Assignment	Industry	2011
A Study of Cariprazine in the Prevention of Relapse of Symptoms in Participants With Schizophrenia	Mental Illness	Drug	765	1	Native Hawaiian or other Pacific Islander	US	Randomized, Parallel Assignment	Industry	2011
A Study of Hypertonic Saline for Infants Hospitalized With Bronchiolitis	Respiratory Disease	Drug	227	0		US	Randomized, Parallel Assignment	Industry	2011
A Study of Intravenous Zanamivir Versus Oral Oseltamivir in Adults and Adolescents Hospitalized With Influenza	Respiratory Disease	Drug	626	12	American Indian or Alaska Native, Native Hawaiian or Other Pacific Islander	US	Randomized, Parallel Assignment	Industry	2011
A Study of LY2127399 in Participants With Systemic Lupus Erythematosus	Arthritis	Drug	1124	97	American Indian or Alaska Native, Native Hawaiian or Other Pacific Islander	US	Randomized, Parallel Assignment	Industry	2011
A Study of LY2216684 in Healthy Females	Mental Illness	Drug	20	0		US	Randomized, Crossover Assignment	Industry	2011
A Study of RoActemra/Actemra (Tocilizumab) Versus Adalimumab in Combination With Methotrexate (MTX) in Patients With Moderate to Severe Active Rheumatoid Arthritis And an Inadequate Response to Treatment With Only One Tumor Necrosis Factor (TNF)-Inhibitor	Arthritis	Drug	96		Race and Ethnicity Not Collected	US	Randomized, Parallel Assignment	Industry	2011
A Study of the Effect of MK-8457 on Blood Pressure in Hypertensive Participants (MK-8457-004-AM1)	Hypertension	Drug	31		Race and Ethnicity Not Collected	US	Randomized, Crossover Assignment	Industry	2011
A Study of the Safety and Efficacy of Bimatoprost Ophthalmic Solution in Paediatric Patients With Glaucoma	Hypertension	Drug	6		Race and Ethnicity Not Collected	US	Randomized, Parallel Assignment	Industry	2011
Addiction Housing Case Management for Homeless Veterans	Mental Illness	Behavioral	181	8	Native American, Asian/Pacific Islander	US	Randomized, Parallel Assignment	Industry	2011
Adjunctive Minocycline in Clozapine Treated Schizophrenia Patients	Mental Illness	Drug	52	0		US	Randomized, Parallel Assignment	NIH	2011
Antiplatelet Therapy Guided by Thrombelastography in Patients With Acute Coronary Syndromes (TEGCOR Study)	Cardiovascular Disease	Drug	67	0		US	Randomized, Parallel Assignment	University	2011

Asenapine in the Treatment of Older Adults With Bipolar Disorder	Mental Illness	Drug	15	0		US	Single Group Assignment	University	2011
Beating the Blues for Your Heart	Mental Illness	Behavioral	29		Race and Ethnicity Not Collected	US	Randomized, Parallel Assignment	University	2011
Blacks and Exacerbations on Long Acting Beta Agonists (LABA) vs. Tiotropium (BELT)	Respiratory Disease	Drug	1070	0		US	Randomized, Parallel Assignment	Hospital	2011
Blood Pressure Telemonitoring and Goal Blood Pressure in Diabetes	Diabetes	Device	50		Race and Ethnicity Not Collected	US	Randomized, Parallel Assignment	Hospital	2011
Bronchoscopic Intratumoral Chemotherapy for Small Cell Lung Cancer (SCLC)	Respiratory Disease	Drug	4		Race and Ethnicity Not Collected	US	Single Group Assignment	University	2011
Bupropion for Smoking Cessation During Pregnancy	Mental Illness	Drug	65	0		US	Randomized, Parallel Assignment	NIH	2011
Cardiovascular Intervention Improvement Telemedicine Study	Cardiovascular Disease	Behavioral	428	0		US	Randomized, Parallel Assignment	Research Institute	2011
Carotid Artery Stenting Outcomes in the Standard Risk Population for Carotid Endarterectomy	Cardiovascular Disease	Device	1203	11	American Indian or Alaska Native, Native Hawaiian or Other Pacific Islander	US	Single Group Assignment	Industry	2011
Clinical Pharmacology of Aliskiren in Combination With Cyclosporine in Cardiac Transplantation	Hypertension	Drug	1		Race and Ethnicity Not Collected	US	Single Group Assignment	University	2011
Combination Immunotherapy and Autologous Stem Cell Transplantation for Myeloma	Cardiovascular Disease	Biological	27	0		US	Single Group Assignment	University	2011
Comparison of 2 Beta Blocker Drugs on Peripheral Arterial Disease in Patients With High Blood Pressure	Cardiovascular Disease	Drug	17	0		US	Randomized, Parallel Assignment	University	2011
Computerized Cognitive Behavioral Therapy for Childhood Anxiety in Community Health Centers	Mental Illness	Behavioral	100	3	Asian/Pacific Islander	US	Randomized, Parallel Assignment	University	2011
Cortisol Suppression and Startle Responses in Posttraumatic Stress Disorder (PTSD)	Mental Illness	Drug	165	0		US	Randomized, Crossover Assignment	NIH	2011
Diagnostic Device Risk Management of Atrial Fibrillation and Heart Failure	Cardiovascular Disease	Device	24	0		US	Randomized, Parallel Assignment	Industry	2011
Dipeptidyl Peptidase-4 Inhibitor (Sitagliptin) Therapy in the Inpatients With Type 2 Diabetes	Diabetes	Drug	90		Race and Ethnicity Not Collected	US	Randomized, Parallel Assignment	University	2011

Dosing Study of Ranibizumab for Diabetic Retinal and Macular Edema	Diabetes	Drug	20		Race and Ethnicity Not Collected	US	Randomized, Parallel Assignment	Industry	2011
Early Intervention in Cystic Fibrosis Exacerbation	Respiratory Disease	Device	267	0		US	Randomized, Parallel Assignment	NIH	2011
Effect of Ketoconazole on Breathlessness	Respiratory Disease	Drug	20		Race and Ethnicity Not Collected	US	Randomized, Crossover Assignment	Hospital	2011
Effects of Febuxostat on Adipokines and Kidney Disease in Diabetic Chronic Kidney Disease	Kidney Disease	Drug	80	0		US	Randomized, Parallel Assignment	University	2011
Efficacy and Safety of Colchicine for the Prevention of Gout Flares During the Initiation of Allopurinol	Arthritis	Drug	82		Race and Ethnicity Not Collected	US	Randomized, Parallel Assignment	Industry	2011
Efficacy and Tolerability of Riluzole in Treatment Resistant Depression	Mental Illness	Drug	104		Race and Ethnicity Not Collected	US	Randomized, Parallel Assignment	University	2011
Efficacy of an Intravitreal DEX Implant in Retinal Vein Occlusion Following Treatment With Anti-VEGF Injections.	Cardiovascular Disease	Drug	10		Race and Ethnicity Not Collected	US	Randomized, Parallel Assignment	Research Institute	2011
Endothelial Facilitation in Alzheimer's Disease	Mental Illness	Drug	11	0		US	Single Group Assignment	University	2011
Erlotinib Plus Tivantinib (ARQ 197) Versus Single Agent Chemotherapy in Locally Advanced or Metastatic Non-Small Cell Lung Cancer	Respiratory Disease	Drug	96	0		US	Randomized, Parallel Assignment	Industry	2011
Evaluation of Efficacy of Experimental Gel to Foam Dentifrices in Dental Erosion	Dental Disease	Drug	56		Race and Ethnicity Not Collected	US	Randomized, Crossover Assignment	Industry	2011
Feasibility of Delivering a Quitline Based Smoking Cessation Intervention in Cancer Patients	Respiratory Disease	Drug	146	2	American Indian or Alaska Native	US	Randomized, Parallel Assignment	NIH	2011
Galvanic Vestibular Stimulation and Motor Training in Traumatic Brain Injury Survivors	Mental Illness	Device	7	0		US	Randomized, Parallel Assignment	Hospital	2011
Green Tea Lozenges for the Management of Dry Mouth	Arthritis	Dietary Supplement	60		Race and Ethnicity Not Collected	US	Randomized, Parallel Assignment	Industry	2011
Group Physical Therapy for Knee Osteoarthritis	Arthritis	Other	320	0		US	Randomized, Parallel Assignment	Research Institute	2011
How Accurately Does the Diopsys Visual Evoked Potential (VEP) Vision Testing System Detect Glaucoma?	Hypertension	Diagnosis	136	0		US	Randomized, Parallel Assignment	Industry	2011
IL1-TRAP, Rilonacept, in Systemic Sclerosis	Arthritis	Drug	24		Race and Ethnicity Not Collected	US	Randomized, Parallel Assignment	University	2011

Imatinib and Rituximab in Treating Cutaneous Sclerosis in Patients With Chronic Graft-Versus-Host Disease	Arthritis	Drug	72		Race and Ethnicity Not Collected	US	Randomized, Crossover Assignment	NIH	2011
Improving Obesity Self-Care Among Mentally Ill Veterans	Mental Illness	Behavioral	44	2	Native Hawaiian or other Pacific Islander	US	Randomized, Parallel Assignment	Research Institute	2011
Inducing Remission in Type 1 Diabetes With Alefacept	Diabetes	Drug	49	0		US	Randomized, Parallel Assignment	NIH	2011
Intervening in Diabetes With Healthy Eating, Activity, and Linkages To Healthcare - The I-D-HEALTH Study	Diabetes	Behavioral	331	0		US	Randomized, Parallel Assignment	NIH	2011
Life Goals Collaborative Care to Improve Health Outcomes in Mental Disorders	Mental Illness	Behavioral	304	0		US	Randomized, Parallel Assignment	Research Institute	2011
Lower Extremity Splinting to Manage Pain and Sleep Disturbances Associated With HIV/AIDS Related Peripheral Neuropathy	Mental Illness	Device	46	0		US	Randomized, Parallel Assignment	University	2011
Medicinal Nicotine for Preventing Stress Induced Craving and Withdrawal Symptoms	Mental Illness	Drug	98	3	American Indian or Alaska Native	US	Randomized, Crossover Assignment	NIH	2011
Metformin and Transient Hyperglycemia	Diabetes	Drug	4		Race and Ethnicity Not Collected	US	Single Group Assignment	Hospital	2011
Milnacipran in the Treatment of Widespread, Non-Joint Pain in Rheumatoid Arthritis	Arthritis	Drug	49	0		US	Randomized, Crossover Assignment	NIH	2011
Minocycline for Bipolar Depression	Mental Illness	Drug	20	0		US	Single Group Assignment	University	2011
Modulation of Autophagy in Patients With Advanced/Recurrent Non-small Cell Lung Cancer - Phase II	Respiratory Disease	Drug	32	0		US	Non-randomized, Parallel Assignment	NIH	2011
Monoamine Contributions to Neurocircuitry in Eating Disorders	Mental Illness	Drug	88	0		US	Single Group Assignment	NIH	2011
Patient and Provider Interventions for Managing Osteoarthritis in Primary Care	Arthritis	Behavioral	300	3	American Indian or Alaska Native	US	Randomized, Parallel Assignment	Research Institute	2011
PD-1 Alone or With Dendritic Cell/Renal Cell Carcinoma Fusion Cell Vaccine	Kidney Disease	Drug	11		Race and Ethnicity Not Collected	US	Non-randomized, Parallel Assignment	NIH	2011
PET/CT Assessment of Tumor Perfusion in Patients With Renal Cell Carcinoma	Kidney Disease	Drug	14	0		US	Single Group Assignment	University	2011
Pharmacokinetics/Pharmacodynamics of Albiglutide	Diabetes	Biological	283	2	American Indian or Alaska Native,	US	Randomized, Parallel Assignment	Industry	2011

					Native Hawaiian or Other Pacific Islander				
Pilot Study of Using Copeptin to Predict Response to Tolvaptan	Cardiovascular Disease	Drug	21	0		US	Single Group Assignment	University	2011
Pioglitazone in Patients With Mood Disorders	Mental Illness	Drug	37	0		US	Randomized, Parallel Assignment	NIH	2011
Practice Effects and Amyloid Imaging Using 18F-PIB or Flutemetamol PET and FDG-PET	Mental Illness	Drug	27	0		US	Single Group Assignment	University	2011
Preventing Cognitive Decline in African Americans With Mild Cognitive Impairment	Mental Illness	Behavioral	221	0		US	Randomized, Parallel Assignment	University	2011
Prevention of Post-operative Pneumonia (POPP)	Respiratory Disease	Drug	150	0		US	Single Group Assignment	University	2011
PROlonGed ExpoSure Sertraline	Mental Illness	Drug	223	0		US	Randomized, Parallel Assignment	Research Institute	2011
Promoting Adherence to Improve Effectiveness of Cardiovascular Disease Therapies	Cardiovascular Disease	Other	21752	2538	American Indian or Alaska Native, Native Hawaiian or Other Pacific Islander	US	Randomized, Parallel Assignment	Research Institute	2011
Recording for Potential AF Drivers and Patient Specific Atrial Anatomy & Atrial Electrogram Maps	Cardiovascular Disease	Procedure	78		Race and Ethnicity Not Collected	US	Single Group Assignment	University	2011
Reduced Intensity Double Umbilical Cord Blood Transplantation	Cardiovascular Disease	Drug	33	0		US	Single Group Assignment	Hospital	2011
Reducing Length of Stay for Veterans Hospitalized With Pneumonia	Respiratory Disease	Other	129		Race and Ethnicity Not Collected	US	Single Group Assignment	Research Institute	2011
Remote Ischemic Preconditioning Prior to Vascular Surgery	Cardiovascular Disease	Procedure	201		Race and Ethnicity Not Collected	US	Randomized, Parallel Assignment	Research Institute	2011
Results of Tc99m-Maraciclatide Scintigraphy in Subjects With Diabetes Mellitus and Heart Failure With Preserved Left Ventricular Ejection Fraction	Diabetes	Drug	4		Race and Ethnicity Not Collected	US	Single Group Assignment	Industry	2011
Right Ventricular (RV) Pacing in Early Post-operative Continuous Flow Left Ventricular Assist Device (LVAD)	Cardiovascular Disease	Device	3		Race and Ethnicity Not Collected	US	Randomized, Parallel Assignment	University	2011
Safety and Effectiveness Study of Intranasal Insulin Glulisine on Cognitive and Memory in Mild-Mod AD Patients.	Mental Illness	Drug	12		Race and Ethnicity Not Collected	US	Randomized, Crossover Assignment	Research Institute	2011

Safety and Effects of Supplementation With Ergocalciferol on Erythropoietin Dosing in Hemodialysis Patients	Kidney Disease	Dietary Supplement	470	0		US	Randomized, Parallel Assignment	Industry	2011
Safety and Efficacy of BIBF 1120 at High Dose in Idiopathic Pulmonary Fibrosis Patients II	Respiratory Disease	Drug	551	0		US	Randomized, Parallel Assignment	Industry	2011
Safety, Tolerability, and Pharmacokinetics of Iloperidone Depot in Schizophrenic Patients	Mental Illness	Drug	81		Race and Ethnicity Not Collected	US	Randomized, Parallel Assignment	Industry	2011
Social Work Intervention Focused on Transitions	Cardiovascular Disease	Other	181	8	Asian/Pacific Islander and Native American	US	Randomized, Parallel Assignment	NIH	2011
Study of Nasal Insulin to Fight Forgetfulness - Long-acting Insulin Detemir - 120 Days (SL120)	Mental Illness	Drug	36	0		US	Randomized, Parallel Assignment	University	2011
Study of Ranolazine in the Treatment of Pulmonary Hypertension Associated With Diastolic Left Ventricular Dysfunction	Cardiovascular Disease	Drug	10		Race and Ethnicity Not Collected	US	Single Group Assignment	University	2011
Tadalafil for Sarcoidosis Associated Pulmonary Hypertension	Respiratory Disease	Drug	12	0		US	Single Group Assignment	University	2011
Tailored Communication to Reduce Cardiovascular Risk	Cardiovascular Disease	Behavioral	464	0		Us	Randomized, Parallel Assignment	University	2011
T-Cell Depleted Double UCB for Refractory AML	Cardiovascular Disease	Drug	3		Race and Ethnicity Not Collected	US	Single Group Assignment	University	2011
Technology-assisted Case Management in Adults With Type 2 Diabetes	Diabetes	Behavioral	113	0		US	Randomized, Parallel Assignment	University	2011
Telephone Support During Overseas Deployment for Military Spouses	Mental Illness	Behavioral	161	3	American Indian or Alaska Native	US	Randomized, Parallel Assignment	Industry	2011
The Effect of N-Acetyl Cysteine on Cortical Erosion in Early Stage Schizophrenia	Mental Illness	Drug	60	0		US	Randomized, Parallel Assignment	Research Institute	2011
The Health Access and Recovery Peer Program	Cardiovascular Disease	Behavioral	400	0		US	Randomized, Parallel Assignment	NIH	2011
The Safety and Tolerability of Budesonide Foam in Participants With Active Ulcerative Proctitis or Proctosigmoiditis	Respiratory Disease	Drug	114		Race and Ethnicity Not Collected	US	Single Group Assignment	Industry	2011
This Study is Designed to Evaluate PD/PK and Safety of Replagal Manufactured by Two Different Processes.	Cardiovascular Disease	Biological	7		Race and Ethnicity Not Collected	US	Single Group Assignment	Industry	2011
TIV and High Dose TIV in Subjects With Rheumatoid Arthritis	Arthritis	Biological	102	1	Native Hawaiian or other Pacific Islander	US	Randomized, Parallel Assignment	NIH	2011

Treatment for Cannabis Withdrawal and Dependence	Mental Illness	Drug	700		Race and Ethnicity Not Collected	US	Randomized, Parallel Assignment	NIH	2011
Ultrasound Guided Knee Injections in Musculoskeletal Medicine	Arthritis	Procedure	63		Race and Ethnicity Not Collected	US	Randomized, Parallel Assignment	Industry	2011
Use of Santyl in Diabetic Foot Ulcers	Diabetes	Other	55	0		US	Randomized, Parallel Assignment	Industry	2011
Vagal Nerve Stimulation and Glucose Metabolism	Diabetes	Device	7		Race and Ethnicity Not Collected	US	Randomized, Crossover Assignment	NIH	2011
VenaTech Convertible Vena Cava Filter U.S. Multi-Center Clinical Trial	Respiratory Disease	Device	149	0		US	Single Group Assignment	Industry	2011
4 Week Switch Study in Hemodialysis-dependent Subjects With Anemia Associated With Chronic Kidney Disease	Kidney Disease	Drug	80	1	American Indian or Alaska Native	US	Randomized, Parallel Assignment	Industry	2012
5-Fluorouracil Followed by Interferon-alfa-2b in Previously-treated Metastatic Gastrointestinal, Kidney, or Lung Cancer	Respiratory Disease	Drug	18		Race and Ethnicity Not Collected	US	Single Group Assignment	Industry	2012
8-week Randomized, Open-label Study to Evaluate Food Effect on Efficacy and Safety of Oral Aliskiren 300 mg in Patients With Hypertension	Hypertension	Drug	590		Race and Ethnicity Not Collected	US	Randomized, Parallel Assignment	Industry	2012
A 12-week Treatment, Multi-center, Randomized, Double-blind, Parallel-group, Placebo and Active Controlled Study to Assess the Efficacy, Safety, and Tolerability of Indacaterol Maleate / Glycopyrronium Bromide in COPD Patients With Moderate to Severe Airflow Limitation	Respiratory Disease	Drug	1042	0		US	Randomized, Parallel Assignment	Industry	2012
A 3-Month Clinical Trial to Assess the Safety and Efficacy of the OZURDEXÂ® Intraocular Implant in Patients With Diabetes Mellitus	Diabetes	Drug	37		Race and Ethnicity Not Collected	US	Single Group Assignment	Industry	2012
A 6-Week Study Of PF-05175157 In Type 2 Diabetes Mellitus	Diabetes	Drug	19		Race and Ethnicity Not Collected	US	Randomized, Parallel Assignment	Industry	2012
A Long-term Safety Study of ALKS 9072 (Also Known as ALKS 9070)	Mental Illness	Drug	478	2	Native Hawaiian or Other Pacific Islander	US	Single Group Assignment	Industry	2012
A Prospective Study Evaluating the Use of Intraoperative Stroke Volume Variation Via the FloTrac Device to Guide Fluid and Vasopressor Management in Head and Neck Free Flaps	Cardiovascular Disease	Device	94		Race and Ethnicity Not Collected	US	Randomized, Parallel Assignment	University	2012
A Study in Rheumatoid Arthritis (RA) Patients to Compare Two Formulations of Adalimumab for Pharmacokinetic, Pharmacodynamic and Safety	Arthritis	Biological	100		Race and Ethnicity Not Collected	US	Randomized, Parallel Assignment	Industry	2012
A Study of Weekly Carfilzomib in Combination With Dexamethasone for Progressive Multiple Myeloma	Cardiovascular Disease	Drug	116	1	Native Hawaiian or Other Pacific Islander	US	Sequential Assignment	Industry	2012

A Study to Assess Regadenoson Administration Following an Inadequate Exercise Stress Test as Compared to Regadenoson Alone for Myocardial Perfusion Imaging (MPI) Using Single Photon Emission Computed Tomography (SPECT)	Cardiovascular Disease	Drug	1142	0		US	Randomized, Parallel Assignment	Industry	2012
A Study to Compare the Effect of Giving Dulaglutide Using an Auto-injector Versus a Manual Syringe	Diabetes	Biological	50	0		US	Randomized, Crossover Assignment	Industry	2012
A Study to Determine the Effect of Tiotropium + Olodaterol Fixed Dose Combination on Exercise Endurance Time During Constant Work Rate Cycle Ergometry Test in COPD	Respiratory Disease	Device	404		Race and Ethnicity Not Collected	US	Randomized, Parallel Assignment	Industry	2012
ActiV.A.C.+ Compression Therapy Versus Compression Therapy Alone for the Treatment of Chronic Venous Ulcerations	Cardiovascular Disease	Device	2		Race and Ethnicity Not Collected	US	Randomized, Parallel Assignment	University	2012
Actual Human Use of Methotrexate (MTX) Subcutaneously Administered Via the VIBEX MTX Auto-Injector Device	Arthritis	Device	101	0		US	Single Group Assignment	Industry	2012
Aggressive Fever Control With Intravenous Ibuprofen After Non-traumatic Brain Hemorrhage	Cardiovascular Disease	Drug	35	0		US	Randomized, Parallel Assignment	University	2012
Analgesia After Total Hip Arthroplasty: Peri-Articular Injection Versus Epidural Patient Controlled Analgesia (PCA)	Arthritis	Procedure	90	0		US	Randomized, Parallel Assignment	Hospital	2012
Antidepressant Plus Asenapine Versus Antidepressant Plus Placebo for Depression	Mental Illness	Drug	46		Race and Ethnicity Not Collected	US	Randomized, Parallel Assignment	University	2012
Anti-Inflammatory Treatment of Schizophrenia	Mental Illness	Drug	39	0		US	Randomized, Parallel Assignment	University	2012
Assessing the PK and Effect on Glucose and GI Hormone Concentrations of Metformin Delayed-Release in Subjects With T2DM	Diabetes	Drug	24	0		US	Randomized, Crossover Assignment	Industry	2012
Asthma Data Innovation Demonstration Project	Respiratory Disease	Device	95		Race and Ethnicity Not Collected	US	Single Group Assignment	Research Institute	2012
Autologous Cord Blood Stem Cells for Autism	Mental Illness	Biological	30	0		US	Randomized, Crossover Assignment	Research Institute	2012
Bi-Level Positive Airway Ventilation for Acute Chest Syndrome	Respiratory Disease	Device	0		Race and Ethnicity Not Collected	US	Randomized, Parallel Assignment	University	2012
Clinical Efficacy of a Toothpaste in Providing Relief From the Pain of Dentinal Hypersensitivity	Dental Disease	Drug	113			US	Randomized, Parallel Assignment	Industry	2012
Combined Behavioral and Analgesic Trial for Fibromyalgia	Arthritis	Drug and Behavioral	134	2	American Indian or Alaska Native	US	Randomized, Factorial Assignment	University	2012
Compare Technegas Ventilation-Perfusion SPECT and Xenon Ventilation-Perfusion Planar Imaging for Pulmonary Embolism	Respiratory Disease	Drug	18	0		US	Non-randomized,	Industry	2012

							Crossover Assignment		
Comparison of a New Formulation of Insulin Glargine With Lantus in Patients With Type 1 Diabetes Mellitus on Basal Plus Mealtime Insulin	Diabetes	Drug	59		Race and Ethnicity Not Collected	US	Randomized, Parallel Assignment	Industry	2012
Conversations as a Means to Delay the Onset of Alzheimer's Disease	Mental Illness	Behavioral	83		Race and Ethnicity Not Collected	US	Randomized, Parallel Assignment	NIH	2012
Developing New Clinical Management Strategies	Mental Illness	Drug	3		Race and Ethnicity Not Collected	US	Randomized, Parallel Assignment	Research Institute	2012
Donor Atorvastatin Treatment in Preventing Severe Acute GVHD After Nonmyeloablative Peripheral Blood Stem Cell Transplant in Patients With Hematological Malignancies	Cardiovascular Disease	Drug	47	2	American Indian or Alaska Native, Native Hawaiian or Other Pacific Islander	US	Single Group Assignment	NIH	2012
Drug-Eluting Stents vs. Bare Metal Stents In Saphenous Vein Graft Angioplasty	Cardiovascular Disease	Device	597	0		US	Randomized, Parallel Assignment	Research Institute	2012
Early Feasibility Study 2 of Outpatient Control-to-Range - Testing System Efficacy	Diabetes	Device	20		Race and Ethnicity Not Collected	US	Randomized, Crossover Assignment	University	2012
Eclampsia and Posterior Reversible Encephalopathy Syndrome (PRES):	Hypertension	Drug	1		Race and Ethnicity Not Collected	US	Randomized, Parallel Assignment	University	2012
Effects of Eszopiclone on Sleep and Memory in Schizophrenia	Mental Illness	Drug	59	0		US	Randomized, Crossover Assignment	Hospital	2012
Effects of Inhaled Cannabis on Driving Performance	Mental Illness	Drug	98	0		US	Randomized, Crossover Assignment	NIH	2012
Effects of Intranasal Oxytocin on Satiety Signaling in People With Schizophrenia	Mental Illness	Drug	17	0		US	Randomized, Crossover Assignment	University	2012
Effects of Non Contact Low Frequency Ultrasound in Healing Venous Leg Ulcers	Cardiovascular Disease	Device	156	0		US	Randomized, Parallel Assignment	Industry	2012
Efficacy and Safety of Azilsartan Medoxomil Used in Combination With Metformin in Participants With Hypertension and Diabetes	Hypertension	Drug	105	1	Native Hawaiian or Other Pacific Islander	US	Randomized, Parallel Assignment	Industry	2012
Efficacy and Safety of Liraglutide in Combination With Metformin Compared to Metformin Alone, in Children and Adolescents With Type 2 Diabetes	Diabetes	Drug	135	3	American Indian or Alaska Native	US	Randomized, Parallel Assignment	Industry	2012
Efficacy and Safety of Trichuris Suis Ova (TSO) as Compared to Placebo	Cardiovascular Disease	Biological	250	1	American Indian or Alaska Native	US	Randomized, Parallel Assignment	Industry	2012

Efficacy of Inhaled Albuterol SpiromaxÂ® in Subjects With Persistent Asthma With Steady State Pharmacokinetics	Respiratory Disease	Drug	160	2	American Indian or Alaska Native, Pacific Islander	US	Randomized, Parallel Assignment	Industry	2012
Evaluation of a Prototype Diabetes Management System Applied to Insulin Initiation and Titration	Diabetes	Device	40		Race and Ethnicity Not Collected	US	Randomized, Parallel Assignment	Research Institute	2012
Evaluation of Blood Glucose Monitoring Systems With Blood Samples From Neonates	Diabetes	Device	162		Race and Ethnicity Not Collected	US	Single Group Assignment	Industry	2012
Exposure Therapy for Veterans With PTSD and Panic Attacks (Phase 1)	Mental Illness	Behavioral	7		Race and Ethnicity Not Collected	US	Single Group Assignment	Research Institute	2012
FK506 (Tacrolimus) in Pulmonary Arterial Hypertension	Respiratory Disease	Drug	23		Race and Ethnicity Not Collected	US	Randomized, Parallel Assignment	University	2012
GLASSIA Infusion Rate Study	Respiratory Disease	Biological	30		Race and Ethnicity Not Collected	US	Randomized, Crossover Assignment	Industry	2012
Group Treatment for PTSD: A Randomized Clinical Trial With Veterans	Mental Illness	Behavioral	198	3	American Indian or Alaska Native	US	Randomized, Parallel Assignment	Research Institute	2012
HeartLight Ablation in Patients With Paroxysmal Atrial Fibrillation (PAF)	Cardiovascular Disease	Device	405	0		US	Randomized, Parallel Assignment	Industry	2012
Investigation of Efficacy of incobotulinumtoxinA (Xeomin) in Relieving Symptoms of Restless Leg Syndrome.	Mental Illness	Drug	24		Race and Ethnicity Not Collected	US	Randomized, Crossover Assignment	University	2012
Investigation of Sacroiliac Fusion Treatment (INSITE)	Arthritis	Device	159	1	American Indian	US	Randomized, Parallel Assignment	Industry	2012
Liraglutide in Obesity and Diabetes: Identification of CNS Targets Using fMRI	Diabetes	Drug	28		Race and Ethnicity Not Collected	US	Randomized, Crossover Assignment	Hospital	2012
Low Dose Naltrexone-buprenorphine Transfer to Vivitrol Injection in Opioid Dependence	Mental Illness	Drug	38	0		US	Single Group Assignment	Industry	2012
Magnesium Supplements In The Treatment Of Pseudoxanthoma Elasticum (PXE)	Cardiovascular Disease	Drug	44		Race and Ethnicity Not Collected	US	Randomized, Parallel Assignment	Industry	2012
Mantram Repetition Meditation for Veterans With PTSD	Mental Illness	Behavioral	181	31	American Indian or Alaska Native, Native Hawaiian or Other Pacific Islander	US	Randomized, Parallel Assignment	Research Institute	2012

Manuka Honey in Preventing Esophagitis-Related Pain in Patients Receiving Chemotherapy and Radiation Therapy For Lung Cancer	Respiratory Disease	Drug	163	2	American Indian or Alaska Native	US	Randomized, Parallel Assignment	NIH	2012
Multiple Ascending Dose Study of the Safety, Tolerability, Pharmacokinetic/Efficacy	Mental Illness	Drug	45	0		US	Non-randomized, Factorial Assignment	Industry	2012
Nitrites, Exercise, and Peripheral Arterial Disease	Cardiovascular Disease	Drug	32	0		US	Randomized, Parallel Assignment	NIH	2012
Novel Use of (Oral) Ketotifen for the Treatment of Fibromyalgia: A Pilot Study	Arthritis	Drug	51		Race and Ethnicity Not Collected	US	Randomized, Parallel Assignment	University	2012
Oral Nitrite in Adults With Metabolic Syndrome and Hypertension	Hypertension	Drug	20	2	American Indian or Alaska Native	US	Single Group Assignment	University	2012
Persistent Methicillin Resistant Staphylococcus Aureus Eradication Protocol (PMEP)	Respiratory Disease	Drug	29	0		US	Randomized, Parallel Assignment	University	2012
Pharmacodynamic Evaluation of PL2200 Versus Enteric-Coated Aspirin in Diabetic Patients	Diabetes	Drug	40	0		US	Randomized, Crossover Assignment	Industry	2012
Phase 1 Study to Assess the Safety/Tolerability of Brexpiprazole as Adjunctive Therapy in Elderly Subjects With Major Depressive Disorder	Mental Illness	Drug	18		Race and Ethnicity Not Collected	US	Randomized, Parallel Assignment	Industry	2012
Point-of-Care Glucose Testing and Insulin Supplementation	Diabetes	Drug	235	0		US	Randomized, Parallel Assignment	University	2012
PT001 MDI Versus SpirivaÂ® in Patients With Moderate to Severe COPD	Respiratory Disease	Drug	140	0		US	Randomized, Crossover Assignment	Industry	2012
Safety and Tolerability Trial of Aripiprazole IM Depot Treatment in Adult Subjects With Schizophrenia Stabilized on Oral Antipsychotics Other Than Aripiprazole	Mental Illness	Drug	60	0		US	Single Group Assignment	Industry	2012
SCD Use to Prevent Deep Venous Thrombosis (DVT) in Patients With PICC Lines	Cardiovascular Disease	Device	78		Race and Ethnicity Not Collected	US	Randomized, Parallel Assignment	Hospital	2012
SPD489 Low Dose and High Dose Ranges When Added to Stable Doses of Antipsychotic Medications in Clinically Stable Adults With Negative Symptoms of Schizophrenia	Mental Illness	Drug	0		Race and Ethnicity Not Collected	US	Randomized, Parallel Assignment	Industry	2012
Stereotactic Body Radiotherapy (SBRT) Versus Stereotactic Body Proton Therapy (SBPT)	Respiratory Disease	Radiation	21	0		US	Randomized, Parallel Assignment	NIH	2012
Study of Elotuzumab in Combination With Lenalidomide and Dexamethasone in Subjects With Multiple Myeloma and Various Levels of Renal Function	Cardiovascular Disease	Drug	35	0		US	Non-randomized, Parallel Assignment	Industry	2012

STX-100 in Patients With Idiopathic Pulmonary Fibrosis (IPF)	Respiratory Disease	Drug	41	0		US	Randomized, Parallel Assignment	Industry	2012
Tadalafil and Nesiritide as Therapy in Pre-clinical Heart Failure	Cardiovascular Disease	Drug	43		Race and Ethnicity Not Collected	US	Randomized, Crossover Assignment	NIH	2012
Tandem Auto Transplantation in Myeloma Patients With <12 Months of Prior Treatment	Cardiovascular Disease	Drug	19		Race and Ethnicity Not Collected	US	Single Group Assignment	NIH	2012
Temozolomide With or Without Veliparib in Treating Patients With Relapsed or Refractory Small Cell Lung Cancer	Respiratory Disease	Other	97	0		US	Randomized, Parallel Assignment	NIH	2012
Test-retest Reproducibility of [11C]PHNO PET Using the Constant Infusion Paradigm	Mental Illness	Drug	10	0		US	Single Group Assignment	NIH	2012
Thalidomide, Lenalidomide, and Rituximab for Previously Treated Waldenstrom Macroglobulinemia	Cardiovascular Disease	Drug	4		Race and Ethnicity Not Collected	US	Single Group Assignment	University	2012
The Effect of Immediate Implant Placement and Provisionalization in the Esthetic Zone	Dental Disease	Device	5		Race and Ethnicity Not Collected	US	Randomized, Parallel Assignment	University	2012
The PARTNER II Trial: Placement of AoRTic TraNscathetER Valves II - PARTNER II - Nested Registry 3/Valve-in-Valve	Cardiovascular Disease	Device	197		Race and Ethnicity Not Collected	US	Single Group Assignment	Industry	2012
The Penumbra Liberty Trial: Safety and Effectiveness in the Treatment of Wide-Neck Intracranial Aneurysms	Cardiovascular Disease	Device	112	1	American Indian	US	Single Group Assignment	Industry	2012
The Relationship of Hemoglobin A1c and Diabetic Wound Healing	Diabetes	Drug	2		Race and Ethnicity Not Collected	US	Randomized, Parallel Assignment	Hospital	2012
Tilt-Table Study of the Clinical Efficacy of Midodrine in Symptomatic Orthostatic Hypotension	Hypertension	Drug	20		Race and Ethnicity Not Collected	US	Randomized, Crossover Assignment	Industry	2012
Tobacco Dependence Treatment Education for Dental Students	Mental Illness	Other	94		Race and Ethnicity Not Collected	US	Non-randomized, Parallel Assignment	University	2012
Treatment of Post-Traumatic Brain Injury (Post-TBI) Fatigue With Light Therapy	Mental Illness	Device	88	7	Asian/Pacific Islande	US	Randomized, Parallel Assignment	University	2012
Using mTOR Inhibitors in the Prevention of BK Nephropathy	Kidney Disease	Drug	40	2	American Indian or Alaska Native, Native Hawaiian or Other Pacific Islander	US	Randomized, Parallel Assignment	University	2012
Value of Liquid Potassium Magnesium Citrate in Controlling Hypertension	Hypertension	Drug	30		Race and Ethnicity Not Collected	US	Randomized, Crossover Assignment	University	2012

Velcade + Cyclophosphamide in Newly Diagnosed Multiple Myeloma	Cardiovascular Disease	Drug	17		Race and Ethnicity Not Collected	US	Single Group Assignment	Industry	2012
VERIFY:A Study to Compare Combination Regimen With Vildagliptin & Metformin Versus Metformin in Treatment-naÃ¯ve Patients With Type 2 Diabetes Mellitus	Diabetes	Drug	2001	210	Native Americans	US	Randomized, Parallel Assignment	Industry	2012
Veterans Service Organizations and My HealtheVet (MHV)	Cardiovascular Disease	Other	282		Race and Ethnicity Not Collected	US	Randomized, Parallel Assignment	Research Institute	2012
Vet-Harts Pilot Intervention for Veterans With Coronary Heart Disease	Cardiovascular Disease	Behavioral	12	0		US	Randomized, Parallel Assignment	Research Institute	2012
Washington Study of Hemofiltration After Out-of-Hospital Cardiac Arrest	Cardiovascular Disease	Other	2		Race and Ethnicity Not Collected	US	Randomized, Parallel Assignment	University	2012
Weighted Versus Uniform Dose of Tranexamic Acid in Patients Undergoing Primary, Knee Arthroplasty	Arthritis	Drug	65		Race and Ethnicity Not Collected	US	Randomized, Parallel Assignment	University	2012
12-Month Open-Label Long-term Safety Study of TNX-102 SL Tablets in Fibromyalgia Patients	Arthritis	Drug	158		Race and Ethnicity Not Collected	US	Single Group Assignment	Industry	2013
A 26-week Trial Comparing Efficacy and Safety of Insulin Degludec/Insulin Aspart BID and Insulin Degludec OD Plus Insulin Aspart in Subjects With Type 2 Diabetes Mellitus Treated With Basal Insulin in Need of Treatment Intensification With Mealtime Insulin	Diabetes	Drug	274		Race and Ethnicity Not Collected	US	Randomized, Parallel Assignment	Industry	2013
A Clinical Evaluation of Absorbâ„¢ Bioresorbable Vascular Scaffold (Absorbâ„¢ BVS) System in Chinese Population ~ ABSORB CHINA Randomized Controlled Trial (RCT)	Cardiovascular Disease	Device	459		Race and Ethnicity Not Collected	US	Randomized, Parallel Assignment	Industry	2013
A Clinical Trial to Evaluate Long-term Efficacy and Safety of Lozenges Containing Lactobacilli Reuteri (Prodentisâ„¢) on Gingivitis	Dental Disease	Other	62	2	American Indian or Alaska Native, Native Hawaiian or Other Pacific Islander	US	Randomized, Parallel Assignment	Industry	2013
A Double Blind Clinical Trial of DCS for Food Anxiety	Mental Illness	Drug	36		Race and Ethnicity Not Collected	US	Randomized, Parallel Assignment	NIH	2013
A Helping Hand Among Low-Income Patients	Diabetes	Behavioral	348	0		US	Randomized, Parallel Assignment	University	2013
A Pharmacokinetic Substudy of the TDE-PH-304 Protocol	Hypertension	Drug	13	0		US	Single Group Assignment	Industry	2013
A Phase 3 Rollover Study of Lumacaftor in Combination With Ivacaftor in Subjects 12 Years and Older With Cystic Fibrosis	Respiratory Disease	Drug	1164		Race and Ethnicity Not Collected	US	Randomized, Parallel Assignment	Industry	2013

A Study of Lumacaftor in Combination With Ivacaftor in Cystic Fibrosis Subjects Aged 12 Years and Older Who Are Homozygous for the F508del-CFTR Mutation	Respiratory Disease	Drug	559		Race and Ethnicity Not Collected	US	Randomized, Parallel Assignment	Industry	2013
A Study of LY2940680 in Small Cell Lung Cancer	Respiratory Disease	Drug	26	0		US	Randomized, Parallel Assignment	Industry	2013
A Study of the Impact of Methotrexate (MTX) Discontinuation on the Efficacy of Subcutaneous (SC) Tocilizumab (TCZ) With MTX	Arthritis	Drug	718		Race and Ethnicity Not Collected	US	Randomized, Parallel Assignment	Industry	2013
A Study to Demonstrate the Benefit of a New Kind of Anti-cancer Treatment [PReferentially Expressed Antigen of MElanoma (PRAME) Immunotherapy] for Patients With Non-Small Cell Lung Cancer (NSCLC), After Removal of Their Tumor	Respiratory Disease	Biological	137		Race and Ethnicity Not Collected	US	Randomized, Parallel Assignment	Industry	2013
A Study To Examine The Safety, Tolerability And Pharmacokinetics Of PF-02545920 In Psychiatrically Stable Subjects With Schizophrenia	Mental Illness	Drug	37		Race and Ethnicity Not Collected	US	Randomized	Industry	2013
A Trial To Assess Risk of Delirium in Older Adults Undergoing Hip Fracture Surgery With Spinal or General Anesthesia	Mental Illness	Other	12		Race and Ethnicity Not Collected	US	Randomized, Parallel Assignment	University	2013
Acetaminophen Versus Ibuprofen in Children With Asthma	Respiratory Disease	Drug	300	8	American Indian or Alaska Native, Native Hawaiian or Other Pacific Islander	US	Randomized, Parallel Assignment	NIH	2013
Acute Effects of Inorganic Nitrite on Cardiovascular Hemodynamics in Heart Failure With Preserved Ejection Fraction	Cardiovascular Disease	Drug	28	0		US	Randomized, Parallel Assignment	Industry	2013
Alveolar Microperforation for Inflammation-Enhanced Tooth Movement During Orthodontic Treatment	Dental Disease	Device	21		Race and Ethnicity Not Collected	US	Randomized, Parallel Assignment	University	2013
An Outpatient Study Of The Efficacy, Safety, And Tolerability Of PF-02545920 In The Adjunctive Treatment Of Sub-Optimally Controlled Symptoms of Schizophrenia	Mental Illness	Drug	240		Race and Ethnicity Not Collected	US	Randomized, Parallel Assignment	Industry	2013
AngelÂ® Catheter Early Feasibility Clinical Study	Respiratory Disease	Device	5		Race and Ethnicity Not Collected	US	Single Group Assignment	Industry	2013
Antipsychotic Effects on Brain Function in Schizophrenia	Mental Illness	Drug	4		Race and Ethnicity Not Collected	US	Randomized, Parallel Assignment	University	2013
Aripiprazole Once-monthly in Patients With Schizophrenia	Mental Illness	Drug	88	0		US	Single Group Assignment	Industry	2013
Artificial Pancreas Control System in an Outpatient Setting	Diabetes	Device	20		Race and Ethnicity Not Collected	US	Single Group Assignment	Research Institute	2013

AVERT Shock: Arginine Vasopressin During the Early Resuscitation of Traumatic Shock	Diabetes	Drug	101	0		US	Randomized, Parallel Assignment	University	2013
BEdtime Sublingual TNX-102 SL as Fibromyalgia Intervention Therapy (BESTFIT)	Arthritis	Drug	205		Race and Ethnicity Not Collected	US	Randomized, Parallel Assignment	Industry	2013
Bimatoprost Ocular Insert Compared to Topical Timolol Solution in Patients With Glaucoma or Ocular Hypertension	Hypertension	Drug	169		Race and Ethnicity Not Collected	US	Randomized, Parallel Assignment	Industry	2013
Brexpiprazole (OPC-34712) as an Adjunctive Treatment in Adults With Major Depressive Disorder and Anxiety Symptoms	Mental Illness	Drug	37	0		US	Single Group Assignment	Industry	2013
Brexpiprazole as Adjunctive Therapy With Major Depressive Disorder and an Inadequate Response to Previous Adjunctive Therapy	Mental Illness	Drug	61	1	American Indian or Alaska Native, Native Hawaiian or Other Pacific Islander	US	Single Group Assignment	Industry	2013
Cabozantinib-s-malate or Sunitinib Malate in Treating Patients With Previously Untreated Locally Advanced or Metastatic Kidney Cancer	Kidney Disease	Drug	157	2	American Indian or Alaska Native, Native Hawaiian or Other Pacific Islander	US	Randomized, Parallel Assignment	NIH	2013
Cangrelor Ticagrelor Transition Study	Cardiovascular Disease	Drug	12		Race and Ethnicity Not Collected	US	Randomized, Factorial Assignment	Industry	2013
Carfilzomib in Refractory Renal Cell Carcinoma (RCC)	Kidney Disease	Drug	10	0		US	Single Group Assignment	Industry	2013
Clinical Study of Macitentan in Patients With Pulmonary Arterial Hypertension to Psychometrically Validate the PAH-SYMPACT Instrument	Hypertension	Drug	284	3	American Indian or Alaska Native, Native Hawaiian or Other Pacific Islander	US	Single Group Assignment	Industry	2013
Comparing Types of Implementation of a Shared Decision Making Intervention	Respiratory Disease	Other	6274		Race and Ethnicity Not Collected	US	Randomized, Parallel Assignment	Research Institute	2013
Comparison of Curosurf and Infasurf in the Treatment of Preterm Infants With Respiratory Distress Syndrome	Respiratory Disease	Drug	30		Race and Ethnicity Not Collected	US	Randomized, Parallel Assignment	University	2013
Control of Cognition (Naltrexone, Methylphenidate, and ADHD Study (NMAS))	Mental Illness	Drug	23	0		US	Randomized, Parallel Assignment	University	2013

Dane County Drug Court Study for Addicted Offenders	Mental Illness	Drug	24		Race and Ethnicity Not Collected	US	Randomized, Parallel Assignment	University	2013
Decision Support for Smoking Cessation in Young Adults With Severe Mental Illness	Mental Illness	Behavioral	58	0		US	Randomized, Parallel Assignment	NIH	2013
Development of Walk Assist Device to Improve Community Ambulation	Cardiovascular Disease	Device	53		Race and Ethnicity Not Collected	US	Randomized, Parallel Assignment	Industry	2013
DISKUS vs. ELLIPTA Device Preference Study in Chronic Obstructive Pulmonary Disease (COPD)	Respiratory Disease	Device	287	0		US	Randomized, Crossover Assignment	Industry	2013
Dose-Response of Salmeterol in Children	Respiratory Disease	Drug	10		Race and Ethnicity Not Collected	US	Randomized, Crossover Assignment	University	2013
Early Warning System	Cardiovascular Disease	Other	571		Race and Ethnicity Not Collected	US	Randomized, Parallel Assignment	University	2013
Effect of Antioxidant Vitamins on Coagulopathy and Nosocomial Pneumonia After Severe Trauma	Respiratory Disease	Dietary Supplement	11	0		US	Randomized, Parallel Assignment	University	2013
Effectiveness of DECIDE in Patient-Provider Communication, Therapeutic Alliance & Care Continuation	Mental Illness	Behavioral	481	7	American Indian or Alaska Native, Native Hawaiian or Other Pacific Islander	US	Randomized, Parallel Assignment	Hospital	2013
Effects of Cerebral Hypoperfusion and Its Reversal on Late-Life Depression	Mental Illness	Drug	1		Race and Ethnicity Not Collected	US	Single Group Assignment	University	2013
Efficacy of Optison Echo Contrast to Detect Thrombus in Left Atrial Appendage	Cardiovascular Disease	Drug	100	2	Native Hawaiian or Other Pacific Islander	US	Single Group Assignment	University	2013
Environmental Intervention to Reduce Allergens in Urban Schools and Childhood Asthma	Respiratory Disease	Other	29	0		US	Randomized, Parallel Assignment	Hospital	2013
Erlotinib Hydrochloride or Crizotinib and Chemoradiation Therapy in Treating Patients With Stage III Non-small Cell Lung Cancer	Respiratory Disease	Radiation	59	0		US	Randomized, Parallel Assignment	NIH	2013
Evaluating Two Types of Cognitive Training in Veterans With Schizophrenia	Mental Illness	Behavioral	105	0		US	Randomized, Parallel Assignment	Research Institute	2013
EverFlex Post Approval Study	Cardiovascular Disease	Device	108	0		US	Single Group Assignment	Industry	2013
Fatty Liver Study in Patients With Type II Diabetes	Diabetes	Drug	2		Race and Ethnicity Not Collected	US	Randomized, Parallel Assignment	University	2013

Group Learning Achieves Decreased Incidents of Lower Urinary Symptoms	Kidney Disease	Behavioral	463	5	Asian/ Pacific Islander, Native American	US	Randomized, Parallel Assignment	NIH	2013
High Ticagrelor Loading Dose in STEMI	Cardiovascular Disease	Drug	52	0		US	Randomized, Parallel Assignment	University	2013
Improving Buprenorphine Detoxification Outcomes With Isradipine	Mental Illness	Drug	28	0		US	Randomized, Parallel Assignment	NIH	2013
Improving Communication About Serious Illness	Cardiovascular Disease	Behavioral	817	8	Pacific Islander, Native American	US	Randomized, Parallel Assignment	University	2013
Informing Policy to Implement Pediatric Family Engagement in Meaningful Use Stage 3	Respiratory Disease	Other	294	0		US	Single Group Assignment	Hospital	2013
Ipilimumab and Local Radiation Therapy in Treating Patients With Recurrent Melanoma, Non-Hodgkin Lymphoma, Colon, or Rectal Cancer	Cardiovascular Disease	Biological	3	0		US	Single Group Assignment	NIH	2013
IVIg Efficacy Study to Treat Cutaneous Lupus Erythematosus	Arthritis	Drug	16		Race and Ethnicity Not Collected	US	Single Group Assignment	University	2013
Ketamine for Suicidality in Bipolar Depression	Mental Illness	Drug	16	0		US	Randomized, Parallel Assignment	Research Institute	2013
Lipid Biomarkers for Diabetic Heart Disease	Diabetes	Drug	70	0		US	Randomized, Parallel Assignment	NIH	2013
Mechanisms of Sleep Disruption Hyperalgesia	Mental Illness	Drug	100	0		US	Randomized, Crossover Assignment	NIH	2013
MI Varnish and MI Paste Plus in a Caries Prevention and Remineralization Study	Dental Disease	Drug	40		Race and Ethnicity Not Collected	US	Randomized, Parallel Assignment	University	2013
Mitigation of Radiation Pneumonitis and Fibrosis	Respiratory Disease	Drug	50		Race and Ethnicity Not Collected	US	Randomized, Parallel Assignment	Research Institute	2013
Naltrexone for Antipsychotic-Induced Weight Gain	Mental Illness	Drug	144	0		US	Randomized, Parallel Assignment	NIH	2013
Nesiritide and Renal Function After the Total Artificial Heart	Cardiovascular Disease	Drug	2		Race and Ethnicity Not Collected	US	Randomized, Parallel Assignment	University	2013
OFDI Capsule Imaging in Patients With Atrial Fibrillation Undergone Radio Frequency (RF) Ablation	Cardiovascular Disease	Device	5	0		US	Single Group Assignment	NIH	2013
Omega-3 Dietary Supplements in Schizophrenia	Mental Illness	Drug	50		Race and Ethnicity Not Collected	US	Single Group Assignment	NIH	2013

Open-label, Extension Study of Aripiprazole Intramuscular Depot (OPC-14597, Lu AF41155) in Patients With Schizophrenia	Mental Illness	Drug	74		Race and Ethnicity Not Collected	US	Single Group Assignment	Industry	2013
Outpatient Reduction of Nocturnal Hypoglycemia by Using Predictive Algorithms and Pump Suspension in Children	Diabetes	Device	82	0		US	Randomized, Parallel Assignment	NIH	2013
PET-Adjusted Intensity Modulated Radiation Therapy and Combination Chemotherapy in Treating Patients With Stage II-IV Non-small Cell Lung Cancer	Respiratory Disease	Drug	35		Race and Ethnicity Not Collected	US	Single Group Assignment	NIH	2013
Pharmacodynamic Effect of Prasugrel vs. Ticagrelor in Diabetes	Diabetes	Drug	50	0		US	Randomized, Crossover Assignment	University	2013
Pharmacokinetic Study of Oral IXAZOMIB in Cancer Patients With Liver Dysfunction	Cardiovascular Disease	Drug	48	0		US	Non-randomized, Parallel Assignment	Industry	2013
Phase 1b/2 Study of Carfilzomib, Carboplatin, and Etoposide in Patients With Previously Untreated Extensive Stage Small-cell Lung Cancer	Respiratory Disease	Drug	32	0		US	Sequential Assignment	Industry	2013
Pilot Study: Lipoic Acid and Omega-3 Fatty Acids for Alzheimer's Prevention	Hypertension	Drug	42	0		US	Randomized, Parallel Assignment	University	2013
Pilot Trial of Phototherapy for Acute Depression in Hospitalized Cystic Fibrosis Patients	Respiratory Disease	Device	30		Race and Ethnicity Not Collected	US	Single Group Assignment	Hospital	2013
Preventing Post-Operative Delirium in Patients Undergoing a Pneumonectomy, Esophagectomy or Thoracotomy	Mental Illness	Drug	135	0		US	Randomized, Parallel Assignment	University	2013
Real-Time Mobile Cognitive Behavioral Intervention for Serious Mental Illness	Mental Illness	Behavioral	255	1	American Indian or Alaska Native	US	Randomized, Parallel Assignment	University	2013
Regadenoson Stress-MRI to Identify Coronary Artery Disease in Atrial Fibrillation Patients	Cardiovascular Disease	Drug	30		Race and Ethnicity Not Collected	US	Single Group Assignment	University	2013
Safety and Efficacy of Merlin (Ethanol and Glycolic Acid Mixture) for Episodic Treatment of Cold Sores	Dental Disease	Drug	469	2	American Indian or Alaska Native	US	Randomized, Parallel Assignment	Industry	2013
Safety, Efficacy and Tolerability of Vilazodone in Generalized Anxiety Disorder	Mental Illness	Drug	414	2	American Indian or Alaska Native	US	Randomized, Parallel Assignment	Industry	2013
Safety, Tolerability and Effectiveness of Nuedexta in the Treatment of Pseudobulbar Affect (PBA)	Cardiovascular Disease	Drug	367	2	Native Hawaiian/pacific islander	US	Single Group Assignment	Industry	2013
Safety, Tolerability, Pharmacokinetic, and Efficacy Study of AZD5213 in Adolescents With Tourette's Disorder	Mental Illness	Drug	29	0		US	Randomized, Crossover Assignment	Industry	2013
Series of N-of-1 Crossover Trials of Antihypertensive Therapy in Adolescents With Essential Hypertension	Hypertension	Drug	42	0		US	Randomized, Crossover Assignment	University	2013

Shoe Lifts for Leg Length Inequality in Adults With Knee or Hip Symptoms	Arthritis	Device	46	0		US	Randomized, Parallel Assignment	University	2013
Study of Adrenalectomy Versus Observation for Subclinical Hypercortisolism	Cardiovascular Disease	Procedure	4	0		US	Randomized, Parallel Assignment	NIH	2013
Study of Flat Polyp Detection Using New Narrow Band Imaging (NBI) Compared to White Light Colonoscopy - The FIND FLAT Colonoscopy Study	Cardiovascular Disease	Device	281			US	Randomized, Parallel Assignment	Industry	2013
Study of Oasis Ultra in Diabetic Foot Ulcers	Diabetes	Other	82	0		US	Randomized, Parallel Assignment	Industry	2013
Study to Characterize the Local Duration of Exposure From FX006 in Patients With Osteoarthritis of the Knee	Arthritis	Drug	50		Race and Ethnicity Not Collected	US	Single Group Assignment	Industry	2013
Targeting the Right Ventricle in Pulmonary Hypertension	Respiratory Disease	Drug	22		Race and Ethnicity Not Collected	US	Randomized, Parallel Assignment	University	2013
Testing the Feasibility of Patient Controlled Sedation for Ventilated ICU Patients	Respiratory Disease	Drug	15	0		US	Randomized, Parallel Assignment	NIH	2013
The Effects of an Antioxidant Formulation on Ocular Blood Flow	Hypertension	Dietary Supplement	47	0		US	Randomized, Crossover Assignment	Industry	2013
The Influence of Febuxostat on Coronary Artery Endothelial Dysfunction in Participants With Chronic Stable Angina	Cardiovascular Disease	Drug	30	0		US	Randomized, Crossover Assignment	Industry	2013
The Summer Camp Study: Blood Glucose Control With a Bi-Hormonal Bionic Endocrine Pancreas	Diabetes	Other	32		Race and Ethnicity Not Collected	US	Randomized, Crossover Assignment	Hospital	2013
The Use of Paravertebral Block for Cardiac Surgery	Cardiovascular Disease	Other	60		Race and Ethnicity Not Collected	US	Single Group Assignment	University	2013
Ticagrelor Versus Clopidogrel in Type 2 Diabetic Patients	Diabetes	Drug	20		Race and Ethnicity Not Collected	US	Randomized, Crossover Assignment	University	2013
Tocilizumab in the Management of Juvenile Idiopathic Arthritis Associated Uveitis	Arthritis	Drug	3		Race and Ethnicity Not Collected	US	Single Group Assignment	University	2013
TRACER RGD-K5 Carotid Plaque Imaging Study	Cardiovascular Disease	Drug	3	0		US	Single Group Assignment	Hospital	2013
Tranexamic Acid in Reverse Total Shoulder Arthroplasty	Arthritis	Drug	102		Race and Ethnicity Not Collected	US	Randomized, Parallel Assignment	Hospital	2013
Treatment of Complex Regional Pain Syndrome With Once Daily Gastric-Retentive Gabapentin (Gralise)	Mental Illness	Drug	5		Race and Ethnicity Not Collected	US	Single Group Assignment	Hospital	2013

TXV13-01 Estradiol Vaginal Softgel Capsules in Treating Postmenopausal Women With Symptoms of Vulvar and Vaginal Atrophy	Mental Illness	Drug	50	0		US	Randomized, Parallel Assignment	Industry	2013
Use of Immune Globulin Plus Rituximab for Desensitization in Highly HLA Sensitized Patients Awaiting Deceased Donor Kidney Transplantation	Kidney Disease	Biological	41	0		US	Single Group Assignment	Industry	2013
Use of Mobile Devices and the Internet to Streamline an Asthma Clinical Trial	Respiratory Disease	Drug	79	2	American Indian or Alaska Native	US	Randomized, Parallel Assignment	NIH	2013
Vascular Inflammation in Psoriasis - Extension Study	Arthritis	Drug	81	1	American Indian or Alaska Native	US	Single Group Assignment	University	2013
24 Week Efficacy and 3-year Safety and Efficacy of Secukinumab in Active Psoriatic Arthritis	Arthritis	Biological	414	2	American Indian or Alaska Native	US	Randomized, Parallel Assignment	Industry	2014
24 Week Efficacy and Safety Study of Empagliflozin (BI 10773) in Hypertensive Black/African American Patients With Type 2 Diabetes Mellitus and Hypertension	Diabetes	Drug	150	0		US	Randomized, Parallel Assignment	Industry	2014
A Clinical Study in Participants With Huntington's Disease (HD) to Assess Efficacy and Safety of Three Oral Doses of Laquinimod	Mental Illness	Drug	352	0		US	Randomized, Parallel Assignment	Industry	2014
A Concierge Model of CAE Plus LAI in Individuals With Schizophrenia at Risk for Treatment Non-adherence and Homelessness	Mental Illness	Behavioral	30	0		US	Single Group Assignment	University	2014
A Dose Ranging Phase IIa Study of 6 Hour Intravenous Dosages of CXL-1427 in Patients Hospitalized With Heart Failure	Cardiovascular Disease	Drug	70		Race and Ethnicity Not Collected	US	Randomized, Parallel Assignment	Industry	2014
A Double-blind Study to Assess the Efficacy and Safety of Intranasal Esketamine for the Rapid Reduction of the Symptoms of Major Depressive Disorder, Including Suicidal Ideation, in Participants Who Are Assessed to be at Imminent Risk for Suicide	Mental Illness	Drug	68		Race and Ethnicity Not Collected	US	Randomized, Parallel Assignment	Research Institute	2014
A Long-Term Extension Study of OnabotulinumtoxinA (BOTOXÂ®) for Urinary Incontinence Due to Neurogenic Detrusor Overactivity	Mental Illness	Biological	95	0		US	Non-Randomized, Parallel Assignment	Industry	2014
A Mobile Personal Health Record for Behavioral Health Homes	Diabetes	Behavioral	311	0		US	Randomized, Parallel Assignment	NIH	2014
A Multicenter, Postmarketing Study Evaluating the Concentration of CimziaÂ® in Mature Breast Milk of Lactating Mothers	Arthritis	Procedure	18		Race and Ethnicity Not Collected	US	Single Group Assignment	Industry	2014
A Phase 2 Study of Viagenpumatucel-L (HS-110) in Patients With Non-Small Cell Lung Cancer	Respiratory Disease	Drug	66	0		US	Randomized, Parallel Assignment	Industry	2014
A Phase 3a, Repeat Dose, Open-label, Long-term Safety Study of Mepolizumab in Asthmatic Subjects	Respiratory Disease	Biological	339	0		US	Single Group Assignment	Industry	2014

A Phase II Study of Doxycycline in Relapsed NHL	Cardiovascular Disease	Drug	7		Race and Ethnicity Not Collected	US	Single Group Assignment	University	2014
A Phase IIa Study to Investigate the Efficacy and Safety of AZD7624 in Chronic Obstructive Pulmonary Disease (COPD) Patients While on Maintenance Therapy	Respiratory Disease	Drug	213	7	American Indian or Alaska Native	US	Randomized, Parallel Assignment	Industry	2014
A Study Comparing Cardiovascular Effects of Ticagrelor Versus Placebo in Patients With Type 2 Diabetes Mellitus	Diabetes	Drug	19271	327	American Indian or Alaska Native, Native Hawaiian or Other Pacific Islander	US	Randomized, Parallel Assignment	Industry	2014
A Study of IDN-6556 in Cirrhotic Subjects With Portal Hypertension	Cardiovascular Disease	Drug	23		Race and Ethnicity Not Collected	US	Non-randomized, Single Group Assignment	Industry	2014
A Study of LY2409021 on Blood Pressure and Pulse Rate in Participants With Type 2 Diabetes Mellitus	Diabetes	Drug	270	42	American Indian or Alaska Native	US	Randomized, Crossover Assignment	Industry	2014
A Study of Tabalumab (LY2127399) Using Two Different Injection Methods in Participants With Lupus	Arthritis	Drug	226	0		US	Randomized, Parallel Assignment	Industry	2014
A Study of the Gore VIABAHN BX for Treatment of Occlusive Disease in the Iliac Arteries.	Cardiovascular Disease	Device	134	2	American Indian or Alaska Native	US	Single Group Assignment	Industry	2014
A Study to Evaluate the Effect of Long-term Treatment With BELVIQ (Lorcaserin HCl) on the Incidence of Major Adverse Cardiovascular Events and Conversion to Type 2 Diabetes Mellitus in Obese and Overweight Subjects With Cardiovascular Disease or Multiple Cardiovascular Risk Factors	Diabetes	Drug	14673	100	American Indian or Alaska Native, Native Hawaiian or Other Pacific Islander	US	Randomized, Parallel Assignment	Industry	2014
Acceptance and Commitment Therapy for the Inpatient Treatment of Psychosis	Mental Illness	Behavioral	18	0		US	Randomized, Parallel Assignment	Industry	2014
Achieving Cannabis Cessation-Evaluating N-Acetylcysteine Treatment	Mental Illness	Drug	302	3	American Indian or Alaska Native, Native Hawaiian or Other Pacific Islander	US	Randomized, Parallel Assignment	NIH	2014
Adjunctive Neurovascular Support for Wide-neck Aneurysm Embolization and Reconstruction	Cardiovascular Disease	Device	34		Race and Ethnicity Not Collected	US	Single Group Assignment	Industry	2014

An RCT Comparing Xenograft and Allograft for Ridge Preservation	Dental Disease	Device	40		Race and Ethnicity Not Collected	US	Single Group Assignment	Research Institute	2014
ARISTOCRAT-A Randomized Controlled Trial Evaluating Closure Following Access With the AXERA (Device Name) 2 Access System	Cardiovascular Disease	Device	39		Race and Ethnicity Not Collected	US	Randomized, Parallel Assignment	Research Institute	2014
Artifact-Free High-Resolution Myocardial Perfusion MRI in Subjects With Abnormal Nuclear Myocardial Perfusion Studies	Cardiovascular Disease	Drug	44		Race and Ethnicity Not Collected	US	Single Group Assignment	Industry	2014
Atomoxetine in Veterans With Comorbid ADHD/PTSD	Mental Illness	Drug	44	0		US	Randomized, Crossover Assignment	Research Institute	2014
Best African American Response to Asthma Drugs	Respiratory Disease	Drug	574	0		US	Randomized, Crossover Assignment	Hospital	2014
Brief Behavioral Therapy in Improving Sleep Disorders in Patients With Stage I-III Breast Cancer Undergoing Chemotherapy	Mental Illness	Behavioral	71	0		US	Randomized, Parallel Assignment	University	2014
Care Coordination/Home Telehealth to Safeguard Care in CKD	Kidney Disease	Other	118	0		US	Randomized, Parallel Assignment	NIH	2014
Clinical Evaluation of BackStop in Patients Undergoing Ureteroscopic Lithotripsy	Kidney Disease	Device	35	0		US	Randomized, Parallel Assignment	University	2014
Clinical Trial Tobacco Marijuana	Mental Illness	Behavioral	67	0		US	Randomized, Parallel Assignment	NIH	2014
Closed Loop Insulin Pump Therapy After Islet Auto-Transplantation	Diabetes	Device	14		Race and Ethnicity Not Collected	US	Randomized, Parallel Assignment	University	2014
Closed-Loop Glucagon Administration For Hypoglycemia Treatment	Diabetes	Device	31		Race and Ethnicity Not Collected	US	Randomized, Crossover Assignment	University	2014
Comparison of Treatment for Hoarding Disorder	Mental Illness	Behavioral	323	4	American Indian or Alaska Native, Native Hawaiian or Other Pacific Islander	US	Randomized, Parallel Assignment	University	2014
Decision Aids for the Management of Suspicious Occlusal Caries Lesions	Dental Disease	Device	3085	79	American Indian or Alaska Native, Native Hawaiian or Other Pacific Islander	US	Randomized, Parallel Assignment	University	2014

Developing Effective Response Inhibition Training for Symptom Relief in OCD and Trichotillomania	Mental Illness	Behavioral	45	0		US	Randomized, Parallel Assignment	NIH	2014
Development & Testing of a Decision Aid for LVAD Placement	Cardiovascular Disease	Other	98	0		US	Randomized, Parallel Assignment	University	2014
Dexamethasone for Treatment of Radiation-related Fatigue in Patients Receiving RT for Head-neck and Lung Cancer	Respiratory Disease	Drug	0		Race and Ethnicity Not Collected	US	Randomized, Parallel Assignment	University	2014
Dexamethasone Versus Burr Hole Craniostomy for Symptomatic Chronic Subdural Hematoma	Cardiovascular Disease	Drug	10		Race and Ethnicity Not Collected	US	Randomized, Parallel Assignment	University	2014
Dose Response Effects of Quillivant XR in Children With ADHD and Autism: A Pilot Study	Mental Illness	Drug	36		Race and Ethnicity Not Collected	US	Randomized, Parallel Assignment	Hospital	2014
Effect of Combined Incretin-Based Therapy Plus Canagliflozin on Glycemic Control and the Compensatory Rise in Hepatic Glucose Production in Type 2 Diabetic Patients	Diabetes	Drug	45		Race and Ethnicity Not Collected	US	Single Group Assignment	University	2014
Efficacy and Safety Study of PT009, PT008, and PT005 in Subjects With Moderate to Severe Chronic Obstructive Pulmonary Disease (COPD)	Respiratory Disease	Drug	180		Race and Ethnicity Not Collected	US	Randomized, Crossover Assignment	Industry	2014
Efficacy Study of Riociguat and Its Effects on Exercise Performance and Pulmonary Artery Pressure at High Altitude	Hypertension	Drug	22		Race and Ethnicity Not Collected	US	Non-randomized, Parallel Assignment	University	2014
Efficacy Study on Silver-coated ETT Cleaned With a Novel Device	Respiratory Disease	Device	40	0		US	Randomized, Parallel Assignment	Hospital	2014
Efficacy, Safety And Tolerability Of PF-06743649 In Gout Subjects.	Arthritis	Drug	30		Race and Ethnicity Not Collected	US	Randomized, Parallel Assignment	Industry	2014
Enhancing Delivery of Problem Solving Therapy Using SmartPhone Technology	Mental Illness	Other	33	1	Native Americans	US	Randomized, Parallel Assignment	Research Institute	2014
Evaluation of Anti-platelet Factor 4/Heparin Antibodies in Hemodialysis Patients	Kidney Disease	Device	42	0		US	Randomized, Parallel Assignment	Industry	2014
Extended-Release vs. Oral Naltrexone Alcohol Treatment in Primary Care	Mental Illness	Drug	237	1	American Indian or Alaska Native	US	Randomized, Parallel Assignment	University	2014
Galantamine and Memantine Combination for Cognitive Impairments in Schizophrenia	Mental Illness	Drug	3	0		US	Single Group Assignment	Industry	2014
Gastrointestinal Sensorimotor Dysfunctions in Diabetes Mellitus	Diabetes	Drug	104		Race and Ethnicity Not Collected	US	Randomized, Parallel Assignment	NIH	2014
Genomics Used to Improve DEpression Decisions	Mental Illness	Genetic	1398	9	American Indian or Alaska	US	Randomized, Parallel Assignment	University	2014

					Native, Native Hawaiian or Other Pacific Islander				
Heated Humidified Oxygen Compared to Dry Oxygen Therapy in Children With Bronchiolitis	Respiratory Disease	Device	32	0		US	Randomized, Parallel Assignment	Hospital	2014
Human Mesenchymal Stromal Cells For Acute Respiratory Distress Syndrome (START)	Respiratory Disease	Biological	60	3	Native Hawaiian or other Pacific Islander	US	Randomized, Parallel Assignment	NIH	2014
Improving Cognition in Schizophrenia Using tDCS	Mental Illness	Device	37		Race and Ethnicity Not Collected	US	Randomized, Crossover Assignment	University	2014
Incentives, Cognitive Training and Internet Therapy for Teens With Poorly Controlled Type 1 Diabetes	Diabetes	Behavioral	61	0		US	Randomized, Parallel Assignment	NIH	2014
Inpatient Diabetes on Corticosteroids	Diabetes	Drug	85		Race and Ethnicity Not Collected	US	Randomized, Parallel Assignment	Research Institute	2014
Jump Start Shared Medical Appointments for Diabetes With Weight Management	Diabetes	Behavioral	263	3	American Indian or Alaska Native	US	Randomized, Parallel Assignment	Research Institute	2014
Latinos Combating Diabetes	Diabetes	Behavioral	225	0		US	Randomized, Parallel Assignment	NIH	2014
LCI-LUN-ABR-001: Carbo With Nab-Paclitaxel in Patients With Advanced NSCL Cancer	Respiratory Disease	Drug	11	0		US	Single Group Assignment	Industry	2014
Loving-Kindness Meditation for PTSD	Mental Illness	Behavioral	184	3	American Indian or Alaska Native	US	Randomized, Parallel Assignment	Research Institute	2014
Microvascular Assessment of Ranolazine in Non-Obstructive Atherosclerosis (MARINA)	Cardiovascular Disease	Drug	26	0		US	Randomized, Parallel Assignment	University	2014
MOMENTUM 3 IDE Clinical Study Protocol	Cardiovascular Disease	Device	1028	4	Native Hawaiian or other Pacific Islander	US	Randomized, Parallel Assignment	Industry	2014
Musculoskeletal Ultrasound in Predicting Early Dose Titration With Tocilizumab	Arthritis	Drug	74	2	American Indian or Alaska Native, Native Hawaiian or Other Pacific Islander	US	Single Group Assignment	University	2014
Nutrition and Aerobic Exercise in Chronic Stroke	Cardiovascular Disease	Behavioral	51	0		US	Randomized, Parallel Assignment	Research Institute	2014

Omega-3 for Depression and Other Cardiac Risk Factors - 2	Mental Illness	Drug	144	2	American Indian or Alaska Native	US	Randomized, Parallel Assignment	NIH	2014
Omega-3 Supplementation to ADHD Medication in Children	Mental Illness	Dietary Supplement	21		Race and Ethnicity Not Collected	US	Single Group Assignment	Hospital	2014
Open-Label Study of N-Acetylcysteine in Children and Adolescents 5-17 With Bipolar Spectrum Disorders	Mental Illness	Drug	40	1	American Indian or Alaska Native	US	Single Group Assignment	Hospital	2014
Open-label Study to Assess Usability of the Medical Information Device #1 (MIND1) System in Adults With Schizophrenia On Oral Aripiprazole	Mental Illness	Device	67	0		US	Single Group Assignment	Industry	2014
Person-Centered Versus Measurement-Based Care in Mental Health	Mental Illness	Behavioral	2443	9	Native American	US	Randomized, Parallel Assignment	University	2014
Phase 2 Study of Alisertib (MLN8237) in Combination With Paclitaxel Versus Placebo in Combination With Paclitaxel as Second Line Therapy for Small Cell Lung Cancer (SCLC)	Respiratory Disease	Drug	178	0		US	Randomized, Parallel Assignment	Industry	2014
Phase I Study of Nicotinamide for Early Onset Preeclampsia	Hypertension	Drug	10		Race and Ethnicity Not Collected	US	Single Group Assignment	University	2014
Phase I Trial of Everolimus, Pomalidomide and Dexamethasone in Patients With Relapsed/Refractory Multiple Myeloma	Cardiovascular Disease	Drug	1		Race and Ethnicity Not Collected	US	Single Group Assignment	Industry	2014
Phase I/II Trial of the Combination of Lenalidomide (Revlimid) and Nab-paclitaxel (Abraxane) in the Treatment of Relapsed/Refractory Multiple Myeloma	Cardiovascular Disease	Drug	3		Race and Ethnicity Not Collected	US	Single Group Assignment	University	2014
Pilot Study of Atorvastatin for Orthopedic Surgery Patients	Cardiovascular Disease	Drug	20	0		US	Randomized, Parallel Assignment	University	2014
Positron Emission Tomography (PET) Study Investigating Dopamine and Serotonin Receptor Occupancy After Multiple Oral Dosing of Lu AF35700	Mental Illness	Drug	22		Race and Ethnicity Not Collected	US	Non-randomized, Parallel Assignment	Industry	2014
Randomized Evaluation of Ten Allergy Skin Prick Test Devices	Respiratory Disease	Device	24		Race and Ethnicity Not Collected	US	Single Group Assignment	University	2014
Randomized Trial Comparing Sitagliptin to Placebo in Closed Loop	Diabetes	Drug	17	0		US	Single Group Assignment	University	2014
Safety of Brimonidine Tartrate Ophthalmic Solution in a Population of Pediatric, Adult, and Geriatric Participants	Cardiovascular Disease	Drug	507		Race and Ethnicity Not Collected	US	Randomized, Parallel Assignment	Industry	2014
Social Forces to Improve Statin Adherence (Study A)	Hypertension	Behavioral	200	2	American Indian or Alaska Native, Native Hawaiian or	US	Randomized, Parallel Assignment	University	2014

					Other Pacific Islander				
Specialized Community Disease Management to Reduce Substance Use and Hospital Readmissions	Cardiovascular Disease	Behavioral	97	0		US	Randomized, Parallel Assignment	Research Institute	2014
Study of Clonidine Hydrochloride Topical Gel, 0.1% in the Treatment of Pain Associated With Diabetic Neuropathy	Diabetes	Drug	260	3	American Indian or Alaska Native	US	Randomized, Parallel Assignment	Industry	2014
Study to Evaluate the Safety and Efficacy of GSK1278863 in Recombinant Human Erythropoietin (rhEPO) Hyporesponsive Hemodialysis-dependent Chronic Kidney Disease Subjects With Anemia	Kidney Disease	Drug	15	0		US	Single Group Assignment	Industry	2014
SYN120 Study to Evaluate Its Safety, Tolerability and Efficacy in Parkinson's Disease Dementia (SYNAPSE)	Mental Illness	Drug	82	0		US	Randomized, Parallel Assignment	Industry	2014
The Combination of Adductor Canal Block and Periarticular Injection. A Novel Technique for Patients Undergoing Total Knee Replacement (ACB PAI)	Arthritis	Drug	111	1	American Indian or Alaska Native	US	Randomized, Parallel Assignment	Hospital	2014
The Tobacco, Alcohol, Prescription Medication and Other Substances Tool	Mental Illness	Other	2000	0		US	Single Group Assignment	Research Institute	2014
The WEB-IT Clinical Study	Cardiovascular Disease	Device	150	0		US	Single Group Assignment	Industry	2014
To Evaluate the Effect of Inhaled Medication Together With Exercise and Activity Training on Exercise Capacity and Daily Activities in Patients With Chronic Lung Disease With Obstruction of Airways	Respiratory Disease	Drug	303		Race and Ethnicity Not Collected	US	Randomized, Parallel Assignment	Industry	2014
To Evaluate the Effect of Liraglutide on Ambulatory Blood Pressure-A Pilot Study	Cardiovascular Disease	Drug	11	0		US	Randomized, Parallel Assignment	University	2014
Transcranial Magnetic Stimulation Treatment of Hoarding Disorder	Mental Illness	Device	1		Race and Ethnicity Not Collected	US	Single Group Assignment	Hospital	2014
Treatment of Resistant Hypertension by Prevention of T-Cell Co-Stimulation	Hypertension	Drug	1		Race and Ethnicity Not Collected	US	Randomized, Parallel Assignment	University	2014
16-week Comparative Effectiveness Trial of Lamotrigine vs. Fluoxetine for Bipolar Depression	Mental Illness	Drug	2	0		US	Randomized, Parallel Assignment	Hospital	2015
5-hydroxytryptophan and Creatine for Treatment Resistant Depression Associated With Hypoxia in Females	Mental Illness	Drug	15	0		US	Single Group Assignment	University	2015
A Euglycemic Insulin Clamp Study in Type 1 Diabetic Patients With Oral Insulin (ORAMED)	Diabetes	Drug	11	0		US	Non-Randomized, Crossover Assignment	University	2015
A Phase 4 Trial Assessing the ImPact of Residual Inflammation Detected Via Imaging TEchniques, Drug Levels and Patient Characteristics on the Outcome of Dose TaperIng of Adalimumab in Clinical Remission Rheumatoid ArThritis (RA) Subjects	Arthritis	Biological	149	0		US	Randomized, Parallel Assignment	Industry	2015

A Phase II Study to Evaluate Safety and Efficacy of ALX-0061 in Subjects With Systemic Lupus Erythematosus	Arthritis	Biological	312	0		US	Randomized, Parallel Assignment	Industry	2015
A Phase IIb Study for ALX-0061 Monotherapy in Subjects With Rheumatoid Arthritis	Arthritis	Biological	251		Race and Ethnicity Not Collected	US	Randomized, Parallel Assignment	Industry	2015
A Pivotal Efficacy Trial to Evaluate HLD200 in Children With ADHD in a Classroom Setting	Mental Illness	Drug	125	2	Native Hawaiian or Other Pacific Islander	US	Randomized, Parallel Assignment	Industry	2015
A Proof of Concept Study of Intravenous Sodium Nitroprusside in Adults With Symptomatic Schizophrenia	Mental Illness	Drug	60	0		US	Randomized, Crossover Assignment	Hospital	2015
A Study of MHAA4549A in Combination With Oseltamivir Versus Oseltamivir in Participants With Severe Influenza A Infection	Respiratory Disease	Drug	168	2	American Indian or Alaska Native	US	Randomized, Parallel Assignment	Industry	2015
Accuracy of Self-estimation of Blood Alcohol Concentration Compared to Object Values	Mental Illness	Behavioral	55		Race and Ethnicity Not Collected	US	Single Group Assignment	Hospital	2015
Adjunctive Brief Behavioral Treatment of Insomnia (BBTI) for Sleep Intervention (SI)	Mental Illness	Behavioral	54	1	American Indian or Alaska Native	US	Randomized, Parallel Assignment	Industry	2015
An Open-label Extension Study to Evaluate the Safety of the 13 mg Bimatoprost Ocular Insert	Hypertension	Drug	81		Race and Ethnicity Not Collected	US	Single Group Assignment	Industry	2015
Assessing Knowledge of Dietary Sodium Content and Implementation of Color-Coded Cue Cards and Dietary Sodium	Cardiovascular Disease	Behavioral	10	0		US	Single Group Assignment	University	2015
Cancer Associated Thrombosis and Isoquercetin (CATIQ)	Cardiovascular Disease	Drug	64	0		US	Non-randomized, Sequential Assignment	Hospital	2015
Cardiogoniometry for Detecting Coronary Artery Disease by CT Angiography	Cardiovascular Disease	Device	2	0		US	Single Group Assignment	University	2015
Connection to Care: Pilot Study of a Mobile Health Tool for Patients With Depression and Anxiety	Mental Illness	Behavioral	18	0		US	Single Group Assignment	University	2015
Coordinated Healthcare Interventions for Childhood Asthma Gaps in Outcomes	Respiratory Disease	Behavioral	373	2	American Indian or Alaska Native	US	Randomized, Parallel Assignment	University	2015
Corticosteroid/Ropivacaine Versus Corticosteroid/Saline Injections for Knee Osteoarthritis	Arthritis	Drug	29		Race and Ethnicity Not Collected	US	Randomized, Parallel Assignment	Hospital	2015
Cranial Electrotherapy Stimulation (CES) Therapy	Mental Illness	Device	6		Race and Ethnicity Not Collected	US	Single Group Assignment	University	2015
Education Bundle to Decrease Patient Refusal of VTE Prophylaxis	Cardiovascular Disease	Behavioral	19652	21	Native American	US	Non-randomized, Parallel Assignment	University	2015

Effect of Harvoni on Proteinuria and eGFR in Hepatitis C Virus Associated Chronic Kidney Disease (CKD)	Kidney Disease	Drug	14	0		US	Single Group Assignment	Hospital	2015
Effect of LIK066 on Body Weight in Patients With Elevated Body Mass Index	Diabetes	Drug	181		Race and Ethnicity Not Collected	US	Randomized, Parallel Assignment	Industry	2015
Effect of Methylnaltrexone on the PK/PD Profiles of Ticagrelor in Patients Treated With Morphine	Cardiovascular Disease	Drug	30	0		US	Randomized, Crossover Assignment	University	2015
Efficacy and Safety of Sotagliflozin in Young Adult Patients With Type 1 Diabetes Mellitus and Elevated Hemoglobin A1C	Diabetes	Drug	87	0		US	Randomized, Parallel Assignment	Industry	2015
Efficacy of Quetiapine for Pediatric Delirium	Mental Illness	Drug	6	0		US	Randomized, Parallel Assignment	University	2015
Efficacy of Two Experimental Oral Rinses in Providing Long Term Relief From Dentinal Hypersensitivity	Dental Disease	Device	240		Race and Ethnicity Not Collected	US	Randomized, Parallel Assignment	Industry	2015
Evaluation of an Ascensia Lancing System	Diabetes	Device	119	3	American Indian or Alaska Native	US	Single Group Assignment	Industry	2015
Evaluation of Safety and Efficacy of Lumason in Pediatric Echocardiography	Cardiovascular Disease	Drug	13	0		US	Single Group Assignment	Industry	2015
Improving Mood in Veterans in Primary Care	Mental Illness	Behavioral	140	4	Native Hawaiian or other Pacific Islander	US	Randomized, Parallel Assignment	Research Institute	2015
Improving Patient-Centered Care Delivery Among Patients With Chronic Obstructive Pulmonary Disease	Respiratory Disease	Other	240	4	American Indian or Alaska Native	US	Randomized, Parallel Assignment	University	2015
Injectable Pharmacotherapy for Opioid Use Disorders (IPOD)	Mental Illness	Drug	151	13	American Indian or Alaska Native	US	Randomized, Parallel Assignment	University	2015
Integrated Tele-monitoring and Patient-centric Health Coaching Strategy in Patients Hospitalized With Heart Failure	Cardiovascular Disease	Device	112	0		US	Randomized, Parallel Assignment	Hospital	2015
Intranasal Glulisine in Amnestic Mild Cognitive Impairment and Probable Mild Alzheimer's Disease	Mental Illness	Drug	49	0		US	Randomized, Parallel Assignment	Research Institute	2015
Lidocaine for Diabetic Peripheral Neuropathy	Diabetes	Drug	34	0		US	Randomized, Crossover Assignment	University	2015
Long-Term Extension Study of KRN23 in Adult Subjects With X-Linked Hypophosphatemia (XLH)	Kidney Disease	Biological	20	0		US	Single Group Assignment	Industry	2015
Lorvotuzumab Mertansine in Treating Younger Patients With Relapsed or Refractory Wilms Tumor, Rhabdomyosarcoma, Neuroblastoma, Pleuropulmonary Blastoma, Malignant Peripheral Nerve Sheath Tumor, or Synovial Sarcoma	Kidney Disease	Other	62	0		US	Single Group Assignment	NIH	2015

Medtronic Resolute Onyx 2.0 mm Clinical Study	Cardiovascular Disease	Device	101	1	American Indian or Alaska Native	US	Single Group Assignment	Industry	2015
Minocycline Augmentation to Clozapine	Mental Illness	Drug	10		Race and Ethnicity Not Collected	US	Non-randomized, Parallel Assignment	University	2015
Mirabegron in Parkinson Disease and Impaired Cognition	Mental Illness	Drug	7		Race and Ethnicity Not Collected	US	Randomized, Parallel Assignment	Research Institute	2015
Multiple Ascending Doses of PF-04958242 in Subjects With Stable Schizophrenia	Mental Illness	Drug	39		Race and Ethnicity Not Collected	US	Randomized, Parallel Assignment	Industry	2015
Non-invasive Measurement of Cerebral Dynamic Autoregulation	Cardiovascular Disease	Device	21		Race and Ethnicity Not Collected	US	Single Group Assignment	University	2015
OSA Screen Negative With Spinal Duramorph	Mental Illness	Device	3	0		US	Single Group Assignment	University	2015
PACT for Individuals With Serious Mental Illness	Mental Illness	Other	331	5	American Indian or Alaska Native, Native Hawaiian or Other Pacific Islander	US	Randomized, Parallel Assignment	Research Institute	2015
PD and Safety of TG-0054 Combined With G-CSF in Multiple Myeloma, Non-Hodgkin Lymphoma and Hodgkin Disease Patients	Cardiovascular Disease	Drug	12	1	Native Hawaiian or Other Pacific Islander	US	Single Group Assignment	Industry	2015
Pharmacokinetic Comparison Of All FK-506 Formulations	Kidney Disease	Drug	31	0		US	Randomized, Crossover Assignment	Industry	2015
Pharmacokinetics of Amlodipine Besylate at Delivery and During Lactation	Hypertension	Drug	16	0		US	Single Group Assignment	University	2015
Pharmacokinetics, Pharmacodynamics, and Impact of Inorganic Nitrate on Exercise in HFpEF	Cardiovascular Disease	Drug	12	1	Pacific Islander	US	Randomized, Parallel Assignment	University	2015
Phase 2a RDEA3170 and Allopurinol Combination Study in Gout Subjects	Arthritis	Biological	41	0		US	Randomized, Parallel Assignment	Industry	2015
Phenytoin for Memory Impairment Secondary to Megestrol	Mental Illness	Drug	21	0		US	Randomized, Crossover Assignment	University	2015
Photodynamic Therapy (PDT) With Levulan and Blue Light for the Treatment of Actinic Cheilitis	Dental Disease	Drug	24	0		US	Single Group Assignment	Industry	2015
PowerUp for Health: A Diabetes Prevention Program for Men	Diabetes	Behavioral	29		Race and Ethnicity Not Collected	US	Single Group Assignment	NIH	2015

Renal Denervation Using the Vessix Renal Denervation System for the Treatment of Hypertension (REDUCE HTN:REINFORCE)	Hypertension	Device	51	0		US	Randomized, Parallel Assignment	Industry	2015
Role of Magnesium Supplementation in the Treatment of Depression	Mental Illness	Drug	126	0		US	Randomized, Crossover Assignment	University	2015
rtPA in in the Prevention of CVAD-Associated Thrombosis and Infection in Pediatric Patients With Short Bowel Syndrome	Cardiovascular Disease	Drug	8	0		US	Single Group Assignment	University	2015
Safety and Efficacy Study of NBI-98854 in Adults With Tourette Syndrome	Mental Illness	Drug	124	0		US	Randomized, Parallel Assignment	Industry	2015
SGLT2 Inhibition and Stimulation of Endogenous Glucose Production: Protocol 2	Diabetes	Drug	30	0		US	Randomized, Parallel Assignment	NIH	2015
SMART-SF Radiofrequency Ablation Safety Study	Cardiovascular Disease	Device	165	1	Native Hawaiian or other Pacific Islander	US	Single Group Assignment	Industry	2015
Study of Ixazomib With Pegylated IFN-alpha 2b (pIFN) in Metastatic Renal Cell Carcinoma (mRCC)	Kidney Disease	Drug	3		Race and Ethnicity Not Collected	US	Single Group Assignment	Industry	2015
Tack Optimized Balloon Angioplasty Study of the Tack Endovascular Systemâ„¢ in Femoropoliteal Arteries	Cardiovascular Disease	Device	213	0		US	Single Group Assignment	Industry	2015
TactiCathÂ® Contact Force Ablation Catheter Study for Atrial Fibrillation Post Approval Study	Cardiovascular Disease	Device	178	0		US	Single Group Assignment	Industry	2015
Telerehabilitation in the Home Versus Therapy In-Clinic for Patients With Stroke	Cardiovascular Disease	Device	124	0		US	Randomized, Parallel Assignment	University	2015
The Combination Ambrisentan Plus Spironolactone in Pulmonary Arterial Hypertension Study	Hypertension	Drug	2		Race and Ethnicity Not Collected	US	Randomized, Crossover Assignment	Hospital	2015
The Effect of Aromatherapy on Neonatal Abstinence Syndrome and Salivary Cortisol Levels	Mental Illness	Drug	38		Race and Ethnicity Not Collected	US	Randomized, Parallel Assignment	University	2015
The Effect of Healing Touch on Sleep	Mental Illness	Behavioral	41	0		US	Single Group Assignment	Hospital	2015
Thoracoscopic Lung Cancer Staging With the Use of Intraoperative Ultrasound	Respiratory Disease	Device	9	0		US	Single Group Assignment	University	2015
Three-way, Cross-over Closed-loop Exercise Study	Diabetes	Device	23		Race and Ethnicity Not Collected	US	Randomized, Crossover Assignment	University	2015
To Evaluate Safety, Pharmacokinetics and Pharmacodynamics of MEDI6012 in Subjects With Stable Coronary Artery Disease	Cardiovascular Disease	Biological	48		Race and Ethnicity Not Collected	US	Randomized, Sequential Assignment	Industry	2015
Treatment of Young Adults With Comorbid AUD/MDD: A Pilot Medication Trial	Mental Illness	Drug	7		Race and Ethnicity Not Collected	US	Randomized, Parallel Assignment	University	2015

Trial To Assess The Safety And Tolerability Of Lucinactant For Inhalation In Preterm Neonates 26 to 28 Weeks PMA	Respiratory Disease	Drug	48	0		US	Randomized, Sequential Assignment	Industry	2015
University of California, San Diego (UCSD) Suramin Autism Treatment-1 (SAT1) Trial	Mental Illness	Drug	10	0		US	Randomized, Parallel Assignment	University	2015
Valproic Acid for Treatment of Hyperactive or Mixed Delirium in ICU	Mental Illness	Drug	3	1	Native Hawaiian or Other Pacific Islander	US	Randomized, Parallel Assignment	University	2015
Verapamil for Beta Cell Survival Therapy in Type 1 Diabetes	Diabetes	Drug	24	0		US	Randomized, Parallel Assignment	University	2015
Vortioxetine, 5, 10, and 20 mg, Relapse Prevention Study in Adults With Major Depressive Disorder (MDD)	Mental Illness	Drug	1106	17	American Indian or Alaska Native, Native Hawaiian or Other Pacific Islander	US	Randomized, Parallel Assignment	Industry	2015
Vulvoscopy Changes of the Vulva, Vestibule and Vagina With Daily Ospemifene in Women With Dyspareunia From VVA	Mental Illness	Drug	8	0		US	Single Group Assignment	Industry	2015
Wellness Programs for Brain-Injured Individuals	Mental Illness	Behavioral	37	0		US	Randomized, Parallel Assignment	Research Institute	2015
12-Week Study of DS-8500a in Subjects With Type 2 Diabetes Mellitus on Metformin	Diabetes	Drug	298	4	American Indian or Alaska Native	US	Randomized, Parallel Assignment	Industry	2016
A Phase 1 Study of Continuous Intravenous L-citrulline During Sickle Cell Pain Crisis or Acute Chest Syndrome	Respiratory Disease	Drug	4		Race and Ethnicity Not Collected	US	Single Group Assignment	University	2016
A Phase 1b Study of MEDI4920 in Participants With Adult-onset Rheumatoid Arthritis	Arthritis	Drug	57	0		US	Randomized, Parallel Assignment	Industry	2016
A Study of ASP8273 vs. Erlotinib or Gefitinib in First-line Treatment of Patients With Stage IIIB/IV Non-small Cell Lung Cancer Tumors With EGFR Activating Mutations	Respiratory Disease	Drug	530	4	American Indian or Alaska Native	US	Randomized, Parallel Assignment	Industry	2016
A Study of L-DOPA for Depression and Slowing in Older Adults	Mental Illness	Drug	47	0		US	Single Group Assignment	Hospital	2016
A Study of the Effect of XmAbÂ®5871 in Patients With Systemic Lupus Erythematosus	Arthritis	Biological	105	3	American Indian or Alaska Native	US	Randomized, Parallel Assignment	Industry	2016
A Study to Evaluate the Efficacy and Safety of Dasotraline in Children 6 to 12 Years of Age With Attention-Deficit Hyperactivity Disorder (ADHD) in a Simulated Classroom Setting.	Mental Illness	Drug	132	1	Native Hawaiian or Other Pacific Islander	US	Randomized, Parallel Assignment	Industry	2016

A Study To Evaluate The Safety And Efficacy Of PF-04958242 In Subjects With Cognitive Impairment Associated With Schizophrenia (CIAS)	Mental Illness	Drug	35		Race and Ethnicity Not Collected	US	Randomized, Parallel Assignment	Industry	2016
Adductor Canal Block Versus Periarticular Bupivicaine Injection in Total Knee Arthroplasty	Arthritis	Drug	155		Race and Ethnicity Not Collected	US	Randomized, Parallel Assignment	University	2016
An Open Label Trial of Bupropion and Naltrexone for Binge Drinking	Mental Illness	Drug	12	0		US	Single Group Assignment	University	2016
Andecaliximab as Add-On Therapy to a Tumor Necrosis Factor Inhibitor and Methotrexate Regimen in Adults With Moderately to Severely Active Rheumatoid Arthritis	Arthritis	Drug	15	0		US	Randomized, Parallel Assignment	Industry	2016
Anti-Mesothelin Antibody Drug Conjugate Anetumab Ravtansine for Mesothelin Expressing Lung Adenocarcinoma	Respiratory Disease	Drug	2	0		US	Non-randomized, Sequential Assignment	Research Institute	2016
Buspirone in Parkinson's Disease	Mental Illness	Drug	21	0		US	Randomized, Parallel Assignment	University	2016
Cellular Immunotherapy for Viral Induced Cancer - EBV Positive Lymphomas	Cardiovascular Disease	Biological	1	0		US	Single Group Assignment	Industry	2016
Clinical Trial of NAC in Asthma	Respiratory Disease	Drug	1	0		US	Randomized, Crossover Assignment	University	2016
Cognitive Behavior Therapy for Work Success in Veterans With Mental Illness: A Pre-post Efficacy Study	Mental Illness	Behavioral	57	0		US	Single Group Assignment	Research Institute	2016
Comparative Effectiveness of Decision Aids for Stable Chest Discomfort	Cardiovascular Disease	Behavioral	34	0		US	Randomized, Parallel Assignment	Hospital	2016
Comparative Effectiveness of Decision Support Strategies for Joint Replacement Surgery	Arthritis	Behavioral	1220	3	American Indian or Alaska Native, Native Hawaiian or Other Pacific Islander	US	Randomized, Factorial Assignment	Hospital	2016
Double-masked Study of PG324 Ophthalmic Solution in Patients With Open-angle Glaucoma or Ocular Hypertension	Hypertension	Drug	750	1	American Indian or Alaska Native	US	Randomized, Parallel Assignment	Industry	2016
Efficacy of Three Toothpastes Using an in Situ Caries Model	Dental Disease	Drug	45	0		US	Randomized, Crossover Assignment	Industry	2016
ENhancing Outcomes Through Goal Assessment and Generating Engagement in Diabetes Mellitus	Diabetes	Behavioral	1362		Race and Ethnicity Not Collected	US	Randomized, Parallel Assignment	Industry	2016
Evaluation of Web-Based CBT for Women Veterans With PTSD	Mental Illness	Behavioral	102	5	American Indian or Alaska Native, Native	US	Randomized, Parallel Assignment	Research Institute	2016

					Hawaiian or Other Pacific Islander				
Exploratory Trial to Assess the Functionality of an Integrated Call Center for the Digital Medicine System	Mental Illness	Drug	49	0		US	Single Group Assignment	Industry	2016
FAMS Mobile Health Intervention for Diabetes Self-care Support	Diabetes	Behavioral	512	5	American Indian or Alaska Native, Native Hawaiian or Other Pacific Islander	US	Randomized, Parallel Assignment	University	2016
Feasibility of Home-based Virtual Reality Rehabilitation for the Upper Extremity in Subacute and Chronic Stroke	Cardiovascular Disease	Device	20	0		US	Single Group Assignment	University	2016
Hyperbaric Oxygen Therapy as Adjunctive Therapy to Scaling and Root-planing in the Management of Periodontitis in Patients With Type 2 Diabetes	Diabetes	Device	3	0		US	Non-randomized, Parallel Assignment	University	2016
Investigation Into the Effects of Blood Glucose Levels Upon Eating Behavior in Lean and Obese Non-diabetic and Diabetic Subjects	Diabetes	Behavioral	31	0		US	Non-randomized, Parallel Assignment	NIH	2016
Involving Family to Improve Communication in Primary Care	Mental Illness	Other	93	0		US	Randomized, Parallel Assignment	University	2016
Iron Isomaltoside/Ferric Derisomaltose vs Iron Sucrose for Treatment of Iron Deficiency Anemia in Non-Dialysis-Dependent Chronic Kidney Disease	Kidney Disease	Drug	1538	5	American Indian or Alaska Native, Native Hawaiian or Other Pacific Islander	US	Randomized, Parallel Assignment	Industry	2016
Liposomal Bupivacaine for Pain Control After Total Shoulder Arthroplasty	Arthritis	Drug	108	0		US	Randomized, Parallel Assignment	University	2016
Methohexital v Propofol as General Anesthetic in Patients on ACEIs or ARBs	Cardiovascular Disease	Drug	51		Race and Ethnicity Not Collected	US	Randomized, Parallel Assignment	Hospital	2016
Milrinone in Addition to Hyperdynamic Therapy in the Treatment of Vasospasm Following Aneurysmal Subarachnoid Hemorrhage	Cardiovascular Disease	Drug	4	0		US	Randomized, Parallel Assignment	University	2016
Modulating Interaction of Motor Learning Networks in Rehabilitation of Stroke	Cardiovascular Disease	Device	10	0		US	Randomized, Crossover Assignment	NIH	2016
NeoSync TMS Treatment for Bipolar I Depression	Mental Illness	Device	6	0		US	Single Group Assignment	Hospital	2016

Novel Methods for Ascertainment of Gout Flares -A Pilot Study	Arthritis	Device	44	0		US	Randomized, Crossover Assignment	University	2016
OHI--Randomized Control Trial to Evaluate Efficacy, Acceptability, and Perception of Benefit of an Innovative Custom AFO	Cardiovascular Disease	Device	44	0		US	Randomized, Parallel Assignment	University	2016
Physical Activity Behavior Change for Older Adults After Dysvascular Amputation	Diabetes	Behavioral	31	2	American Indian or Alaska Native	US	Randomized, Crossover Assignment	Research Institute	2016
Probiotics for Quality of Life in Autism Spectrum Disorders	Mental Illness	Drug	13	0		US	Randomized, Crossover Assignment	University	2016
Pulmonary Artery Pressure Reduction With ENTresto (Sacubitril/Valsartan)	Cardiovascular Disease	Device	4	0		US	Randomized, Parallel Assignment	Hospital	2016
Remote Surveillance of Postpartum Hypertension	Hypertension	Other	206	0		US	Randomized, Parallel Assignment	University	2016
Ridge Preservation Following Tooth Extraction Using Porcine and Bovine Xenograft Materials	Dental Disease	Device	44	0		US	Randomized, Parallel Assignment	University	2016
Safety and Efficacy of BL-8040 for the Mobilization of Donor Hematopoietic Stem Cells and Allogeneic Transplantation in Patients With Advanced Hematological Malignancies	Cardiovascular Disease	Drug	50	0		US	Non-randomized, Parallel Assignment	University	2016
Stopping Cavities Study: Diammine Silver Fluoride	Dental Disease	Drug	64	2	American Indian or Alaska Native	US	Randomized, Parallel Assignment	Industry	2016
Study Comparing Daratumumab, Lenalidomide, Bortezomib, and Dexamethasone (D-RVd) Versus Lenalidomide, Bortezomib, and Dexamethasone (RVd) in Subjects With Newly Diagnosed Multiple Myeloma	Cardiovascular Disease	Drug	224	0		US	Randomized, Parallel Assignment	Research Institute	2016
Study of TAK-071 in Healthy Participants and Participants With Mild Cognitive Impairment/Mild Alzheimer Disease and Relative Bioavailability (BA) and Food Effect of TAK-071 in Healthy Participants	Mental Illness	Drug	179	4	Native Hawaiian or Other Pacific Islander	US	Randomized, Parallel Assignment	Industry	2016
Study of the Pan-DAC Inhibitor AR-42 and Pazopanib in Advanced Sarcoma and Kidney Cancer	Kidney Disease	Drug	6	0		US	Non-randomized, Sequential Assignment	NIH	2016
Study to Assess Efficacy and Safety of HP3070 in Subjects Diagnosed With Schizophrenia.	Mental Illness	Drug	617	1	American Indian or Alaska Native	US	Randomized, Parallel Assignment	Industry	2016
Study to Assess the Effects of FX006 on Blood Glucose in Patients With OA of the Knee and Type 2 Diabetes	Diabetes	Drug	33		Race and Ethnicity Not Collected	US	Randomized, Parallel Assignment	Industry	2016
Study to Evaluate the Efficacy and Safety of Adjunctive Pimavanserin in Major Depressive Disorder (CLARITY)	Mental Illness	Drug	207	4	American Indian or Alaska Native	US	Randomized, Parallel Assignment	Industry	2016

Technology Assisted Programs That Promote Mental Health for Teenagers	Mental Illness	Behavioral	40			US	Non-randomized, Parallel Assignment	University	2016
The iLet Introduction Study: A Feasibility Study of the iLet, a Fully Integrated Bihormonal Bionic Pancreas	Diabetes	Device	20	0		US	Randomized, Crossover Assignment	Hospital	2016
The Use of Mini-dose Glucagon to Prevent Exercise-induced Hypoglycemia in Type 1 Diabetes	Diabetes	Other	15	0		US	Randomized, Crossover Assignment	Research Institute	2016
ToRsemide for pOstpartum HYpertension	Hypertension	Drug	118	0		US	Randomized, Parallel Assignment	University	2016
Trial to Evaluate the Effect of ALN-PCSSC Treatment on Low Density Lipoprotein Cholesterol (LDL-C)	Cardiovascular Disease	Drug	501	7	American Indian or Alaska Native, Native Hawaiian or Pacific Islander	US	Randomized, Parallel Assignment	Industry	2016
Use of Lexiscan for Myocardial Stress Perfusion Computed Tomography With a 3rd Generation Dual Source CT System	Cardiovascular Disease	Drug	24			US	Single Group Assignment	University	2016
Virtual Reality Training for Social Skills in Schizophrenia	Mental Illness	Behavioral	47	1	American Indian or Alaska Native	US	Randomized, Parallel Assignment	University	2016
A Comparison of Methods of Discontinuing Nasal CPAP in Premature Infants <30 Weeks Gestation	Respiratory Disease	Device	66		Race and Ethnicity Not Collected	US	Randomized, Parallel Assignment	University	2017
A Study of Pimavanserin for the Treatment of Agitation and Aggression in Subjects With Alzheimer's Disease	Mental Illness	Drug	78	0		US	Single Group Assignment	Industry	2017
A Study of the Effectiveness and Efficacy of the PowerSleep Device	Mental Illness	Device	84	0		US	Randomized, Crossover Assignment	Industry	2017
A Study to Assess the Analgesic Efficacy and Safety of ASP8062 in Subjects With Fibromyalgia	Arthritis	Drug	183	3	American Indian or Alaska Native	US	Randomized, Parallel Assignment	Industry	2017
A Study to Assess the PK and Safety of PT010 in Subjects With COPD Following Single and Repeat Dose	Respiratory Disease	Drug	30	0		US	Single Group Assignment	Industry	2017
A Study to Evaluate Efficacy and Safety of Anakinra in the Treatment of Still's Disease (SJIA and AOSD)	Arthritis	Biological	12	0		US	Randomized, Parallel Assignment	Industry	2017
Assessment of Glycemic Control in Patients With Type 2 Diabetes Mellitus and Late Stage Chronic Kidney Disease	Diabetes	Device	80	1	American Indian or Alaska Native	US	Single Group Assignment	Research Institute	2017
Carfilzomib With or Without Rituximab in the Treatment of Waldenstrom Macroglobulinemia or Marginal Zone Lymphoma	Cardiovascular Disease	Drug	4	0		US	Single Group Assignment	NIH	2017

Continuous Glucose Monitors to Regulate Glucose Levels in Type 2 Diabetics	Diabetes	Behavioral	358		Race and Ethnicity Not Collected	US	Single Group Assignment	Industry	2017
Daily Step-based Exercise Using Fitness Monitors for Peripheral Artery Disease	Cardiovascular Disease	Behavioral	20		Race and Ethnicity Not Collected	US	Randomized, Parallel Assignment	University	2017
Danirixin Dose Ranging Study in Participants With Chronic Obstructive Pulmonary Disease (COPD)	Respiratory Disease	Drug	614	1	Native Hawaiian or other Pacific Islander	US	Randomized, Parallel Assignment	Industry	2017
Efficacy and Safety of SHP465 at 6.25 mg in the Treatment of Attention-Deficit/Hyperactivity Disorder (ADHD) in Children Aged 6-12 Years	Mental Illness	Drug	89	1	American Indian or Alaska Native	US	Randomized, Parallel Assignment	Industry	2017
Efficacy of Glucagon In the Prevention of Hypoglycemia During Mild Exercise	Diabetes	Drug	22	0		US	Randomized, Crossover Assignment	Hospital	2017
Emergence Agitation and Pain Scores in Pediatrics When Comparing Single-modal vs Multi-modal Analgesia for ENT Surgery	Mental Illness	Drug	143	0		US	Randomized, Parallel Assignment	Hospital	2017
Evaluation of Coronary Artery Calcification Using Gated Stationary Chest Tomosynthesis	Cardiovascular Disease	Device	10	0		US	Single Group Assignment	University	2017
Hemodynamic Effects of Blood Flow Variation in Continuous Renal Replacement Therapy	Kidney Disease	Device	6		Race and Ethnicity Not Collected	US	Single Group Assignment	University	2017
Human Laboratory Study of Varenicline for Alcohol Use Disorder	Mental Illness	Drug	47	0		US	Randomized, Parallel Assignment	NIH	2017
Management of Platelet Transfusion Therapy in Patients With Blood Cancer or Treatment-Induced Thrombocytopenia	Cardiovascular Disease	Biological	4	0		US	Randomized, Parallel Assignment	NIH	2017
Multiple Ascending Doses of MEDI6012 in Subjects With Stable Atherosclerotic Cardiovascular Disease	Cardiovascular Disease	Drug	32	0		US	Randomized, Parallel Assignment	Industry	2017
Neoadjuvant Pembrolizumab	Respiratory Disease	Drug	35	0		US	Single Group Assignment	University	2017
New Approaches to Smoking Cessation in Heavy Drinkers	Mental Illness	Drug	26	0		US	Randomized, Parallel Assignment	NIH	2017
Oral Nitrite for Older Heart Failure With Preserved Ejection Fraction	Cardiovascular Disease	Drug	15		Race and Ethnicity Not Collected	US	Randomized, Parallel Assignment	NIH	2017
Paradoxical Stimulation of Hepatic Glucose Production With Dapagliflozin	Diabetes	Drug	30	0		US	Randomized, Parallel Assignment	University	2017
PTC Study to Evaluate Ataluren in Combination With Ivacaftor	Respiratory Disease	Drug	1	0		US	Single Group Assignment	University	2017
Single Agent Chemotherapy +/- Nivolumab in Patients With Advanced Squamous or Non-squamous NSCLC With Primary Resistance to Prior PD-1 or PDL-1 Inhibitor	Respiratory Disease	Drug	3	0		US	Randomized, Parallel Assignment	Research Institute	2017

Study of Naltrexone-Induced Blockade of Antidepressant Effects	Mental Illness	Drug	20		Race and Ethnicity Not Collected	US	Randomized, Crossover Assignment	University	2017
Study of PEMF to Evaluate VPT and Thermal Sensory in Subjects With Diabetic Peripheral Neuropathy	Diabetes	Device	37	0		US	Randomized, Parallel Assignment	Industry	2017
Study to Compare Palindrome vs. BioFlo DuraMax Dialysis Catheters	Kidney Disease	Device	32		Race and Ethnicity Not Collected	US	Randomized, Parallel Assignment	University	2017
The ENERGITOÂ® 2 Study Compares 2 Inhaled Medicines for Chronic Obstructive Pulmonary Disease (COPD). One Medicine is a Combination of Tiotropium and Olodaterol (StioltoÂ®) Taken Using the RespimatÂ® Inhaler and the Other Medicine is a Combination of Fluticasone and Salmeterol Taken Using the Diskus	Respiratory Disease	Drug	302	0		US	Randomized, Parallel Assignment	Industry	2017
Vitabreath Pilot in Chronic Obstructive Pulmonary Disease (COPD) Patients	Respiratory Disease	Device	25	0		US	Randomized, Crossover Assignment	Industry	2017
A Comparative Study of Electric Toothbrushes for the Efficacy Plaque Removal and the Effect on Plaque Accumulation and Gingivitis.	Dental Disease	Device	90		Race and Ethnicity Not Collected	US	Randomized, Parallel Assignment	University	2018
A Phase 2 Clinical Trial Examining the Effects on Osteoarthritic Knee Pain of CGS-200-1, CGS-200-5 and Vehicle Control	Arthritis	Drug	122	0		US	Randomized, Parallel Assignment	Industry	2018
A Study to Determine the Maximum Tolerated Dose of an Investigational Drug in Subjects With Schizophrenia	Mental Illness	Drug	40	1	Native Hawaiian or Other Pacific Islander	US	Randomized, Parallel Assignment	Industry	2018
A Study to Evaluate Efficacy and Safety of TEZ/IVA in Subjects Aged 6 Through 11 Years With Cystic Fibrosis	Respiratory Disease	Drug	67	0		US	Randomized, Parallel Assignment	Industry	2018
Brief Online Interventions for Alcohol Use	Mental Illness	Behavioral	444	0		US	Randomized, Parallel Assignment	NIH	2018
CrYobiopsy With Radial UltraSound Guidance	Respiratory Disease	Device	10	0		US	Randomized, Parallel Assignment	University	2018
Efficacy and Safety Study of a Test Naproxen Sodium 220mg Tablet in Postoperative Dental Pain	Dental Disease	Drug	501	7	American Indian or Alaska Native, Native Hawaiian or Other Pacific Islander	US	Randomized, Parallel Assignment	Industry	2018
Knee Injection RCT	Arthritis	Drug	18	0		US	Randomized, Parallel Assignment	Industry	2018

Lutonix Drug Coated Balloon for Treatment of Femoropopliteal Arteries in United States Females (CONFIRM)	Cardiovascular Disease	Device	4	0		US	Single Group Assignment	Industry	2018
Penile Lengthening Pre-Penile Prosthesis Implantation	Mental Illness	Device	24		Race and Ethnicity Not Collected	US	Non-randomized, Parallel Assignment	Hospital	2018
Study to Evaluate the Pharmacokinetics of Lemborexant (E2006) and Its Metabolites in Subjects With Normal Renal Function or With Severe Renal Impairment	Kidney Disease	Drug	16	0		US	Non-randomized, Parallel Assignment	Industry	2018
Subcutaneous Furosemide in Acute Decompensated Heart Failure: The SUBQ-HF Study	Cardiovascular Disease	Drug	11	0		US	Randomized, Parallel Assignment	University	2018
To Assess the Bioequivalence of the 4mg Prototype Mini Nicotine Lozenge to the Reference Product (Nicorette) in Healthy Smokers	Mental Illness	Drug	37	1	American Indian or Alaska Native	US	Randomized, Crossover Assignment	Industry	2018

***Indigenous Population: "Race and Ethnicity Not Collected": Refers to studies that did not collect race and ethnicity data; "Not Available": Refers to studies that we were unable to obtain and extract Indigenous enrollment data; and "Blank": Refers to studies that did not include Indigenous populations.**

Figure S1. PRISMA flow diagram of general population trials.

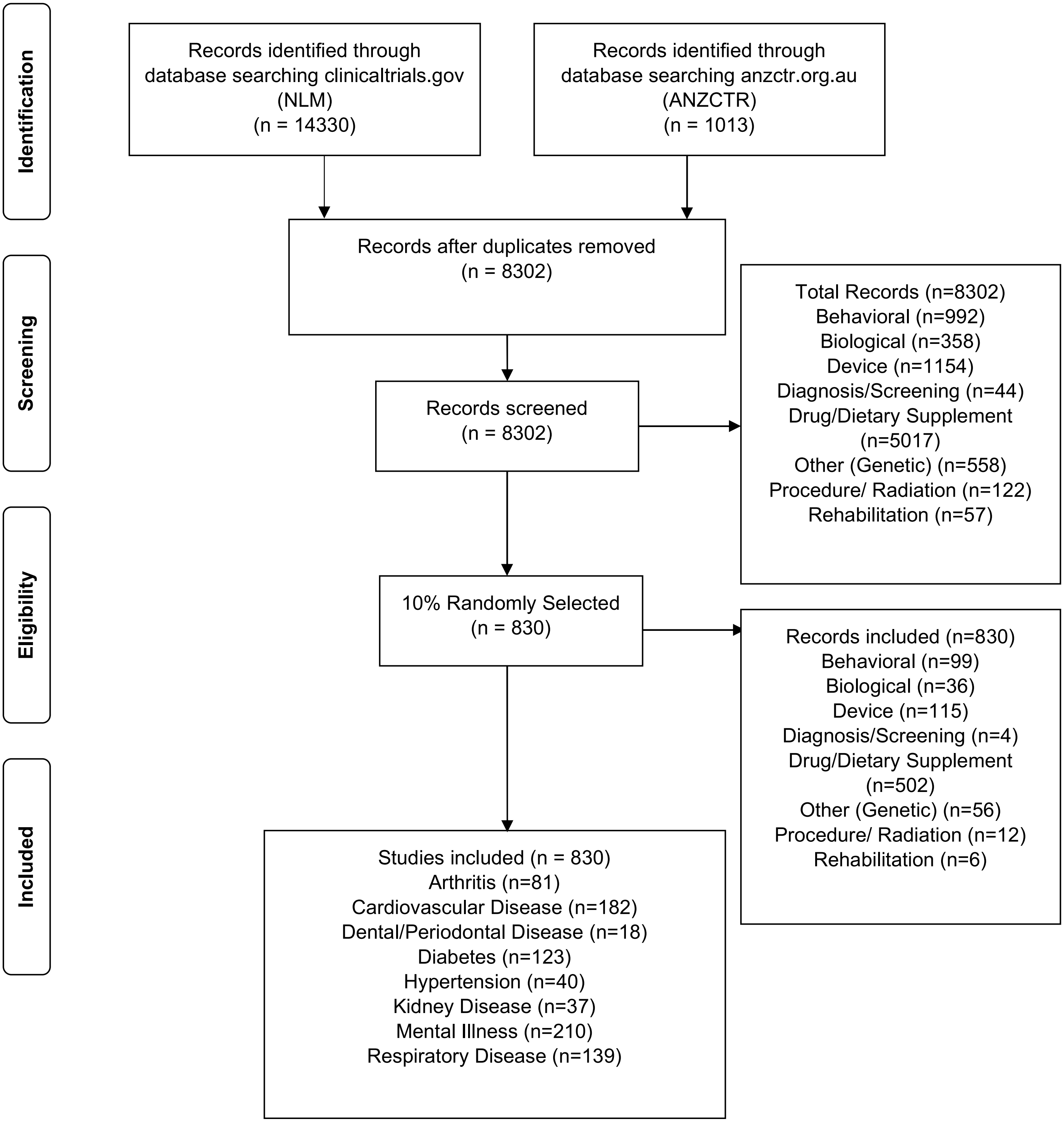

Supplement: sj-pdf-1-ctj-10.1177_17407745211069153 – Supplemental material for The representation of Indigenous peoples in chronic disease clinical trials in Australia, Canada, New Zealand, and the United States [file sj-pdf-1-ctj-10.1177_17407745211069153.pdf]
